# Host blood protein biomarkers to screen for tuberculosis disease: a systematic review and meta-analysis

**DOI:** 10.1128/jcm.00786-24

**Published:** 2024-10-24

**Authors:** Mary Gaeddert, Kerstin Glaser, Bih H. Chendi, Ayten Sultanli, Lisa Koeppel, Emily L. MacLean, Tobias Broger, Claudia M. Denkinger

**Affiliations:** 1 Department of Infectious Diseases and Tropical Medicine, Center for Infectious Diseases, Heidelberg University Hospital, Heidelberg, Germany; 2 Division of Immunology, Department of Biomedical Sciences, Faculty of Medicine and Health Sciences, South African Medical Research Council Centre for Tuberculosis Research, Stellenbosch University, Cape Town, South Africa; 3 Institute of Tropical Medicine, University of Tübingen, Tübingen, Germany; 4 German Center for Infection Research, Tübingen, Germany; 5 NHMRC Clinical Trials Centre, Faculty of Medicine and Health, The University of Sydney, Sydney, Australia; 6 German Center for Infection Research, Partner site Heidelberg, Heidelberg, Germany; University of Manitoba, Winnipeg, Canada

**Keywords:** tuberculosis, biomarker, host protein, screening

## Abstract

Non-sputum tests are needed to improve tuberculosis (TB) diagnosis and close the diagnostic gap. The World Health Organization’s target product profile (TPP) for point-of-care (POC) screening tests requires a minimum sensitivity of 90% and a specificity of 70%. Our objective was to identify host blood protein biomarkers meeting TPP criteria. A systematic review was conducted and reported following PRISMA guidelines. Data extraction and quality assessment with Quality Assessment of Diagnostic Accuracy Studies-2 (QUADAS-2) were completed for the included studies. Heterogeneity was assessed. For biomarkers reporting sensitivity and specificity in at least four studies, a random-effects meta-analysis was performed for biomarkers with similar cut-offs. We screened 4,651 citations and included 65 studies that enrolled 16,010 participants and evaluated 156 host proteins. Most (47/65) studies enrolled adult pulmonary TB (PTB), with 15 studies in adult extra-pulmonary TB and 5 in children. Small early-stage discovery studies with case-control design were common (24/65) and had a high risk of bias. For adult PTB, CRP, IP-10, NCAM-1, and SAA met TPP criteria in high-quality studies. There was a high degree of heterogeneity in biomarker cut-offs and study design. CRP at 10 mg/L cut-off was meta-analyzed from 10 studies; pooled sensitivity 86% [95% confidence interval (CI): 80–95] and pooled specificity 67% (95% CI: 54–79). In people living with HIV (six studies), CRP pooled sensitivity was 93% (95% CI: 90–95), and pooled specificity was 59% (95% CI: 40–78). We identified promising biomarkers that performed well in high-quality studies. Data overall are limited and highly heterogenous. Further standardized validation across subgroups in prospective studies is needed before translating into POC assays.

**IMPORTANCE:**

To our knowledge, this is the first comprehensive systematic review of host blood protein biomarkers for tuberculosis (TB), and we identified promising biomarkers for a TB screening test.

## INTRODUCTION

Tuberculosis (TB) was the second leading cause of death by a single infectious disease worldwide in 2022 after coronavirus disease 2019 (1). Even though the disease is curable, individuals must first be diagnosed before starting treatment, and the limitations of current TB diagnostics are one of the factors contributing to the estimated 3 million TB cases that were missed due to underdiagnosis or under-reporting in 2022 (1). With 87% of the global TB cases concentrated in only 30 high-burden countries (1), many of which have limited resources, there is a need for new diagnostics that have a wider reach.

Current diagnostics for TB disease are mainly sputum-based, and there are well-recognized challenges with obtaining adequate sputum samples for testing, particularly in people living with HIV (PLHIV) and children (2). Sputum-based methods are also unsuitable for the detection of extra-pulmonary TB (EPTB) disease (3). A molecular WHO-recommended rapid diagnostic (mWRD) was used as the initial test for 47% of the newly diagnosed TB cases in 2022, and the goal is to reach 100% coverage by 2027 (1). In order to close this gap and improve TB diagnostics globally, there is a need for non-sputum-based rapid tests that can be used in a point-of-care (POC) setting where they would reach more patients earlier in the disease course.

In 2014, the World Health Organization (WHO) issued target product profiles (TPPs) to guide the development of new TB diagnostics which included recommendations for a screening test (4). The goal is to develop a high-sensitivity test that can be used to rule out adults and children with presumptive TB at lower levels of the healthcare system. A positive result on the screening test would require a second test with higher specificity as confirmation. The minimum accuracy recommended by the TPP was sensitivity of 90% and specificity of 70%. The TPP also recommends operational characteristics to enable a better reach in resource-limited settings. This includes non-sputum samples that are easy to collect such as blood from a finger-stick, testing platforms that can be used at POC, and a low cost per test.

Host blood biomarkers could possibly meet these accuracy targets as suggested by results from a recent study on the Xpert MTB Host Response assay using a 3-gene signature (sensitivity of 90%, specificity of 63%) (5). However, tests based on mRNA targets or other genomic signatures require complex equipment and are unlikely to meet stated cost targets (6, 7). Blood protein biomarkers and biomarker signatures are more likely to translate into affordable POC tests, such as a lateral-flow assay (LFA). C-reactive protein (CRP) has been evaluated in many studies, and while it does not meet performance targets in meta-analyses, it can be measured using POC testing platforms (8, 9). The question remains whether other host blood protein biomarkers, or a combination of markers, could reach the TPP accuracy targets. Well-performing markers would also need to be measurable by POC platforms, ideally using blood from a finger-prick sample.

This systematic review focused on blood host proteins with the goal of identifying markers that meet the TPP performance criteria and could be translated to POC tests. The primary objective was to review the diagnostic accuracy of host proteins in adult pulmonary TB, and the secondary objectives were to review host proteins in adult extra-pulmonary and childhood TB.

## MATERIALS AND METHODS

### Search strategy

Studies were identified through a systematic search of the databases EMBASE, PubMed, Cochrane Controlled Trials Register, Scopus, the Web of Science, clinicaltrials.gov, and African Journals Online. The search was conducted for all studies from 1 January 2010 to 5 October 2023 with no language restrictions (Table S1). We also identified papers by reviewing the citations of included papers and relevant reviews. The systematic review protocol and search strategy are registered on PROSPERO (registration number: CRD42022298906) and followed PRISMA reporting guidelines (10). We included randomized clinical trials, cohort studies, case-control studies, and cross-sectional studies. Studies with less than 20 TB cases positive by either microbiological reference standard (MRS) or composite reference standard (CRS) were excluded.

### Index test

Studies using index tests that were able to identify proteins in blood were included. The major testing platforms are enzyme-linked immunosorbent assay (ELISA), immunoassays in general, multiplexing platforms such as Luminex and Meso Scale Discovery (MSD), and mass spectrometry. Studies that used blood samples stimulated with TB antigens were excluded as they would likely not meet the operational characteristics necessary for use at POC. While antibodies are part of the proteome, we excluded antibody tests as they have shown unreliable diagnostic accuracy, and WHO strongly recommended against their use in 2011 (11).

### Reference standard

To be considered for inclusion, adult PTB studies must have used an MRS based on culture or an mWRD on any sample (12). TB cases were those who tested positive on at least one culture or mWRD, and control groups were negative on all tests. Bacteriological confirmation is difficult for extra-pulmonary and childhood TB due to their paucibacillary nature and diagnosis often relies on a combination of symptoms, imaging, and microbiologic testing (3). Therefore, we included studies that used a CRS for the childhood and EPTB groups and extracted details of the CRS definition.

### Study selection and data extraction

The results of the literature search were exported to EndNote, and duplicates were removed.

For title and abstract screening, a sample of 5% was screened by three reviewers (M.G., K.G., and A.S.), discussed to reach concordance, and then each reviewer independently assessed the remaining results. Two reviewers (M.G. and K.G.) independently reviewed the full text and conducted data extraction; discrepancies were resolved by discussion between the two reviewers, or by decision of a third reviewer (C.M.D.). Data were extracted from eligible studies using GoogleForms. Study quality was assessed only for adult pulmonary TB studies using a modified version of the Quality Assessment of Diagnostic Accuracy Studies-2 (QUADAS-2) tool (13). QUADAS signaling questions were modified to fit the review question, and the description of questions and scoring is in Table S2. Covidence was used for full-text review and QUADAS extraction.

When studies reported results from both discovery and validation cohorts, only data from the validation cohort were extracted. For studies that reported biomarker results at multiple cut-off points, we extracted the results with higher sensitivity in order to reach the TPP goal of 90% sensitivity. We reported the results of each biomarker separately from studies that reported results of multiple biomarkers.

The most clinically relevant comparison group for a screening test is other patients who present with symptoms of presumptive TB and are later diagnosed with other respiratory diseases (ORDs), as they likely have an inflammatory process causing symptoms and will be the target population for routine use. A hierarchy of control groups was developed [Supplementary Methods (14)], and if studies used multiple control groups, results from the group with the highest clinical relevance were extracted. Biomarker signatures were also extracted. Biomarker names were harmonized, and abbreviations are listed in Table S3. All results were reported separately by HIV status and country of enrollment where information was available.

### Statistical analysis

When studies did not report 95% confidence intervals (CIs) for sensitivity and specificity, we calculated the CIs using the Wilson Score Interval. The Deeks test for funnel-plot asymmetry was performed to investigate publication bias for diagnostic test accuracy meta-analyses using the “midas, pubbias” command in Stata; a *P* value <0.10 for the slope coefficient indicates significant asymmetry (15, 16). If at least four studies reported sensitivity and specificity for adult pulmonary TB, heterogeneity was assessed by visually examining forest plots and hierarchical summary receiver operating characteristic (HSROC) plots, and underlying causes of heterogeneity in study design were investigated. A random-effects meta-analysis was done using the “meta” package in Stata for biomarkers with similar cut-offs. Stata 17 (STATA Corporation, College Station, TX, USA) was used for all analyses.

## RESULTS

### Summary of studies

**Fig 1:**
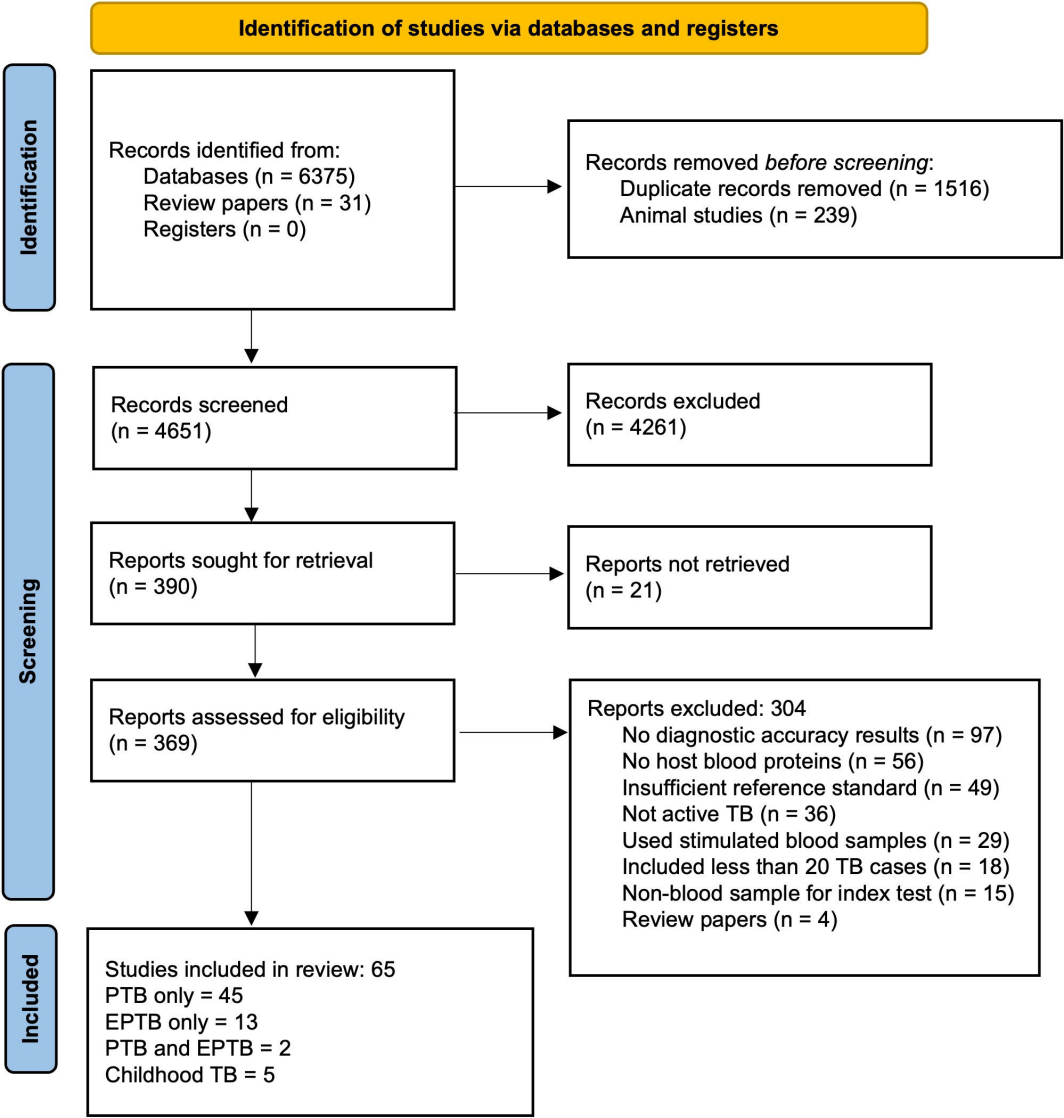
PRISMA flow diagram of study selection.

The literature search resulted in 4,651 articles after de-duplication. After title and abstract screening, 369 papers were eligible for full-text review. Of these, 304 were excluded, leaving 65 studies in the systematic review (Fig. 1); 45 provided data for adult PTB, 13 for EPTB, 2 for both PTB and EPTB, and 5 for childhood TB. The 65 studies enrolled a total of 16,010 participants from 17 different countries across all continents except Australia. Most studies enrolled participants from the regions of Southeast Asia (26/65, 40%) and Sub-Saharan Africa (24/65, 37%), with South Africa (12/65, 18%), India (12/65, 18%), and China (11/65, 17%) being the most frequent countries.

The majority of studies used serum (41/65, 63%) and plasma (19/65, 29%) for biomarker testing, and whole blood was used in four studies (6%). All studies that reported sample condition used frozen blood (44/65, 69%) except four studies (6%) that used fresh blood. Immunoassays in general (37/65, 54%), and ELISA (27/65, 39%) specifically, were the most common index test, followed by Luminex (18/65, 26%) and Turbidimetry (6/65, 9%). Mass spectrometry, MSD, nephelometry, SomaScan, the Simoa assay, and the peroxidase method were used in one study each.

### Methodological quality of studies

**Fig 2:**
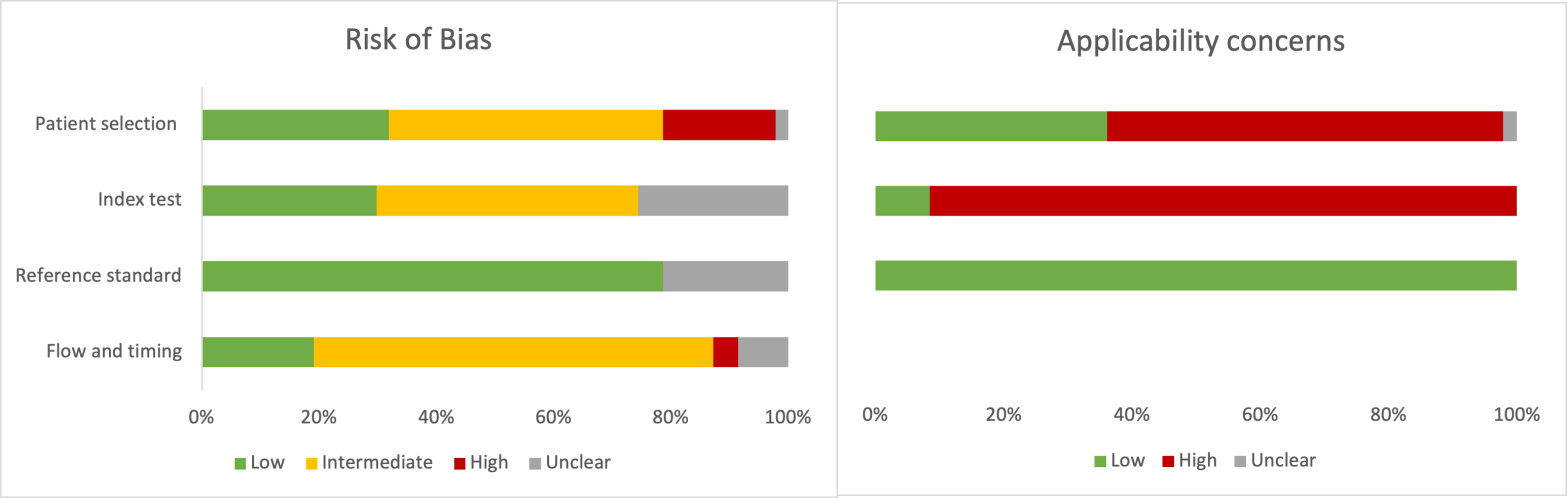
Summary of QUADAS-2 assessment for risk of bias and applicability concerns.

Many papers reviewed were early-stage discovery studies that enrolled small numbers of known TB cases and controls. Of the 47 papers that enrolled adult patients with PTB, 24 (51%) used a case-control design, resulting in a higher risk of bias in the QUADAS patient selection domain (Fig. 2). The use of a comparator group without TB symptoms resulted in a high concern of applicability for patient selection for 62% (29/47) of studies. ORD was used as the comparison in 40% (19/47) studies, and the most common non-ORD groups were LTBI (12/47) and healthy controls (9/47). Studies using healthy controls did not apply the same reference standard testing to the non-TB comparator group, increasing the risk of bias in the “flow and timing” domain. The interpretation of the index test was clearly reported as blinded from the reference standard in 26% (12/47) studies and was unclear in 72% (34/47). Cut-offs were often chosen based on the results of each study and not pre-specified or validated in other cohorts. In the domain concerning applicability of index test for POC use, only four studies (17–20) used a POC assay for CRP, so all other studies had a high concern for applicability. The inclusion criteria requiring an MRS resulted in an overall low risk of bias and low applicability concerns with respect to the reference standard. The detailed list of results by study for adult PTB is in Table S4.

The funnel plot of included adult PTB studies shows a high degree of symmetry and does not indicate substantial publication bias (*P* = 0.62) (Fig. S1).

### Summary of biomarkers

A total of 156 host proteins were evaluated across all included studies. The 47 adult PTB studies evaluated the diagnostic accuracy of 102 individual host blood proteins and 18 different signatures by calculating the sensitivity and specificity at a chosen cut-off value (Table S5 and S6). The majority of biomarkers (73/102, 72%) have results from only one study. The biomarker with the most evidence was CRP, reported by 19 studies. Some studies reported CRP performance separately by HIV status and cut-off, so there are 30 unique results included.

**Fig 3:**
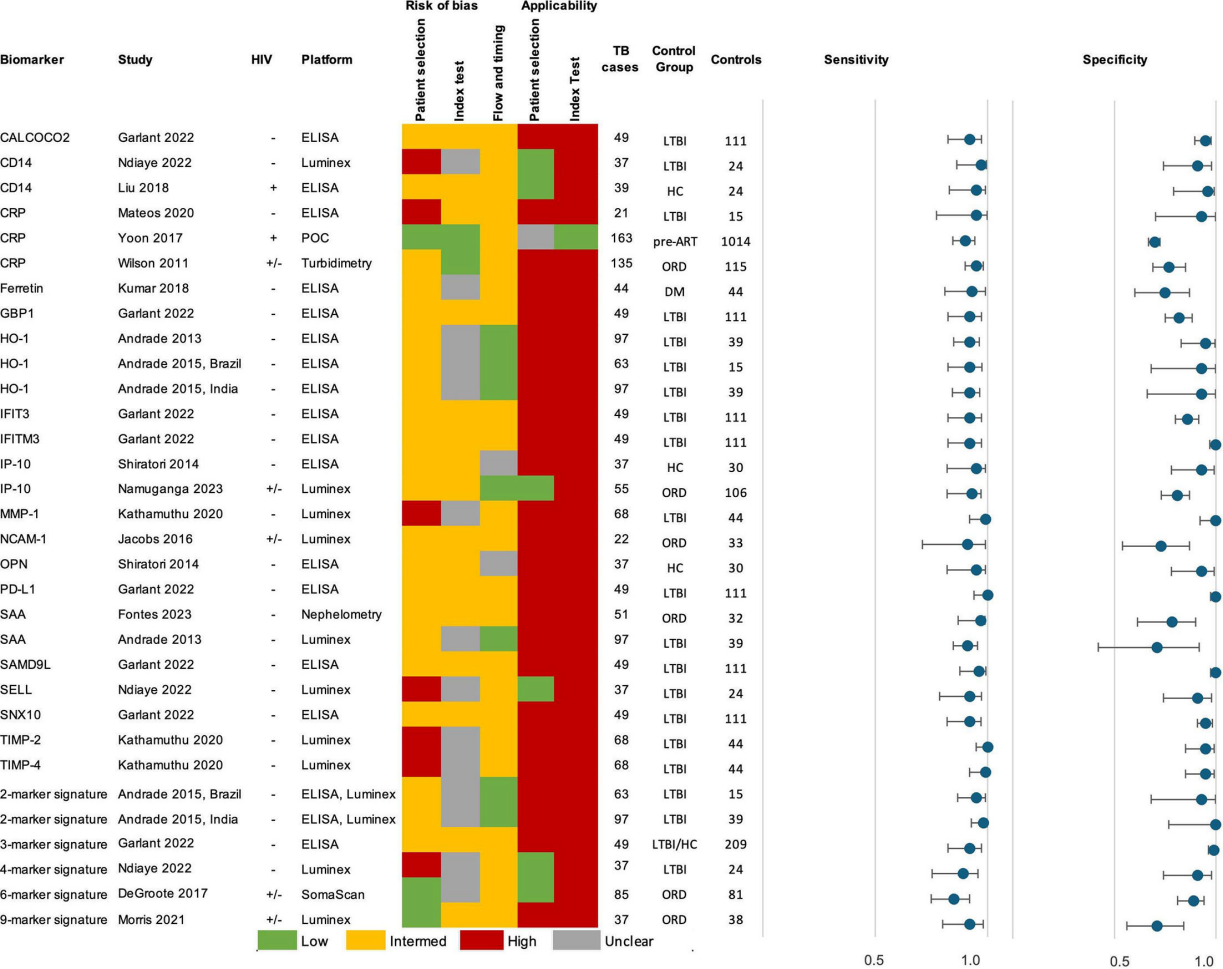
Summary of adult PTB biomarkers and signatures meeting TPP criteria. Control groups: DM = diabetes mellitus, HC = healthy control, LTBI = latent TB infection, ORD = other respiratory disease, pre-ART = before initiation of anti-retroviral therapy. 2-marker signatures: HO-1, MMP-1; 3-marker signature: CALCOCO2, IFITM3, SAMD9L; 4-marker signature: CLEC3B, ECM1, IP-10, SELL; 6-marker signature: SYWC, kallistatin, C9, gelsolin, testican-2, aldolase C; 9-marker signature: fibrinogen, α-2-M, CRP, MMP-9, TTR, CFH, IFN-γ, IP-10, TNF. The reference standard results for risk of bias and applicability were low for all studies.

**Fig 4:**
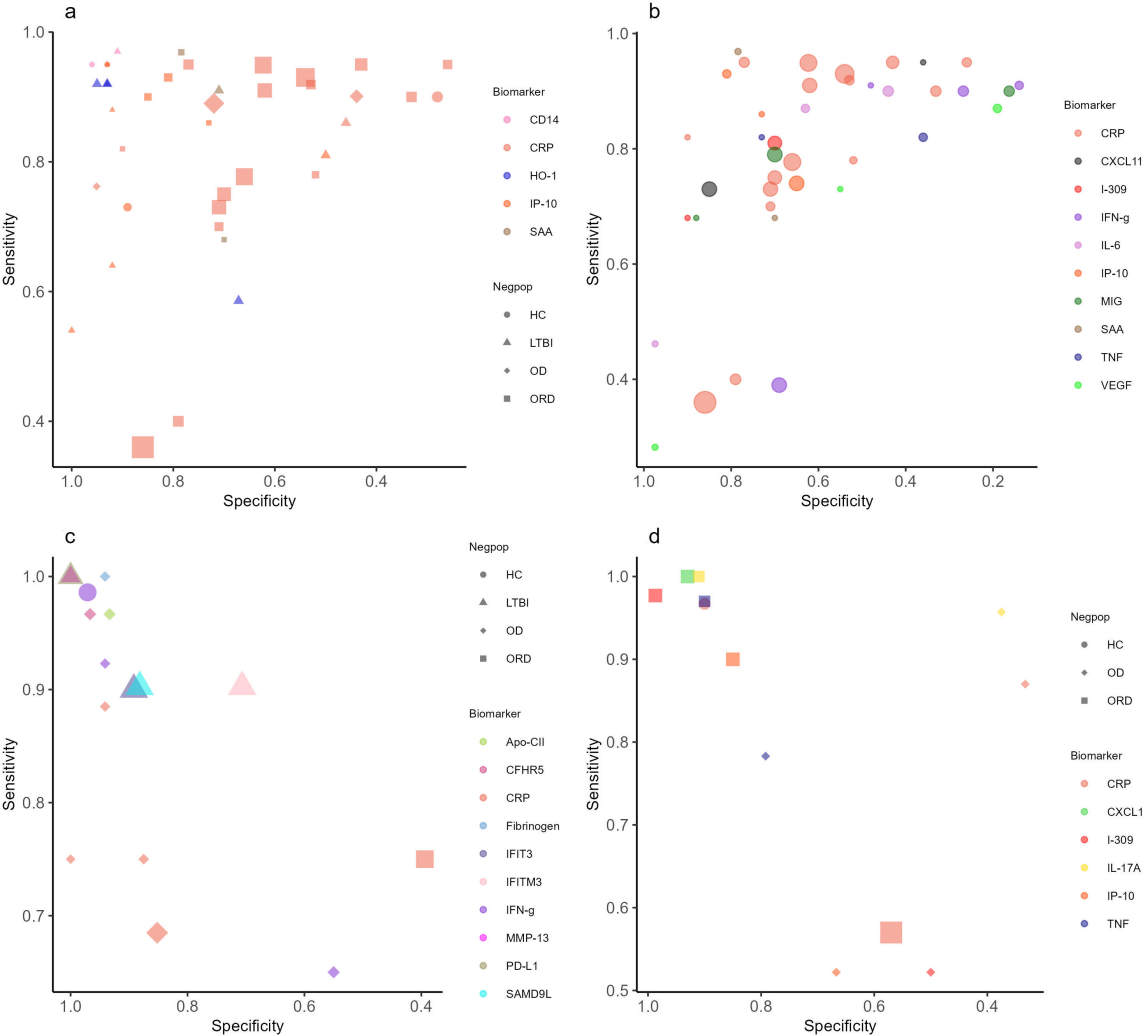
Summary plots. (a) Complete results of adult PTB biomarkers that met the TPP criteria in two or more studies. (b) Complete results of adult PTB biomarkers tested in two or more studies with limited bias for patient selection. (c) Complete results of adult extra-pulmonary TB biomarkers that met the TPP criteria in one or more studies. (d) Complete results of childhood TB biomarkers that met the TPP criteria in one or more studies. Symbol shapes represent the type of control group used as the negative population (HC = healthy control, LTBI = latent TB infection, OD = other disease, ORD = other respiratory disease). Symbol colors represent different biomarkers, and the size of the markers is proportional to the sample size. Panel (b) displays results only for biomarkers that were tested using an ORD control group, regardless of meeting TPP criteria.

In total, 19 individual biomarkers tested by 14 separate studies met the TPP criteria of sensitivity > 90% and specificity > 70% in adult PTB. Figure 3 shows the performance of each marker and the summary of study quality. Eighteen markers met TPP in HIV-negative patients: CALCOCO2 (21), CD14 (22), CRP (23), Ferritin (24), GBP1 (21), HO-1 (25, 26), IFIT3 (21), IFITM3 (21), IP-10 (18), MMP-1 (19), OPN (27), PD-L1 (21), SAA (25, 28), SAMD9L (21), SELL (22), SNX10 (21), TIMP-2 (19), and TIMP-4 (29). All of these studies had a high overall risk of bias due to the use of LTBI or healthy control groups, except one study on SAA that used an ORD comparison group (28). Furthermore, all studies choose the cut-off value which provided the highest accuracy post-hoc and did not use a pre-defined cut-off. Although these biomarkers met TPP in at least one study, there is a wide range of performance and results from other studies on the same biomarker had lower accuracy (Fig. 4a).

In studies that reported results for PLHIV, CD14 (30) and CRP (18) met the TPP criteria for performance. The CD14 study used healthy controls, and the cut-off value was chosen to reach the highest diagnostic accuracy for their cohort; the CRP study enrolled HIV-positive patients before initiation of antiretroviral therapy (ART) and chose pre-defined cut-offs. In studies with a mix of HIV-positive and HIV-negative adults, three individual biomarkers, CRP (31), IP-10 (32), and NCAM (33), met the TPP criteria in at least two of the studies. As these studies all used participants with ORD as the comparator, the risk of bias was low. Nevertheless, heterogeneity was substantial, and complete results from studies that used a clinically relevant population with a low risk of bias show that many biomarkers did not perform as well (Fig. 4b).

In addition, five biomarker signatures met the TPP criteria. A combination of HO-1 and MMP-1 using participants with LTBI as the comparator group was reported separately by sites in Brazil and India (26). A 3-marker signature of CALCOCO2, IFITM3, and SAMD9L tested lysed whole blood with ELISA, comparing TB cases in India to asymptomatic contacts from India and the United Kingdom (21). The 4-marker signature of CLEC3B, ECM1, IP-10, and SELL used Luminex to test plasma samples from TB cases and LTBI controls in Madagascar (22). The 6-marker signature by DeGroote (SYWC, kallistatin, C9, gelsolin, testican-2, and aldolase C) was developed by testing serum samples from patients with ORD with SomaScan (34). The signature’s performance was reported separately by HIV status and had an area under the curve (AUC) >0.9 for all groups; although sensitivity is slightly below 90% and specificity is above 80%. A study of a 9-marker signature consisting of fibrinogen, α-2-M, CRP, MMP-9, TTR, CFH, IFN-γ, IP-10, and TNF-α enrolled patients with ORD from South Africa and Malawi, testing serum samples on Luminex (35).

Adult PTB studies which reported only the AUC are summarized in Table S7. Of the 45 individual markers, 12 had an AUC > 0.9, which is likely to meet TPP criteria if a cut-off had been defined to calculate sensitivity and specificity: C1q (36), I-309 (37), IFN-γ (38), IL-2 (38), IL-5 (38), IL-6 (38), IL-10 (38), IL-17A (38), IL-1α (38), IP-10 (38), MIG (38), and TNF (38). All of these markers were in HIV-negative patients, and 8/12 came from one study (38) that tested both drug-susceptible and drug-resistant TB cases compared to LTBI or healthy controls. Two signatures reached an AUC of 0.9: a 2-marker combination of SYWC and I-309 in PLHIV and a 3-marker combination of I-309, SYWC, and kallistatin in a population with mixed HIV status across three countries (39).

The CRS criteria for the EPTB and childhood TB studies are listed in Table S8. Two EPTB studies (21, 37) and two childhood TB studies (40, 41) reported that all TB cases were positive on either NAAT or culture. The other studies used a combination of AFB smear or culture, and three studies (29, 42, 43) did not provide details on the reference standard.

Ten individual biomarkers met the TPP criteria for adult EPTB: fibrinogen (44) and IFN-γ (44) in a population with mixed HIV status, and Apo-CII (37), CFHR5 (37), IFIT3 (21), IFITM3 (21), IFN-γ (45), MMP-13 (29), PD-L1 (21), and SAMD9L (21) in HIV-negative patients (Fig. 4c, full results in Table S9 and S10). Two signatures met the TPP criteria for EPTB: a 5-marker signature consisting of CRP, NCAM, ferritin, IL-8, and GDF-15 in population with mixed HIV status (44) and a 2-marker signature of CFHR2 and CFHR3 in HIV-negative participants (37). There were no studies in the review that enrolled only PLHIV with EPTB.

A study by Garlant et al. tested a range of markers in both PTB and EPTB patients; IFIT3, IFITM3, PD-L1, and SAMD9L met the TPP criteria in both groups (21). Importantly, the EPTB group was defined using an MRS. Also, IFN-γ performed well in two EPTB studies with HIV-negative participants that used a CRS (44, 45) and one study by Sampath, et al. with both drug-resistant and drug-susceptible HIV-negative PTB cases (38). While the Sampath study did not report sensitivity and specificity, the AUC for IFN-γ was 0.95 in drug-susceptible and 0.94 in drug-resistant cases.

Five childhood TB studies evaluated a total of 70 host blood proteins (Fig. 4d, full results in Table S11). Four studies enrolled HIV-negative children and one study enrolled children with mixed HIV status. Two biomarkers met TPP criteria in the study using an MRS: IL-17A (41) and TNF (41). This study compared TB to ORD and enrolled a separate validation cohort to reduce the risk of bias. The remaining biomarkers meeting TPP were compared to a CRS: CRP in a study that enrolled children with both PTB and EPTB (46), and CXCL-1, I-309, and IP-10 in a study with PTB (47). Two signatures met TPP for childhood TB, a 2-marker combination of I-309 and CXCL-1 (47) in children diagnosed with a CRS and a 3-marker signature of TNF, IL-2, and IL-17A (41) using an MRS. All markers that met TPP in children also performed well in adult studies, except CXCL-1. CRP and IP-10 met TPP criteria in both groups, and I-309, IL-17A, and TNF performed well in adult studies that reported only AUC.

### Meta-analysis

Heterogeneity was assessed for biomarkers that reported sensitivity and specificity in at least four studies for adult pulmonary TB (CRP, HO-1, IFN-γ, IL-6, IP-10, MIG, TNF, and VEGF). Due to the high level of heterogeneity for clear reasons like study design, population included, index test, sample type, and cut-off value, but also for additional reasons (as visualized in forest plots and HSROC curves) a meta-analysis was only possible for CRP.

**Fig 5:**
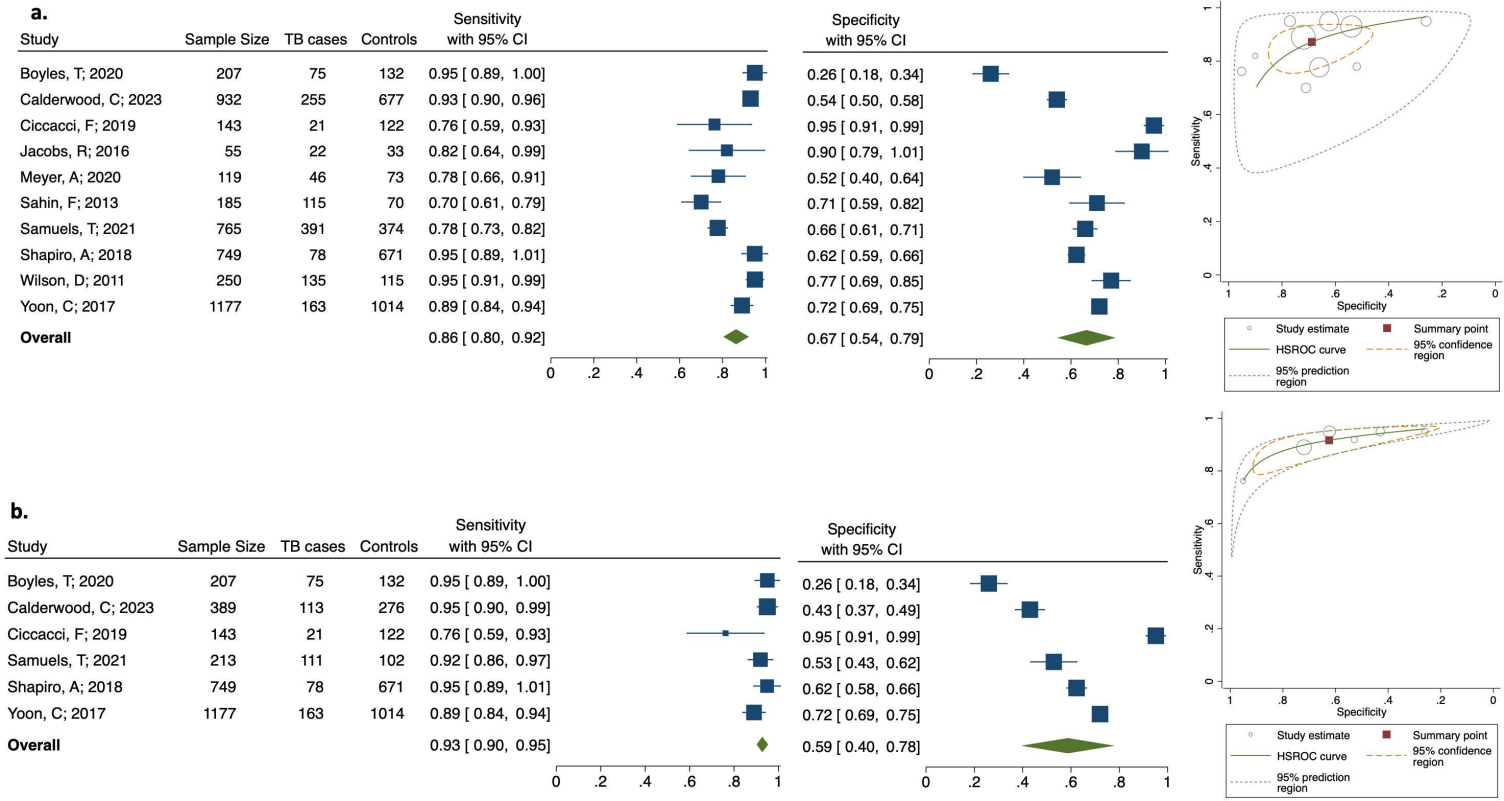
Results of CRP meta-analysis at 10 mg/L cut-off. (a) Meta-analysis of CRP from adult pulmonary TB studies, forest plot, and HSROC plot. (b) Meta-analysis of CRP from adult pulmonary TB studies of PLHIV, forest plot, and HSROC plot. Marker size is proportional to sample size. Control group for all studies was participants with ORD, except Ciccacci and Yoon were HIV-positive pre-ART.

The results of CRP at a cut-off of 10mg/L were meta-analyzed from 10 studies (17, 18, 20, 31, 33, 48–52) (Fig. 5a). The pooled sensitivity was 86% (95% CI: 80–95) and the pooled specificity with 67% (95% CI: 54–79) (Fig. 5a). Visually assessing the forest plots and HSROC curves indicates that the heterogeneity in CRP results was moderate for sensitivity but high for specificity. The results of CRP in only PLHIV at a cut-off of 10 mg/L were meta-analyzed from six studies (18, 20, 48, 49, 51, 52) (Fig. 5b). The pooled sensitivity was 93% (95% CI: 90–95), and the pooled specificity was 59% (95% CI: 40–78). One study by Ciccacci et al. from Mozambique that tested frozen plasma samples on ELISA was an outlier, reporting lower sensitivity but higher specificity than the others (49). This study enrolled PLHIV being screened for TB before starting ART.

## DISCUSSION

This is the first systematic review to comprehensively look at host blood protein biomarkers for TB disease that could translate into POC assays. A number of individual biomarkers and biomarker signatures met the TPP performance criteria for a screening test and are promising. However, most markers were evaluated by only one study each in small study populations that were likely selected in a biased manner and require further validation. For biomarkers that were evaluated in multiple studies, the heterogeneity in study designs and biomarker cut-offs make it difficult to draw broad conclusions.

When considering the risk of bias, the most promising individual host proteins for adult PTB are CRP (31), IP-10 (32), NCAM-1 (33), and SAA (28). These markers not only met TPP criteria but were evaluated in clinically relevant populations of patients with presumptive TB symptoms. Many other studies were early-stage discovery studies that used participants with LTBI or healthy controls, which likely overestimated biomarker performance (53).

Also, many studies excluded PLHIV, limiting the applicability of findings to this group. CD14 was the best performing marker out of the limited number tested in PLHIV (30), and also performed well in HIV-negative participants (22). In studies evaluating participants with ORD that enrolled a population of mixed HIV status, CRP (33) and IP-10 (32) had high accuracy.

CRP was the most-studied marker, and our results support the findings of previous reviews that have shown it not to perform well enough as a single marker (8, 9, 54). While WHO recommends a CRP cut-off of 5 mg/L for PLHIV (55) our review did not have a sufficient number of results at this cut-off to perform a meta-analysis.

CRP is a non-specific inflammatory marker, which is reflected by the low specificity in our meta-analysis (67% for adult PTB, 59% in PLHIV), which does not meet TPP requirements. Two recent studies evaluating CRP for community screening showed lower accuracy than facility-based studies, with an AUC ranging from 0.59 to 0.75 (19, 56), highlighting the fact that setting, prevalence, and patient population further impact test performance. Nevertheless, CRP is the only screening test considered by the WHO to date which has a lateral-flow format meeting the operational characteristics defined in the TPP.

CRP was also the only biomarker with enough previous research to establish pre-defined cut-offs. Many other biomarkers have not been evaluated before, and studies were done for the purpose of discovery and the cut-off thus defined post-hoc to optimize performance. As the body of evidence for biomarkers is growing, it will be important to develop consensus cut-offs and conduct validation studies with fixed, pre-defined cut-offs. This will enable results to be compared across studies and perform meta-analyses in the future.

As the performance of individual markers often does not meet TPP requirements in clinically relevant populations, combinations of markers will likely be necessary. One of the best performing combinations in patients with symptoms of presumptive TB was the 6-marker signature by De Groote et al. (34). Building off of this study, a recent analysis using machine-learning methods identified a 3-marker signature of I-309, SYWC, and kallistatin with an AUC of 0.90 (39). The other signatures that met TPP in studies with a low risk of bias for patient selection were a 4-marker combination with an AUC of 0.93 (22) and a 9-marker combination with an AUC of 0.84 (35). These results may indicate a plateau in performance around AUC 0.90, wherein adding additional biomarkers to a signature may not increase the diagnostic accuracy any further.

Ideally, the same markers would perform well across multiple populations, such as PTB and EPTB, and between adults and children. However, our review did not identify many overlapping markers between patient groups. Possible reasons for this could be differences in the studies themselves (e.g., different reference standards) (57) but also in the host response to TB disease, particularly in children (58).

The best sample for blood-based POC tests would be fresh capillary (finger-prick) blood because it is easy to collect and does not require processing. However, most studies used frozen plasma or serum samples. While this is the most feasible way to do discovery and early validation studies, there are differences when a capillary sample is used (59, 60) and further validation studies on the relevant clinical sample will be needed.

The most commonly used platforms for protein measurement in this review were ELISA and Luminex, which differ in their limits of detection and quantification. While data generated on ELISA can be conceivably translated into a lateral-flow assay, the translation from a multiplexing platform such as Luminex is more difficult as it has superior sensitivity and a broader dynamic range than ELISA (61). Studies that used a POC CRP assay reported lower sensitivity on average than studies using other methods such as ELISA and Luminex, although heterogeneity in study designs makes it difficult to compare results between platforms directly. Of the biomarkers that met TPP in adult PTB, studies used a POC test (18), turbidimetry (31), and nephelometry (28) indicating that less sensitive assays can still achieve high diagnostic accuracy.

Multiplex LFAs are a possible solution to achieve the TPP objective for a screening test; this would enable testing a combination of biomarkers on low-cost POC platform. Multiplex LFAs are not yet common and typically restricted to three markers with similar concentration ranges and LFAs have limited ability for quantification (62). However, advancements have been made in developing LFAs for cardiac biomarkers, including an up-converting phosphor technology-based lateral-flow assay for diagnosis of acute heart failure as a POC test (63, 64). There is a study currently evaluating a 3-host protein marker lateral-flow assay as a screening test for TB (65).

### Strengths

Our review conducted a comprehensive literature search and included studies conducted in 17 countries. Study quality was assessed using the standard QUADAS-2 tool, and the inclusion criteria requiring an MRS for adult PTB reduced the risk of bias for misclassifying the TB group.

### Limitations

Most biomarkers were investigated in one study each, limiting the meta-analysis to CRP. For studies that reported results at multiple cut-off values, the a-priori decision to extract the results with higher sensitivity resulted in a bias toward including the results with higher sensitivity and lower specificity. Despite our comprehensive literature search, there is a possibility that some papers could have been missed.

### Recommendations for future research

As there are few individual host proteins that meet the TPP requirements in high-quality studies, further work should be done validating these markers and new combinations of markers in populations that reflect the intended use case. Validation studies should be conducted using fresh blood samples on POC assays, and standardized, ideally pre-defined cut-off values should be used whenever possible. There is especially a need for more biomarker studies in children and PLHIV, both with PTB and EPTB disease.

### Conclusion

The large number of host blood protein biomarkers studied indicates a strong interest in this area of research, and some biomarkers have promising performance under controlled research conditions. However, further advancements are needed in testing on POC platforms as well as prospective validation studies using clinically relevant populations and sample types.

## SUPPLEMENTAL MATERIAL

Supplemental material jcm.00786-24-s0001.docx Tables S1 to S11; Fig. S1.

ASM does not own the copyrights to Supplemental Material that may be linked to, or accessed through, an article. The authors have granted ASM a non-exclusive, world-wide license to publish the Supplemental Material files. Please contact the corresponding author directly for reuse.
