# Supplementary material for: Host blood protein biomarkers to screen for tuberculosis disease: a systematic review and meta-analysis
Source: J Clin Microbiol. 2024 Oct 24;62(11):e00786-24. doi: 10.1128/jcm.00786-24 (PMC11559064; doi:10.1128/jcm.00786-24)
Supplement: Supplemental material — Tables S1 to S11; Fig. S1. [file jcm.00786-24-s0001.docx]

**Host blood protein biomarkers to screen for Tuberculosis disease: a systematic review and meta-analysis**

**Supplemental Materials**

**Table S1: Search strategy**

|  |  |  |
| --- | --- | --- |
| #1 | P: TB | "Mycobacterium tuberculosis"[MeSH] OR "Tuberculosis"[MeSH] OR Tuberculo*[tiab] OR TB[tiab] |
| #2 | I: protein,  cytokine | "Cytokines/blood"[MeSH] OR "Blood Proteins"[MeSH] OR "Biomarkers/blood"[MeSH] OR proteom*[tiab] OR cytokin*[tiab] OR CRP[tiab] OR "C-reactive protein"[tiab] OR “IP-10”[tiab] |
| #3 | I: blood | blood[tw] OR plasma[tw] OR serum[tw] |
| #4 | O: diagnosis | "diagnosis"[MeSH] OR "Sensitivity and Specificity"[MeSH] OR diagnos*[tiab] OR detect*[tiab] OR discriminat*[tiab] |
| #5 | Date | "2010/01/01"[PDat "2023/10/05"[PDat] |
| #6 |  | (#1) AND (#2) AND (#3) AND (#4) AND (#5) |

**Table S2: Modified QUADAS-2 template**

| **Domain**  **Signaling Question** | | **Accepted values and Answers** |
| --- | --- | --- |
| **Domain 1: Patient selection** | | |
| 1.) Was a case-control design avoided? | | Cohort → yes  Cross-sectional → yes  Case-control → no  NR → unclear |
| 2.) Was a consecutive or random sample of patients enrolled? | | Random → yes  Consecutive → yes  Convenience → no  NR → unclear |
| 3.) Did the study avoid inappropriate exclusions?  *Example exclude co-infections, cancer, pregnancy other ‘difficult to diagnose’ patients.* | | Yes → yes  No → no  NR → unclear |
| Scoring | Yes on ≥2 questions → Low  No on 1 question irrespective of others→ Intermediate  No on ≥2 questions → High  Unclear on ≥2 questions → Unclear | |
| **Applicability** | Did the included patients match the review question?  *Did the entire study population (cases and comparators) have symptoms of presumed TB (pulmonary and/or extra-pulmonary)?* | Low  High  Unclear |
| **Domain 2: Index Test** | | |
| 1.) Was the index test interpreted in a blinded manner, without knowledge of the reference standard results? | | Yes → yes  No → no  NR → unclear |
| 2.) If a cut-off was used, was it pre-specified or validated with another cohort? | | Yes → yes  No → no  NR → unclear |
|  |  |  |
| Scoring | Yes on both questions 🡪 Low  Yes on 2. Irrespective of answer to 1. → Low  No on 2. And Yes on 1. 🡪 Intermediate  No on both questions → High  1 Yes + 1 Unclear *or* Unclear on both questions → Unclear | |
| **Applicability** | Are there concerns that the index test, its conduct, or its interpretation differ from the review question?  *Was a POC test used?* | Low  High  Unclear |
| **Domain 3: Reference Standard** | | |
| 1.) Is the reference standard likely to correctly classify the target condition? | | Culture/Xpert → yes  In house-PCR → unclear |
|  |  |  |
| 2.) Were the reference standard results interpreted without knowledge of the results of the index test? | | Yes → yes  No → no  NR → unclear |
| Scoring | Yes on both questions → Low  Yes on 1. And No on 2. 🡪 Intermediate  Unclear on 1. And no on 2. 🡪 High  1 Yes + 1 Unclear *or* Unclear on both questions → Unclear | |
| **Applicability** | Are there concerns that the target condition as defined by the reference standard does not match the review question? | Low  High  Unclear |
| **Domain 4: Flow and Timing** | | |
| 1. Was there an appropriate interval between index tests and reference standard?   *Was the blood for the index test drawn before or within the two days of the start of TB treatment?* | | Yes → yes  No → no  NR → unclear |
|  |  |  |
| 2.) Did entire study population (cases and comparator groups) have the same reference standard testing? | | Yes → yes  No → no  NR → unclear |
| 3.) Were all patients included in the analysis? | | Yes → yes  No → no  NR → unclear |
| Scoring | Yes on ≥2 questions → Low  No on 1 question irrespective of others→ Intermediate  No on ≥2 questions → High  Unclear on ≥2 questions → Unclear | |

**Supplementary Methods: Hierarchy of control groups extracted**

Use case as a screening test, best control is group is patients presenting with symptoms but diagnosed with other respiratory diseases (ORD). We preferentially extracted data for the negative control group with the highest clinical relevance in the following order:

1. Patients suspected of TB but diagnosed with ORD (or other EP disease)
2. Patients enrolled with known ORD
3. Patients enrolled with other diseases, including cancer for EPTB studies
4. Patients with latent TB infection (LTBI)
5. Asymptomatic individuals with known TB contact (e.g. household contacts)
6. Health individuals from endemic countries (e.g. blood donors)
7. Healthy individuals from non-endemic countries (e.g. Norway healthy controls)

**Table S3: Biomarker abbreviations**

| **Abbreviation** | **Name** |
| --- | --- |
| α1AGP1 | α1-acid glycoprotein |
| α-2-M | alpha-2-macroglobulin |
| A1At | Alpha-1 antitrypsin (α1AT); Serpin Peptidase Inhibitor Clade A Member 1 (SERPINA1) |
| Aβ40 | Amyloid β 40 |
| Aβ42 | Amyloid β 42 |
| ADA | Adenosine deaminase |
| ADAMTS-13 | a disintegrin and metalloproteinase with a thrombospondin type 1 motif, member 13 |
| Apo-AI | Apolipoprotein A1 |
| Apo-CI | Apolipoprotein C1 |
| Apo-CII | Apolipoprotein C-II |
| Apo-CIII | Apolipoprotein C3 |
| Apo-E | Apolipoprotein E |
| - | Apotransferrin |
| AT-III | Antithrombin III |
| BDNF | Brain-derived neurotrophic factor |
| bFGF | Basic fibroblast growth factor |
| C1q | Complement component 1q |
| C2 | Complement component 2 |
| C3 | Complement component 3 |
| C4 | Complement component 4 |
| C4b | Complement component 4b |
| C5 | Complement component 5 |
| C5a | Complement component 5a |
| C9 | Complement component 9 |
| C1-INH | C1-inhibitor |
| CA-125 | Cancer antigen 125 |
| CALCOCO2 | Calcium-binding and coiled-coil domain-containing protein 2 |
| CAMP | Cathelicidin antimicrobial peptide ; LL-37 |
| CCL14 | C-C motif ligand 14; HCC-1 |
| CD14 | Cluster of differentiation 14 |
| CD-163 | Cluster of differentiation 163 |
| CD40L | CD40 ligand; Cluster of Differentiation 154 (CD154) |
| CD52 | Cluster of differentiation 52; CAMPATH-1 antigen |
| CEA | Carcinoembryonic antigen |
| CFD | Complement factor D; Adipsin |
| CFH | Complement factor H |
| CFHR2 | Complement factor H-related protein 2 |
| CFHR3 | Complement factor H-related protein 3 |
| CFHR5 | Complement factor H-related protein 5 |
| CFI | Complement factor I |
| CK-MB | Creatine kinase myocardial band |
| CLEC3B | C-Type Lectin Domain Family 3 Member B; Tetranectin |
| CRP | C-reactive protein |
| CXCL1 | chemokine ligand 1; Growth-related oncogene (GRO1); Growth-regulated alpha protein |
| CXCL2 | Chemokine ligand 2; growth-regulated oncogene (GRO2); growth-regulated protein beta |
| CXCL11 | C-X-C motif chemokine ligand 11; I-TAC |
| CXCL13 | Chemokine ligand 13; B lymphocyte chemoattractant (BLC); B cell-attracting chemokine 1 (BCA-1) |
| - | D-dimer |
| ECM1 | Extracellular matrix protein 1 |
| Eotaxin | Eosinophil chemotactic protein; C-C motif chemokine 11 (CCL11) |
| FAS | Fas cell surface death receptor; tumor necrosis factor receptor superfamily member 6 (TNFRSF6) |
| - | Ferretin |
| - | Fibrinogen |
| Gal-9 | Galectin-9 |
| G-CSF | Granulocyte colony-stimulating factor; colony-stimulating factor 3 (CSF 3) |
| GBP1 | Interferon-induced guanylate-binding protein 1 |
| GDF-15 | Growth/differentiation factor 15 |
| GDNF | Glial cell line-derived neurotrophic factor |
| GM-CSF | Granulocyte-macrophage colony-stimulating factor; colony-stimulating factor 2 (CSF2) |
| HEPC | Hepcidin |
| HO-1 | Heme oxygenase 1 |
| HP | Haptoglobin |
| HPX | Hemopexin |
| I-309 | Small inducible cytokine A1; Chemokine ligand 1 (CCL1) |
| ICAM-1 | Intercellular Adhesion Molecule 1; Cluster of Differentiation 54 (CD54) |
| IFIT3 | Interferon-induced protein with tetratricopeptide repeats 3 |
| IFITM3 | Interferon-induced transmembrane protein 3 |
| IFN-α-2 | Interferon alpha-2 |
| IFN-γ | Interferon-γ |
| IGFBP-3 | Insulin-like growth factor-binding protein 3 |
| IL-1α | Interleukin-1 alpha |
| IL-1β | Interleukin-1 beta |
| IL-1Ra | Interleukin-1 receptor antagonist |
| IL-2 | Interleukin-2 |
| IL-2R | Interleukin-2 receptor |
| IL-4 | Interleukin-4 |
| IL-5 | Interleukin-5 |
| IL-6 | Interleukin-6 |
| IL-7 | Interleukin-7 |
| IL-8 | Interleukin-8; CXCL8 |
| IL-9 | Interleukin-9 |
| IL-10 | Interleukin-10 |
| IL-12 | Interleukin-12 |
| IL-12p40 | Interleukin-12 subunit beta; Interleukin-12p40 |
| IL-12p70 | Interleukin-12 subunit p70 |
| IL-13 | Interleukin-13 |
| IL-17A | Interleukin-17A |
| IL-18 | Interleukin-18 |
| IL-21 | Interleukin-21 |
| IL-33 | Interleukin-33 |
| IP-10 | Interferon gamma-induced protein; CXCL10 |
| ITIH2 | Inter-alpha-trypsin inhibitor heavy chain H2 |
| - | Kallistatin |
| KLKB1 | Kallikrein B1 |
| KNG1 | Kininogen-1 |
| LBP | Lipopolysaccharide-binding protein |
| - |  |
| LL-37 | Cathelicidin LL-37 |
| LTA | Lymphotoxin-alpha; Tumor necrosis factor β (TNF-β) |
| MBL | Mannose binding lectin |
| MCP-1 | Monocyte Chemoattractant Protein-1; Chemokine C-C Motif Ligand 2 (CCL-2) |
| MDC | Macrophage-derived chemokine; C-C Motif Chemokine 22 (CCL-22) |
| MICA | MHC class I polypeptide-related sequence A |
| MIG | Monokine induced by gamma interferon; Chemokine C-X-C Motif Ligand 9 (CXCL-9) |
| MIP-1α | Macrophage Inflammatory Protein 1 Alpha; Chemokine (C-C Motif) Ligand 3 (CCL3) |
| MIP-1β | Macrophage inflammatory protein 1-Beta; Chemokine (C-C Motif) Ligand 4 (CCL4) |
| MIP-4 | Macrophage inflammatory protein 4; Chemokine (C-C Motif) Ligand 18 (CCL18) |
| MMP-1 | Matrix metallopeptidase 1 |
| MMP-2 | Matrix metallopeptidase 2 |
| MMP-3 | Matrix metallopeptidase 3 |
| MMP-7 | Matrix metallopeptidase 7 |
| MMP-8 | Matrix metallopeptidase 8 |
| MMP-9 | Matrix metallopeptidase 9 |
| MMP-12 | Matrix metallopeptidase 12 |
| MMP-13 | Matrix metallopeptidase 13 |
| MPO | Myeloperoxidase |
| MYBPC1 | Myosin binding protein C, slow type |
| - | Myoglobin |
| NCAM-1 | Neural cell adhesion molecule 1; CD56 Antigen |
| NGAL | Neutrophil gelatinase-associated lipocalin; Lipocalin-2 |
| OPG | Osteoprotegerin; Tumor necrosis factor receptor superfamily member 11B (TR11B) |
| OPN | Ostepontin; Secreted Phosphoprotein 1 (SPP1) |
| PAI-1 | Plasminogen activator inhibitor-1; Serpin E1 |
| PCT | Procalcitonin |
| PD-L1 | Programmed death-ligand 1; cluster of differentiation 274 (CD274); B7 homolog 1 (B7-H1) |
| PDGF-AA | Platelet-derived growth factor AA |
| PDGF-AB/BB | Platelet-derived growth factor AB/BB |
| PDGF-BB | Platelet-derived growth factor BB |
| PEDF | Pigment epithelium-derived factor |
| Pla2G2A | Phospholipase A2 group IIA |
| - | P-selectin |
| RAGE | Advanced glycosylation end product-specific receptor |
| RANTES | regulated on activation, normal T cell expressed and secreted; CCL5 |
| S100A9 | S100 calcium binding protein A9 |
| S100B | S100 calcium binding protein B |
| SAA | Serum amyloid A |
| SAA-4 | Serum amyloid A-4 protein |
| SAMD9L | Sterile Alpha Motif Domain-Containing 9-Like Protein |
| SAP | Serum amyloid P component |
| SELL | Selectin L |
| SNX10 | Sorting nexin-10 |
| SOD3 | Superoxide dismutase 3 |
| SYWC | Tryptophan-tRNA ligase |
| TfR | Transferrin receptor |
| TGF-α | Transforming growth factor alpha |
| TIMP-1 | Tissue inhibitor of metalloproteinases 1 |
| TIMP-2 | Tissue inhibitor of metalloproteinases 2 |
| TIMP-3 | Tissue inhibitor of metalloproteinases 3 |
| TIMP-4 | Tissue inhibitor of metalloproteinases 4 |
| TMEM49 | Transmembrane Protein 49; Vacuole membrane protein 1 (VMP1) |
| TNF | Tumor necrosis factor-alpha |
| TPA | Tissue plasminogen activator |
| - | Transferrin; Serotransferrin |
| TTR | Transthyretin |
| VCAM-1 | Vascular cell adhesion molecule 1 |
| VEGF | Vascular endothelial growth factor (A) |

**Table S4: QUADAS-2 detailed results by paper, adult pulmonary TB**

|  | | | **Risk of Bias** | | | | | | | | **Applicability Concerns** | | | | |
| --- | --- | --- | --- | --- | --- | --- | --- | --- | --- | --- | --- | --- | --- | --- | --- |
| **Study ID** | | | **Patient selection** | | **Index Test** | **Reference standard** | | | **Flow and Timing** | | **Patient Selection** | **Index Test** | | | **Reference Standard** |
| Ahmad, R; 2019 (66) | | |  | |  |  | | |  | |  |  | | |  |
| Andrade, B; 2013 (25) | | |  | |  |  | | |  | |  |  | | |  |
| Andrade, B; 2015 (26) | | |  | |  |  | | |  | |  |  | | |  |
| Boyles, T; 2020 (20) | | |  | |  |  | | |  | |  |  | | |  |
| Calderwood, C; 2023 (62) | | |  | |  |  | | |  | |  |  | | |  |
| Chen, T; 2015 (67) | | |  | |  |  | | |  | |  |  | | |  |
| Ciccacci, F; 2019 (48) | | |  | |  |  | | |  | |  |  | | |  |
| DeGroote, M; 2017 (34) | | |  | |  |  | | |  | |  |  | | |  |
| Du, Z; 2017 (68) | | |  | |  |  | | |  | |  |  | | |  |
| Essone, P; 2022 (69) | | |  | |  |  | | |  | |  |  | | |  |
| Estevez, O; 2020 (70) | | |  | |  |  | | |  | |  |  | | |  |
| Farr, K; 2018 (71) | | |  | |  |  | | |  | |  |  | | |  |
| Fontes, C; 2023 (28) | | |  | |  |  | | |  | |  |  | | |  |
| Garlant, H; 2022 (21) | | |  | |  |  | | |  | |  |  | | |  |
| Halliday, A; 2021 (72) | | |  | |  |  | | |  | |  |  | | |  |
| Jacobs, R; 2016 (33) | | |  | |  |  | | |  | |  |  | | |  |
| Jiang, X; 2012 (73) | | |  | |  |  | | |  | |  |  | | |  |
| Kathamuthu, G; 2020 (29) | | |  | |  |  | | |  | |  |  | | |  |
| Koeppel, L; 2023 (39) | | |  | |  |  | | |  | |  |  | | |  |
| Kumar, N; 2018 (24) | | |  | |  |  | | |  | |  |  | | |  |
| Kumar, N; 2019 (74) | | |  | |  |  | | |  | |  |  | | |  |
| Lawn, S; 2013 (75) | | |  | |  |  | | |  | |  |  | | |  |
| Lee, K; 2015 (76) | | |  | |  |  | | |  | |  |  | | |  |
| Liu, Y; 2018 (30) | | |  | |  |  | | |  | |  |  | | |  |
| Lubbers, R; 2018 (36) | | |  | |  |  | | |  | |  |  | | |  |
| Lubbers, R; 2020 (77) | | |  | |  |  | | |  | |  |  | | |  |
| Luo, Y; 2020 (78) | | |  | |  |  | | |  | |  |  | | |  |
| Mateos, J; 2020 (23) | | |  | |  |  | | |  | |  |  | | |  |
| Meyer, A; 2020 (63) | | |  | |  |  | | |  | |  |  | | |  |
| Mikacic, M; 2017 (79) | | |  | |  |  | | |  | |  |  | | |  |
| Moreira, F; 2021 (80) | | |  | |  |  | | |  | |  |  | | |  |
| Morris, T; 2021 (35) | | |  | |  |  | | |  | |  |  | | |  |
| Namuganga, A; 2017 (81) | | |  | |  |  | | |  | |  |  | | |  |
| Namuganga, A; 2023 (32) | | |  | |  |  | | |  | |  |  | | |  |
| Ndiaye, M; 2022 (22) | | |  | |  |  | | |  | |  |  | | |  |
| Peruhype-Magalhaes, V; 2023 (82) | | |  | |  |  | | |  | |  |  | | |  |
| Ruperez, M; 2023 (19) | | |  | |  |  | | |  | |  |  | | |  |
| Sahin, F; 2013 (17) | | |  | |  |  | | |  | |  |  | | |  |
| Sampath, P; 2023a (38) | | |  | |  |  | | |  | |  |  | | |  |
| Sampath, P; 2023b (83) | | |  | |  |  | | |  | |  |  | | |  |
| Samuels, T; 2021 (64) | | |  | |  |  | | |  | |  |  | | |  |
| Shapiro, A; 2018 (65) | | |  | |  |  | | |  | |  |  | | |  |
| Shiratori, B; 2014 (27) | | |  | |  |  | | |  | |  |  | | |  |
| Uwimaana, E; 2021 (84) | | |  | |  |  | | |  | |  |  | | |  |
| Wilson, D; 2011 (31) | | |  | |  |  | | |  | |  |  | | |  |
| Yang, Q; 2014 (85) | | |  | |  |  | | |  | |  |  | | |  |
| Yoon, C; 2017 (18) | | |  | |  |  | | |  | |  |  | | |  |
|  | | | | | | | | | | | | | | | |
| **Legend** | Low |  | | Intermediate | | |  | High | |  | Unclear | |  |  | |

**Figure S1. Funnel plot for publication bias, all adult pulmonary TB studies**

| **Table S5. Biomarkers for adult pulmonary TB, by HIV status**  Biomarkers meeting TPP criteria are indicated in bold  AUC=area under the curve, DM=diabetes mellitus, ELISA=enzyme-linked immunosorbent assay, HC= healthy controls, LTBI=latent TB infection, MSD=Meso Scale Discovery, N=sample size, ORD=other respiratory diseases, NR=not reported | | | | | | | | | | | | | | | | | | | | | | | | | | |  |
| --- | --- | --- | --- | --- | --- | --- | --- | --- | --- | --- | --- | --- | --- | --- | --- | --- | --- | --- | --- | --- | --- | --- | --- | --- | --- | --- | --- |
| **Biomarker** | | | **Study ID** | **Country** | | | | **TB cases, N** | **Control group, N** | | **Control group, N** | | | **Sample type, condition** | **Testing method** | | | **Biomarker cut-off (unit)** | | **Sensitivity**  **(95% CI)** | | | **Specificity**  **(95% CI)** | | **AUC (95% CI)** | |  |
| **HIV-negative** | | | | | | | | | | | | | | | | | | | | | | | | | | |  |
| α1AGP1 | | | Mateos, J; 2020 (23) | Mozambique | | | | 21 | LTBI | | 15 | | | Serum, Frozen | ELISA | | | >1.810 mg/mL | | 0.81 (0.60, 0.92) | | | 0.67 (0.42, 0.85) | | 0.79 (0.64, 0.94) | |  |
| A1At | | | Halliday, A; 2021 (72) | United Kingdom | | | | 60 | ORD | | 84 | | | Serum, Frozen | Mass spectrometry | | | -0.42 NR | | 0.95 (0.86, 0.99) | | | 0.19 (0.11, 0.29) | | 0.66 (0.57, 0.75) | |  |
| Apo-AI | | | Mateos, J; 2020 (23) | Mozambique | | | | 21 | LTBI | | 15 | | | Serum, Frozen | Nephelometry | | | < 92.0 mg/dL | | 0.65 (0.43, 0.82) | | | 0.93 (0.69, 0.99) | | 0.79 (0.64, 0.94) | |  |
| Apotransferrin | | | Kumar, NP; 2018 (24) | India | | | | 44* | DM | | 44 | | | Plasma,  Frozen | ELISA | | | NR | | 0.75 (0.60, 0.87) | | | 0.43 (0.28, 0.59) | | 0.57 (NR) | |  |
| C1-INH | | | Lubbers, R; 2020 (77) | the Gambia | | | | 50 | HC | | 50 | | | Serum, Frozen | ELISA | | | NR | | 0.56 (0.41, 0.7) | | | 0.94 (0.84, 0.99) | | 0.75 (0.65, 0.85) | |  |
| C1q | | | Lubbers, R; 2018 (36) | South Africa, Italy, the Gambia, Korea, Netherlands | | | | 99 | HC | | 117 | | | Serum,  NR | ELISA | | | 300.2 μg/mL | | 0.42 (0.33, 0.53) | | | 0.91 (0.84, 0.95) | | 0.84 (0.79, 0.89) | |  |
|  |  |  | Lubbers, R; 2020 (77) | the Gambia | | | | 50 | HC | | 50 | | | Serum, Frozen | ELISA | | | NR | | 0.48 (0.34, 0.63) | | | 0.88 (0.76, 0.96) | | 0.68 (0.57, 0.79) | |  |
|  |  |  | Ndiaye, M; 2022 (22) | Madagascar | | | | 37 | LTBI | | 24 | | | Plasma, Frozen | Luminex | | | 0.566 NR | | 0.49 (0.33, 0.64) | | | 0.96 (0.80, 0.99) | | 0.71 (NR) | |  |
| CA-125 | | | Mikacic, M; 2017 (79) | Bosnia and Herzegovina | | | | 40 | ORD and previous TB | | 180 | | | Serum,  NR | Chemoluminescent immunoassay | | | > 35 IU/mL | | 0.75 (0.59, 0.87) | | | 0.68 (0.60, 0.74) | | NR | |  |
| CALCOCO2 | | | Garlant, H; 2022 (21) | India, United Kingdom | | | | 49 | LTBI | | 111 | | | Whole blood, Frozen | ELISA | | | 2792 ng/mL | | **0.92 (0.82, 0.97)** | | | **0.95 (0.89, 0.98)** | | 0.97 (0.95,0.99) | |  |
| CD14 | | | Ndiaye, M; 2022 (22) | Madagascar | | | | 37 | LTBI | | 24 | | | Plasma, Frozen | Luminex | | | 0.552 NR | | **0.97 (0.86, 0.99)** | | | **0.91 (0.74, 0.98)** | | 0.96 (NR) | |  |
| CD52 | | | Garlant, H; 2022 (21) | India, United Kingdom | | | | 49 | LTBI | | 111 | | | Serum, Frozen | ELISA | | | 1258 ng/mL | | 0.92 (0.82,0.97) | | | 0.22 (0.16,0.30) | | 0.57 (0.49,0.65) | |  |
| CEA | | | Mikacic, M; 2017 (79) | Bosnia and Herzegovina | | | | 40 | ORD and previous TB | | 180 | | | Serum,  NR | NR | | | NR | | 0.10 (0.03, 0.24) | | | 0.79 (0.72, 0.85) | | NR | |  |
| CK-MB | | | Essone, P; 2022 (69) | Gabon | | | | 26 | ORD | | 36 | | | Serum, Frozen | ELISA | | | < 515.9 pg/mL | | 0.78 (0.56, 0.93) | | | 0.74 (0.56, 0.87) | | 0.81 (0.69, 0.93) | |  |
| CLEC3B | | | Ndiaye, M; 2022 (22) | Madagascar | | | | 37 | LTBI | | 24 | | | Plasma, Frozen | Luminex | | | 0.614 NR | | 0.51 (0.36, 0.67) | | | 0.87 (0.69, 0.96) | | 0.69 (NR) | |  |
| CRP | | | Andrade, B; 2013 (25) | India | | | | 97 | LTBI | | 39 | | | Plasma,  Frozen | Luminex | | | NR | | 0.86 (0.79, 0.92) | | | 0.46 (0.30, 0.63) | | 0.71 (0.62, 0.80) | |  |
|  |  |  | Calderwood, C; 2023 (62) | South Africa | | | | 142 | ORD | | 393 | | | Serum, Frozen | Turbidimetry | | | > 10 mg/L | | 0.91 (0.85, 0.95) | | | 0.62 (0.57, 0.66) | | 0.82 (0.79, 0.86) | |  |
|  |  |  | Mateos, J; 2020 (23) | Mozambique | | | | 21 | LTBI | | 15 | | | Serum, Frozen | ELISA | | | > 70.90 μg/mL | | **0.95 (0.77, 0.99)** | | | **0.93 (0.70, 0.99)** | | 0.92 (0.80, 1.0) | |  |
|  |  |  | Meyer, A; 2020 (63) | Uganda | | | | 46 | ORD | | 73 | | | Serum, Frozen | ELISA | | | > 10 mg/L | | 0.78 (0.64, 0.89) | | | 0.52 (0.40, 0.64) | | 0.65 (0.57, 0.74) | |  |
|  |  |  | Meyer, A; 2020 (63) | Uganda | | | | 46 | ORD | | 73 | | | Serum, Frozen | ELISA | | | ≥ 1.5 mg/L | | 0.91 (0.79, 0.98) | | | 0.21 (0.12, 0.32) | | 0.65 (0.57, 0.74) | |  |
|  |  |  | Moreira, F; 2021 (80) | Brazil | | | | 100 | prisoners | | 200 | | | Serum, Frozen | Immunoassay (POC setting) | | | NR | | 0.90 (0.82, 0.95) | | | 0.28 (0.20, 0.42) | | 0.79 (0.73, 0.84) | |  |
|  |  |  | Sahin, F; 2013 (17) | Turkey | | | | 115 | ORD | | 70 | | | Serum,  NR | Turbidimetry | | | < 9.4 mg/L | | 0.70 (0.61, 0.79) | | | 0.71 (0.59, 0.82) | | 0.79 (NR) | |  |
|  |  |  | Samuels, T; 2021 (64) | Peru, South Africa, Vietnam, Cambodia, Georgia | | | | 274 | ORD | | 253 | | | Serum, Frozen | ELISA | | | 6 mg/L | | 0.79 (0.73, 0.83) | | | 0.64 (0.58, 0.70) | | 0.76 (0.72, 0.81) | |  |
|  |  |  | Samuels, T; 2021 (64) | Peru, South Africa, Vietnam, Cambodia, Georgia | | | | 274 | ORD | | 253 | | | Serum, Frozen | ELISA | | | ≥ 10 mg/L | | 0.73 (0.67, 0.78) | | | 0.71 (0.65, 0.76) | | 0.76 (0.72, 0.81) | |  |
| CXCL1 | | | Kumar, NP; 2019 (74) | India | | | | 88 | LTBI | | 44 | | | Plasma, Frozen | Luminex | | | NR | | 0.69 (0.59, 0.79) | | | 0.53 (0.38, 0.66) | | 0.68 (NR) | |  |
| CXCL11 | | | Kumar, NP; 2019 (74) | India | | | | 88 | LTBI | | 44 | | | Plasma, Frozen | Luminex | | | NR | | 0.45 (0.35, 0.56) | | | 0.93 (0.82, 0.98) | | 0.71 (NR) | |  |
|  |  |  | Lee, K; 2015 (76) | South Korea | | | | 165 | ORD | | 389 | | | Plasma, Frozen | ELISA | | | 60.5 pg/mL | | 0.73 (0.65, 0.79) | | | 0.85 (0.81, 0.88) | | 0.86 (0.83, 0.89) | |  |
| CXCL13 | | | Estevez, O; 2020 (70) | Spain | | | | 28 | LTBI | | 27 | | | Serum, Frozen | Luminex | | | 28.03 pg/mL | | 0.72 (0.51, 0.88) | | | 0.80 (0.59, 0.93) | | 0.78 (NR) | |  |
| CXCL2 | | | Kumar, NP; 2019 (74) | India | | | | 88 | LTBI | | 44 | | | Plasma, Frozen | Luminex | | | NR | | 0.78 (0.69, 0.86) | | | 0.59 (0.44, 0.72) | | 0.66 (NR) | |  |
| ECM1 | | | Ndiaye, M; 2022 (22) | Madagascar | | | | 37 | LTBI | | 24 | | | Plasma, Frozen | Luminex | | | 0.628 NR | | 0.32 (0.20, 0.49) | | | 0.91 (0.74, 0.98) | | 0.58 (NR) | |  |
| Eotaxin | | | Peruhype-Magalhaes, P; 2023 (82) | Brazil | | | | 84 | HC | | 79 | | | Serum, NR | Luminex | | | 10.3 pg/mL | | 0.63 (0.51, 0.74) | | | 0.47 (0.36, 0.58) | | 0.50 (0.42, 0.60) | |  |
| Ferretin | | | Kumar, NP; 2018 (24) | India | | | | 44* | DM | | 44 | | | Plasma,  Frozen | ELISA | | | NR | | **0.93 (0.81, 0.99)** | | | **0.75 (0.60, 0.87)** | | 0.92 (NR) | |  |
| Gal-9 | | | Shiratori, B; 2014 (27) | Phillipines | | | | 37 | HC | | 30 | | | Plasma, Frozen | ELISA | | | > 258 pg/mL | | 0.76 (0.59, 0.88) | | | 0.80 (0.61, 0.92) | | 0.77 (NR) | |  |
| GBP1 | | | Garlant, H; 2022 (21) | India, United Kingdom | | | | 49 | LTBI | | 111 | | | Serum, Frozen | ELISA | | | 2728 ng/mL | | **0.92 (0.82, 0.97)** | | | **0.82 (0.75, 0.88)** | | 0.95 (0.92, 0.98) | |  |
| G-CSF | | | Peruhype-Magalhaes, P; 2023 (82) | Brazil | | | | 84 | HC | | 79 | | | Serum, NR | Luminex | | | 2.7 pg/mL | | 0.72 (0.61,0.82) | | | 0.71 (0.57, 0.82) | | 0.75 (0.66, 0.82) | |  |
|  |  |  | Yang, Q; 2014 (85) | China | | | | 20 | LTBI | | 17 | | | Plasma, Frozen | Luminex | | | 34.83 pg/mL | | 0.75 (0.51, 0.91) | | | 0.88 (0.64, 0.99) | | 0.83 (0.68, 0.97) | |  |
| HEPC | | | Essone, P; 2022 (69) | Gabon | | | | 26 | ORD | | 36 | | | Serum, Frozen | ELISA | | | < 130.6 ng/mL | | 0.87 (0.66, 0.97) | | | 0.64 (0.46, 0.80) | | 0.79 (0.67, 0.91) | |  |
|  |  |  | Kumar, NP; 2018 (24) | India | | | | 44* | DM | | 44 | | | Plasma,  Frozen | ELISA | | | NR | | 0.89 (0.75, 0.96) | | | 0.55 (0.39, 0.70) | | 0.70 (NR) | |  |
| HO-1 | | | Andrade, B; 2013 (25) | India | | | | 97 | LTBI | | 39 | | | Plasma,  Frozen | ELISA | | | NR | | **0.92 (0.85, 0.96)** | | | **0.95 (0.83, 0.99)** | | 0.95 (0.91, 0.98) | |  |
|  |  |  | Andrade, B; 2015 (26) | Brazil | | | | 63 | LTBI | | 15 | | | Plasma, Frozen | ELISA | | | > 2.397 ng/mL | | **0.92 (0.82, 0.97)** | | | **0.93 (0.68, 0.99)** | | 0.95 (0.91, 1.0) | |  |
|  |  |  | Andrade, B; 2015 (26) | India | | | | 97 | LTBI | | 39 | | | Plasma, Frozen | ELISA | | | > 1.65 ng/mL | | **0.92 (0.84, 0.96)** | | | **0.93 (0.66, 0.99)** | | 0.94 (0.90, 0.98) | |  |
| HP | | | Mateos, J; 2020 (23) | Mozambique | | | | 21 | LTBI | | 15 | | | Serum, Frozen | ELISA | | | > 15.42 μg/mL | | 0.62 (0.41, 0.79) | | | 0.80 (0.55, 0.93) | | 0.67 (0.49, 0.85) | |  |
| HPX | | | Kumar, NP; 2018 (24) | India | | | | 44* | DM | | 44 | | | Plasma,  Frozen | ELISA | | | NR | | 0.59 (0.43, 0.74) | | | 0.80 (0.65, 0.90) | | 0.59 (NR) | |  |
| I-309 | | | Kumar, NP; 2019 (74) | India | | | | 88 | LTBI | | 44 | | | Plasma, Frozen | Luminex | | | NR | | 0.58 (0.48, 0.68) | | | 0.89 (0.76, 0.95) | | 0.74 (NR) | |  |
| IFIT3 | | | Garlant, H; 2022 (21) | India, United Kingdom | | | | 49 | LTBI | | 111 | | | Whole blood, Frozen | ELISA | | | 8.749 ng/mL | | **0.92 (0.83, 0.97)** | | | **0.86 (0.80, 0.81)** | | 0.93 (0.89, 0.96) | |  |
| IFITM3 | | | Garlant, H; 2022 (21) | India, United Kingdom | | | | 49 | LTBI | | 111 | | | Whole blood, Frozen | ELISA | | | 3105 ng/mL | | **0.92 (0.83, 0.97)** | | | **1.0 (0.97, 1.0)** | | 0.98 (0.95, 1.0) | |  |
| IFN-γ | | | Lee, K; 2015 (76) | South Korea | | | | 165 | ORD | | 389 | | | Plasma, Frozen | ELISA | | | 15.2 pg/mL | | 0.39 (0.31, 0.47) | | | 0.69 (0.64, 0.73) | | 0.48 (0.42, 0.54) | |  |
|  |  |  | Peruhype-Magalhaes, P; 2023 (82) | Brazil | | | | 84 | HC | | 79 | | | Serum, NR | Luminex | | | 13.6 pg/mL | | 0.77 (0.66, 0.85) | | | 0.47 (0.35, 0.59) | | 0.62 (0.54, 0.69) | |  |
| IGFBP-3 | | | Ndiaye, M; 2022 (22) | Madagascar | | | | 37 | LTBI | | 24 | | | Plasma, Frozen | Luminex | | | 0.532 NR | | 0.73 (0.57, 0.85) | | | 0.87 (0.69, 0.96) | | 0.84 (NR) | |  |
| IL-1β | | | Peruhype-Magalhaes, P; 2023 (82) | Brazil | | | | 84 | HC | | 79 | | | Serum, NR | Luminex | | | 1.0 pg/mL | | 0.44 (0.33, 0.55) | | | 0.80 (0.70, 0.89) | | 0.64 (0.56, 0.71) | |  |
| IL-1Ra | | | Peruhype-Magalhaes, P; 2023 (82) | Brazil | | | | 84 | HC | | 79 | | | Serum, NR | Luminex | | | 11.4 pg/mL | | 0.50 (0.38, 0.62) | | | 0.74 (0.62, 0.84) | | 0.60 (0.51, 0.68) | |  |
| IL-4 | | | Peruhype-Magalhaes, P; 2023 (82) | Brazil | | | | 84 | HC | | 79 | | | Serum, NR | Luminex | | | 0.3 pg/mL | | 0.45 (0.34, 0.57) | | | 0.78 (0.67, 0.87) | | 0.62 (0.54, 0.69) | |  |
| IL-6 | | | Estevez, O; 2020 (70) | Spain | | | | 28 | LTBI | | 27 | | | Serum, Frozen | Luminex | | | > 0.0292 pg/mL | | 1.0 (0.86, 1.0) | | | 0.45 (0.26, 0.67) | | 0.62 (NR) | |  |
|  |  |  | Peruhype-Magalhaes, P; 2023 (82) | Brazil | | | | 84 | HC | | 79 | | | Serum, NR | Luminex | | | 1.0 pg/mL | | 0.87 (0.78, 0.93) | | | 0.81 (0.71, 0.89) | | 0.90 (0.84, 0.94) | |  |
| IL-7 | | | Estevez, O; 2020 (70) | Spain | | | | 28 | LTBI | | 27 | | | Serum, Frozen | Luminex | | | 5.391 pg/mL | | 0.76 (0.55, 0.91) | | | 0.67 (0.41, 0.87) | | 0.73 (NR) | |  |
|  |  |  | Peruhype-Magalhaes, P; 2023 (82) | Brazil | | | | 84 | HC | | 79 | | | Serum, NR | Luminex | | | 2.2 pg/mL | | 0.64 (0.50, 0.75) | | | 0.69 (0.54, 0.81) | | 0.63 (0.53, 0.71) | |  |
| IL-8 | | | Peruhype-Magalhaes, P; 2023 (82) | Brazil | | | | 84 | HC | | 79 | | | Serum, NR | Luminex | | | 5.0 pg/mL | | 0.69 (0.58, 0.79) | | | 0.71 (0.59, 0.80) | | 0.72 (0.64, 0.80) | |  |
|  |  |  | Shiratori, B; 2014 (27) | Phillipines | | | | 37 | HC | | 30 | | | Plasma, Frozen | ELISA | | | > 3.5 pg/mL | | 0.76 (0.59, 0.88) | | | 0.77 (0.58, 0.90) | | 0.75 (NR) | |  |
| IL-9 | | | Peruhype-Magalhaes, P; 2023 (82) | Brazil | | | | 84 | HC | | 79 | | | Serum, NR | Luminex | | | 1.1 pg/mL | | 0.78 (0.60, 0.91) | | | 0.46 (0.28, 0.64) | | 0.54 (0.40, 0.66) | |  |
| IL-10 | | | Peruhype-Magalhaes, P; 2023 (82) | Brazil | | | | 84 | HC | | 79 | | | Serum, NR | Luminex | | | 0.9 pg/mL | | 0.64 (0.53, 0.74) | | | 0.54 (0.42, 0.66) | | 0.62 (0.54, 0.69) | |  |
| IL-12 | | | Peruhype-Magalhaes, P; 2023 (82) | Brazil | | | | 84 | HC | | 79 | | | Serum, NR | Luminex | | | 0.2 pg/mL | | 0.93 (0.78, 0.99) | | | 0.20 (0.05, 0.48) | | 0.54 (0.38, 0.69) | |  |
| IL-13 | | | Peruhype-Magalhaes, P; 2023 (82) | Brazil | | | | 84 | HC | | 79 | | | Serum, NR | Luminex | | | 1.4 pg/mL | | 0.16 (0.08, 0.26) | | | 0.97 (0.89, 0.99) | | 0.52 (0.43, 0.60) | |  |
| IL-17A | | | Peruhype-Magalhaes, P; 2023 (82) | Brazil | | | | 84 | HC | | 79 | | | Serum, NR | Luminex | | | 0.8 pg/mL | | 0.65 (0.43, 0.82) | | | 0.67 (0.35, 0.88) | | 0.60 (0.38, 0.82) | |  |
| IP-10 | | | Estevez, O; 2020 (70) | Spain | | | | 28 | LTBI | | 27 | | | Serum, Frozen | Luminex | | | 450.9 pg/mL | | 0.64 (0.43, 0.82) | | | 0.92 (0.74, 0.99) | | 0.78 (NR) | |  |
|  |  |  | Kumar, NP; 2019 (74) | India | | | | 88 | LTBI | | 44 | | | Plasma, Frozen | Luminex | | | NR | | 0.81 (0.71, 0.88) | | | 0.50 (0.36, 0.64) | | 0.74 (NR) | |  |
|  |  |  | Lee, K; 2015 (76) | South Korea | | | | 165 | ORD | | 389 | | | Plasma, Frozen | ELISA | | | 68.5 pg/mL | | 0.74 (0.67, 0.81) | | | 0.65 (0.60, 0.70) | | 0.74 (0.70, 0.78) | |  |
|  |  |  | Ndiaye, M; 2022 (22) | Madagascar | | | | 37 | LTBI | | 24 | | | Plasma, Frozen | Luminex | | | 0.594 NR | | 0.54 (0.38,0.69) | | | 1.0 (0.86,1.0) | | 0.76 (NR) | |  |
|  |  |  | Peruhype-Magalhaes, P; 2023 (82) | Brazil | | | | 84 | HC | | 79 | | | Serum, NR | Luminex | | | 285.1 pg/mL | | 0.73 (0.62, 0.82) | | | 0.89 (0.80, 0.95) | | 0.86 (0.80, 0.90) | |  |
|  |  |  | Shiratori, B; 2014 (27) | Phillipines | | | | 37 | HC | | 30 | | | Plasma, Frozen | ELISA | | | >342 pg/mL | | **0.95 (0.82, 0.99)** | | | **0.93 (0.78, 0.99)** | | 0.99 (NR) | |  |
|  |  |  | Yang, Q; 2014 (85) | China | | | | 20 | LTBI | | 17 | | | Plasma, Frozen | ELISA | | | 1,008 pg/mL | | 0.88 (0.47, 0.99) | | | 0.92 (0.62, 0.99) | | 0.92 (0.78, 1.05) | |  |
| KLKB1 | | | Mateos, J; 2020 (23) | Mozambique | | | | 21 | LTBI | | 15 | | | Serum, Frozen | ELISA | | | < 338.8 μg/mL | | 0.95 (0.76, 0.99) | | | 0.47 (0.25, 0.70) | | 0.72 (0.54, 0.89) | |  |
| MCP-1 | | | Peruhype-Magalhaes, P; 2023 (82) | Brazil | | | | 84 | HC | | 79 | | | Serum, NR | Luminex | | | 3.4 pg/mL | | 0.71 (0.57, 0.82) | | | 0.48 (0.34, 0.62) | | 0.59 (0.50, 0.68) | |  |
|  |  |  | Shiratori, B; 2014 (27) | Phillipines | | | | 37 | HC | | 30 | | | Plasma, Frozen | ELISA | | | > 92 pg/mL | | 0.76 (0.59, 0.88) | | | 0.63 (0.44, 0.80) | | 0.79 (NR) | |  |
| MIG | | | Kumar, NP; 2019 (74) | India | | | | 88 | LTBI | | 44 | | | Plasma, Frozen | Luminex | | | NR | | 0.55 (0.44, 0.65) | | | 0.85 (0.71, 0.92) | | 0.74 (NR) | |  |
|  |  |  | Lee, K; 2015 (76) | South Korea | | | | 165 | ORD | | 389 | | | Plasma, Frozen | ELISA | | | 148.9 pg/mL | | 0.79 (0.72, 0.85) | | | 0.70 (0.65, 0.75) | | 0.82 (0.78, 0.85) | |  |
|  |  |  | Yang, Q; 2014 (85) | China | | | | 20 | LTBI | | 17 | | | Plasma, Frozen | ELISA | | | 1,976 pg/mL | | 0.53 (0.29, 0.76) | | | 0.94 (0.71, 0.99) | | 0.86 (0.74, 0.98) | |  |
| MIP-1α | | | Peruhype-Magalhaes, P; 2023 (82) | Brazil | | | | 84 | HC | | 79 | | | Serum, NR | Luminex | | | 1.5 pg/mL | | 0.41 (0.29, 0.54) | | | 0.84 (0.68, 0.94) | | 0.62 (0.52, 0.72) | |  |
| MIP-1β | | | Peruhype-Magalhaes, P; 2023 (82) | Brazil | | | | 84 | HC | | 79 | | | Serum, NR | Luminex | | | 7.7 pg/mL | | 0.62 (0.51, 0.72) | | | 0.57 (0.45, 0.68) | | 0.55 (0.47, 0.62) | |  |
| MMP-1 | | | Andrade, B; 2015 (26) | India | | | | 97 | LTBI | | 39 | | | Plasma, Frozen | Luminex | | | > 1.21 ng/mL | | 0.85 (0.78, 0.91) | | | 0.93 (0.66, 0.99) | | 0.96 (0.91, 1.0) | |  |
|  |  |  | Andrade, B; 2015 (26) | Brazil | | | | 63 | LTBI | | 15 | | | Plasma, Frozen | Luminex | | | > 3.511 ng/mL | | 0.78 (0.66, 0.87) | | | 0.73 (0.45, 0.92) | | 0.85 (0.76, 0.93) | |  |
|  |  |  | Kathamuthu, G; 2020 (29) | India | | | | 68 | LTBI | | 44 | | | Plasma,  NR | Luminex | | | NR | | **0.99 (0.92, 0.99)** | | | **1 (0.92, 1.0)** | | 0.99 (NR) | |  |
| MMP-2 | | | Kathamuthu, G; 2020 (29) | India | | | | 68 | LTBI | | 44 | | | Plasma,  NR | Luminex | | | NR | | 0.53 (0.40, 0.65) | | | 0.50 (0.35, 0.65) | | 0.52 (NR) | |  |
| MMP-3 | | | Kathamuthu, G; 2020 (29) | India | | | | 68 | LTBI | | 44 | | | Plasma,  NR | Luminex | | | NR | | 0.57 (0.45, 0.69) | | | 0.55 (0.39, 0.70) | | 0.54 (NR) | |  |
| MMP-7 | | | Kathamuthu, G; 2020 (29) | India | | | | 68 | LTBI | | 44 | | | Plasma,  NR | Luminex | | | NR | | 0.50 (0.38, 0.62) | | | 0.50 (0.35, 0.65) | | 0.61 (NR) | |  |
| MMP-8 | | | Kathamuthu, G; 2020 (29) | India | | | | 68 | LTBI | | 44 | | | Plasma,  NR | Luminex | | | NR | | 0.66 (0.54, 0.77) | | | 0.77 (0.62, 0.89) | | 0.80 (NR) | |  |
| MMP-9 | | | Kathamuthu, G; 2020 (29) | India | | | | 68 | LTBI | | 44 | | | Plasma,  NR | Luminex | | | NR | | 0.74 (0.61, 0.84) | | | 0.75 (0.60, 0.87) | | 0.88 (NR) | |  |
| MMP-12 | | | Kathamuthu, G; 2020 (29) | India | | | | 68 | LTBI | | 44 | | | Plasma,  NR | Luminex | | | NR | | 0.63 (0.51, 0.75) | | | 0.75 (0.60, 0.87) | | 0.76 (NR) | |  |
| MMP-13 | | | Kathamuthu, G; 2020 (29) | India | | | | 68 | LTBI | | 44 | | | Plasma,  NR | Luminex | | | NR | | 0.51 (0.39, 0.64) | | | 0.82 (0.67, 0.92) | | 0.53 (NR) | |  |
| MYBPC1 | | | Essone, P; 2022 (69) | Gabon | | | | 26 | ORD | | 36 | | | Serum, Frozen | ELISA | | | > 26.98 ng/mL | | 0.7 (0.50, 0.86) | | | 0.61 (0.43, 0.77) | | 0.62 (0.48, 0.76) | |  |
| OPN | | | Shiratori, B; 2014 (27) | Phillipines | | | | 37 | HC | | 30 | | | Plasma, Frozen | ELISA | | | > 94 pg/mL | | **0.95 (0.82, 0.99)** | | | **0.93 (0.78, 0.99)** | | 0.97 (NR) | |  |
| PDGF-BB | | | Peruhype-Magalhaes, P; 2023 (82) | Brazil | | | | 84 | HC | | 79 | | | Serum, NR | Luminex | | | 249.4 pg/mL | | 0.73 (0.62, 0.83) | | | 0.52 (0.40, 0.64) | | 0.60 (0.52, 0.68) | |  |
| PD-L1 | | | Garlant, H; 2022 (21) | India, United Kingdom | | | | 49 | LTBI | | 111 | | | Whole blood, Frozen | ELISA | | | 0.5864 ng/mL | | **1.0 (0.94, 1.0)** | | | **1.0 (0.97, 1.0)** | | 1.0 (1.0, 1.0) | |  |
| PLa2G2A | | | Essone, P; 2022 (69) | Gabon | | | | 26 | ORD | | 36 | | | Serum, Frozen | ELISA | | | > 1.19 pg/mL | | 0.48 (0.29, 0.68) | | | 0.56 (0.38, 0.72) | | 0.48 (0.36, 0.65) | |  |
| RANTES | | | Peruhype-Magalhaes, P; 2023 (82) | Brazil | | | | 84 | HC | | 79 | | | Serum, NR | Luminex | | | 60.3 pg/mL | | 0.66 (0.54, 0.76) | | | 0.58 (0.47, 0.69) | | 0.62 (0.54, 0.70) | |  |
| SAA | | | Andrade, B; 2013 (25) | India | | | | 97 | LTBI | | 39 | | | Plasma,  NR | Luminex | | | NR | | **0.91 (0.85, 0.96)** | | | **0.71 (0.42, 0.92)** | | 0.89 (0.82, 0.96) | |  |
|  |  |  | Fontes, C; 2023 (28) | Brazil | | | | 51 | ORD | | 32 | | | Plasma, Frozen | Nephelometry | | | 83.85 mg/L | | **0.97 (0.87, 0.99)** | | | **0.78 (0.61, 0.89)** | | 0.90 (0.83, 0.97) | |  |
| SAMD9L | | | Garlant, H; 2022 (21) | India, United Kingdom | | | | 49 | LTBI | | 111 | | | Whole blood, Frozen | ELISA | | | 2430 ng/mL | | **0.96 (0.88, 0.99)** | | | **1.0 (0.97, 1.0)** | | 0.99 (0.99, 1.0) | |  |
| SELL | | | Ndiaye, M; 2022 (22) | Madagascar | | | | 37 | LTBI | | 24 | | | Plasma, Frozen | Luminex | | | 0.499 NR | | **0.92 (0.79, 0.97)** | | | **0.91 (0.74, 0.98)** | | 0.93 NR | |  |
| SNX10 | | | Garlant, H; 2022 (21) | India, United Kingdom | | | | 49 | LTBI | | 111 | | | Whole blood, Frozen | ELISA | | | 3177 ng/mL | | **0.92 (0.82, 0.97)** | | | **0.95 (0.91, 0.98)** | | 0.97 (0.95, 0.99) | |  |
| TfR | | | Kumar, NP; 2018 (24) | India | | | | 44* | DM | | 44 | | | Plasma,  NR | ELISA | | | NR | | 0.61 (0.46, 0.76) | | | 0.43 (0.28, 0.59) | | 0.58 (NR) | |  |
| TGF-α | | | Estevez, O; 2020 (70) | Spain | | | | 28 | LTBI | | 27 | | | Serum, Frozen | Luminex | | | 6.968 pg/mL | | 0.84 (0.64, 0.95) | | | 0.63 (0.41, 0.82) | | 0.68 (NR) | |  |
| TIMP-1 | | | Kathamuthu, G; 2020 (29) | India | | | | 68 | LTBI | | 44 | | | Plasma,  NR | Luminex | | | NR | | 0.71 (0.58, 0.81) | | | 0.84 (0.70, 0.93) | | 0.70 (NR) | |  |
| TIMP-2 | | | Kathamuthu, G; 2020 (29) | India | | | | 68 | LTBI | | 44 | | | Plasma,  NR | Luminex | | | NR | | **1.0 (0.95, 1.0)** | | | **0.95 (0.85, 0.99)** | | 0.99 (NR) | |  |
| TIMP-3 | | | Kathamuthu, G; 2020 (29) | India | | | | 68 | LTBI | | 44 | | | Plasma,  NR | Luminex | | | NR | | 0.65 (0.52, 0.76) | | | 0.95 (0.85, 0.99) | | 0.62 (NR) | |  |
| TIMP-4 | | | Kathamuthu, G; 2020 (29) | India | | | | 68 | LTBI | | 44 | | | Plasma,  NR | Luminex | | | NR | | **0.99 (0.92, 0.99)** | | | **0.95 (0.85, 0.99)** | | 0.98 (NR) | |  |
| TMEM49 | | | Garlant, H; 2022 (21) | India, United Kingdom | | | | 49 | LTBI | | 111 | | | Whole blood, Frozen | ELISA | | | 369.6 ng/mL | | 0.92 (0.82, 0.97) | | | 0.0 (0.0, 0.28) | | 0.49 (0.41, 0.57) | |  |
| TNF | | | Estevez, O; 2020 (70) | Spain | | | | 28 | LTBI | | 27 | | | Serum, Frozen | Luminex | | | 12.22 pg/mL | | 0.88 (0.69, 0.97) | | | 0.40 (0.21, 0.61) | | 0.59 (NR) | |  |
|  |  |  | Peruhype-Magalhaes, P; 2023 (82) | Brazil | | | | 84 | HC | | 79 | | | Serum, NR | Luminex | | | 9.4 pg/mL | | 0.45 (0.34, 0.56) | | | 0.71 (0.60, 0.81) | | 0.56 (0.48, 0.64) | |  |
|  |  |  | Shiratori, B; 2014 (27) | Phillipines | | | | 37 | HC | | 30 | | | Plasma, Frozen | ELISA | | | > 5.99 pg/mL | | 0.81 (0.65, 0.92) | | | 0.70 (0.51, 0.85) | | 0.80 (NR) | |  |
| Transferrin | | | Kumar, NP; 2018 (24) | India | | | | 44* | DM | | 44 | | | Plasma,  Frozen | ELISA | | | NR | | 0.89 (0.75, 0.96) | | | 0.91 (0.78, 0.98) | | 0.95 (NR) | |  |
|  |  |  | Mateos, J; 2020 (23) | Mozambique | | | | 21 | LTBI | | 15 | | | Serum, Frozen | ELISA | | | < 3928 μg/mL | | 0.77 (0.55, 0.89) | | | 0.87 (0.62, 0.98) | | 0.83 (0.69, 0.97) | |  |
| VEGF | | | Peruhype-Magalhaes, P; 2023 (82) | Brazil | | | | 84 | HC | | 79 | | | Serum, NR | Luminex | | | 9.7 pg/mL | | 0.61 (0.49, 0.73) | | | 0.76 (0.62, 0.87) | | 0.70 (0.62, 0.79) | |  |
| **HIV-positive** | | | | | | | | | | | | | | | | | | | | | | | | | | |  |
| CD14 | Liu, Y; 2018 (30) | | | | South Africa | | 39 | | HC | | | 24 | | Serum, Frozen | | | ELISA | | 1540 ng/mL | | | **0.95 (0.83, 0.99)** | | **0.96 (0.79, 0.99)** | | NR |  |
| CRP | Boyles, T; 2020 (86) | | | | South Africa | | 75 | | ORD | | | 132 | | NR, Fresh | | | Turbidimetry (POC setting) | | ≥10 mg/L | | | 0.95 (0.87, 0.98) | | 0.26 (0.19, 0.34) | | NR |  |
|  | Calderwood, C; 2023 (62) | | | | South Africa | | 113 | | ORD | | | 276 | | Serum, Frozen | | | Turbidimetry | | ≥10 mg/L | | | 0.95 (0.89, 0.98) | | 0.43 (0.37, 0.49) | | 0.77 (0.73, 0.82) |  |
|  | Ciccacci, F; 2019 (48) | | | | Mozambique | | 21 | | pre-ART screening | | | 122 | | Plasma, Frozen | | | ELISA | | > 10 mg/L | | | 0.76 (0.55, 0.89) | | 0.95 (0.90, 0.98) | | NR |  |
|  | Farr, K; 2018 (71) | | | | Uganda | | 155 | | ORD | | | 107 | | Plasma, Frozen | | | Turbidimetry | | NR | | | 0.90 (0.85, 0.95) | | 0.33 (0.30, 0.36) | | NR |  |
|  | Lawn, S; 2013 (75) | | | | South Africa | | 81 | | pre-ART screening | | | 415 | | Serum, Frozen | | | ELISA | | ≥ 5 mg/l | | | 0.90 (0.81, 0.95) | | 0.44 (0.39, 0.49) | | 0.81 (NR) |  |
|  | Ruperez, M; 2023 (19) | | | | South Africa, Zambia | | 25 | | ORD | | | 251 | | Whole blood, Fresh | | | Immunoassay  (POC setting) | | ≥10 mg/L | | | 0.40 (0.21, 0.61) | | 0.79 (0.73, 0.83) | | 0.59 (0.49, 0.69) |  |
|  | Ruperez, M; 2023 (19) | | | | South Africa, Zambia | | 25 | | ORD | | | 251 | | Whole blood, Fresh | | | Immunoassay  (POC setting) | | ≥5 mg/L | | | 0.60 (0.39, 0.79) | | 0.63 (0.57, 0.69) | | 0.61 (0.51, 0.72) |  |
|  | Samuels, T; 2021 (64) | | | | Peru, South Africa, Vietnam, Cambodia, Georgia | | 111 | | ORD | | | 102 | | Serum, Frozen | | | ELISA | | ≥ 10 mg/L | | | 0.92 (0.85, 0.96) | | 0.53 (0.43, 0.62) | | 0.83 (0.78, 0.89) |  |
|  | Shapiro, A; 2018 (65) | | | | South Africa | | 42 | | pre-ART screening | | | 383 | | Serum, Frozen | | | Turbidimetry | | > 5 mg/L | | | 0.91 (0.77, 0.97) | | 0.59 (0.53, 0.64) | | 0.80 (0.72, 0.88) |  |
|  | Shapiro, A; 2018 (65) | | | | South Africa | | 42 | | pre-ART screening | | | 383 | | Serum, Frozen | | | Turbidimetry | | > 10 mg/L | | | 0.79 (0.63, 0.90) | | 0.72 (0.68, 0.77) | | 0.80 (0.72, 0.88) |  |
|  | Yoon, C; 2017 (18) | | | | Uganda | | 163 | | pre-ART screening | | | 1014 | | Whole blood, Fresh | | | Immunoassay (POC setting) | | ≥ 8 mg/L | | | **0.90 (0.85, 0.94)** | | **0.70 (0.67, 0.72)** | | 0.81 (0.78, 0.83) |  |
|  | Yoon, C; 2017 (18) | | | | Uganda | | 163 | | pre-ART screening | | | 1014 | | Whole blood, Fresh | | | Immunoassay (POC setting) | | ≥ 10 mg/L | | | 0.89 (0.83, 0.93) | | 0.72 (0.69, 0.75) | | 0.81 (0.78, 0.83) |  |
| CXCL1 | Farr, K; 2018 (71) | | | | Uganda | | 155 | | ORD | | | 107 | | Plasma, Frozen | | | Luminex | | NR | | | 0.90 (0.85, 0.95) | | 0.01 (0.00, 0.03) | | NR |  |
| HO-1 | Uwimaana, E; 2021 (84) | | | | Uganda | | 70 | | LTBI | | | 70 | | Plasma, Frozen | | | ELISA | | > 8.95 ng/mL | | | 0.59 (0.47, 0.69) | | 0.67 (0.56, 0.77) | | 0.57 (0.47, 0.66) |  |
| IFN-γ | Farr, K; 2018 (71) | | | | Uganda | | 155 | | ORD | | | 107 | | Plasma, Frozen | | | Luminex | | NR | | | 0.90 (0.85, 0.95) | | 0.27 (0.22, 0.32) | | NR |  |
| IL-6 | Farr, K; 2018 (71) | | | | Uganda | | 155 | | ORD | | | 107 | | Plasma, Frozen | | | Luminex | | NR | | | 0.90 (0.85, 0.95) | | 0.44 (0.37, 0.50) | | NR |  |
| IL-18 | Farr, K; 2018 (71) | | | | Uganda | | 155 | | ORD | | | 107 | | Plasma, Frozen | | | Luminex | | NR | | | 0.90 (0.85, 0.95) | | 0.13 (0.08, 0.19) | | NR |  |
| MDC | Farr, K; 2018 (71) | | | | Uganda | | 155 | | ORD | | | 107 | | Plasma, Frozen | | | Luminex | | NR | | | 0.90 (0.85, 0.95) | | 0.02 (0.00, 0.04) | | NR |  |
| MIG | Farr, K; 2018 (71) | | | | Uganda | | 155 | | ORD | | | 107 | | Plasma, Frozen | | | Luminex | | NR | | | 0.90 (0.85, 0.95) | | 0.16 (0.11, 0.21) | | NR |  |
| **HIV mixed positive and negative** | | | | | | | | | | | | | | | | | | | | | | | | | | |  |
| ADAMTS-13 | | Jacobs, R; 2016 (33) | | | | South Africa | 22 | | ORD | 33 | | | Plasma, Frozen | | | Luminex | | > 3466 ng/mL | | | 0.68 (0.45, 0.86) | | 0.64 (0.45, 0.80) | | 0.66 (0.51, 0.81) | |  |
| Apo-AI | | Jacobs, R; 2016 (33) | | | | South Africa | 22 | | ORD | 33 | | | Plasma, Frozen | | | Luminex | | < 318930 ng/mL | | | 0.73 (0.50, 0.89) | | 0.82 (0.65, 0.93) | | 0.76 (0.62, 0.89) | |  |
| AT-III | | Jacobs, R; 2016 (33) | | | | South Africa | 22 | | ORD | 33 | | | Plasma, Frozen | | | Luminex | | < 744162 ng/mL | | | 0.91 (0.71, 0.99) | | 0.61 (0.42, 0.77) | | 0.70 (0.56, 0.84) | |  |
| BDNF | | Jacobs, R; 2016 (33) | | | | South Africa | 22 | | ORD | 33 | | | Plasma, Frozen | | | Luminex | | < 3467 pg/mL | | | 0.45 (0.24, 0.68) | | 0.91 (0.76, 0.98) | | 0.69 (0.55, 0.84) | |  |
| bFGF | | Namuganga, A; 2023 (32) | | | | Uganda | 55 | | ORD | 106 | | | Serum, Frozen | | | Luminex | | 85 pg/mL | | | 0.87 (0.76, 0.94) | | 0.12 (0.07, 0.20) | | 0.63 (0.53, 0.73) | |  |
| CCL14 | | Jacobs, R; 2016 (33) | | | | South Africa | 22 | | ORD | 33 | | | Plasma, Frozen | | | Luminex | | > 136956 pg/mL | | | 0.59 (0.36, 0.79) | | 0.85 (0.68, 0.95) | | 0.75 (0.61, 0.89) | |  |
| CFH | | Jacobs, R; 2016 (33) | | | | South Africa | 22 | | ORD | 33 | | | Plasma, Frozen | | | Luminex | | > 808359 ng/mL | | | 0.68 (0.45, 0.86) | | 0.82 (0.65, 0.93) | | 0.72 (0.57, 0.86) | |  |
| CRP | | Calderwood, C; 2023 (62) | | | | South Africa | 255 | | ORD | 677 | | | Serum, Frozen | | | Turbidimetry | | 10 mg/L | | | 0.93 (0.89, 0.95) | | 0.54 (0.50, 0.58) | | 0.80 (0.77, 0.83) | |  |
|  |  | Calderwood, C; 2023 (62) | | | | South Africa | 255 | | ORD | 677 | | | Serum, Frozen | | | Turbidimetry | | 5 mg/L | | | 0.97 (0.94, 0.98) | | 0.39 (0.35, 0.42) | | 0.80 (0.77, 0.83) | |  |
|  |  | Jacobs, R; 2016 (33) | | | | South Africa | 22 | | ORD | 33 | | | Plasma, Frozen | | | Luminex | | > 9081 ng/mL  (9.1 mg/L) | | | 0.82 (0.60, 0.95) | | 0.9 (0.76, 0.98) | | 0.89 (0.79, 1.00) | |  |
|  |  | Koeppel, L; 2023 (39) | | | | Peru, South Africa, Vietnam | 177 | | ORD | 302 | | | Serum, Frozen | | | MSD | | NR | | | 0.75 (0.68, 0.81) | | 0.70 (0.64, 0.75) | | 0.73 (NR) | |  |
|  |  | Ruperez, M; 2023 (19) | | | | South Africa, Zambia | 76 | | ORD | 1231 | | | Whole blood, Fresh | | | Immunoassay  (POC setting) | | ≥5 mg/L | | | 0.50 (0.38, 0.62) | | 0.72 (0.70, 0.75) | | 0.61 (0.55, 0.67) | |  |
|  |  | Ruperez, M; 2023 (19) | | | | South Africa, Zambia | 76 | | ORD | 1231 | | | Whole blood, Fresh | | | Immunoassay  (POC setting) | | ≥10 mg/L | | | 0.36 (0.25, 0.47) | | 0.86 (0.84, 0.88) | | 0.61 (0.55, 0.67) | |  |
|  |  | Samuels, T; 2021 (64) | | | | Peru, South Africa, Vietnam, Cambodia, Georgia | 391 | | ORD | 374 | | | Serum, Frozen | | | ELISA | | ≥ 10 mg/L | | | 0.78 (0.73, 0.82) | | 0.66 (0.61, 0.71) | | 0.77 (0.74, 0.81) | |  |
|  |  | Wilson, D; 2011 (31) | | | | South Africa | 135 | | ORD | 115 | | | Serum,  NR | | | Turbidimetry | | ≥5 mg/L | | | 0.98 (0.94, 0.99) | | 0.59 (0.50, 0.68) | | 0.91 (0.87, 0.95) | |  |
|  |  | Wilson, D; 2011 (31) | | | | South Africa | 135 | | ORD | 115 | | | Serum,  NR | | | Turbidimetry | | ≥ 10 mg/L | | | **0.95 (0.90, 0.98)** | | **0.77 (0.69, 0.85)** | | 0.91 (0.87, 0.95) | |  |
| CXCL11 | | Jacobs, R; 2016 (33) | | | | South Africa | 22 | | ORD | 33 | | | Plasma, Frozen | | | Luminex | | > 276.5 pg/mL | | | 0.95 (0.77, 1.0) | | 0.36 (0.20, 0.55) | | 0.68 (0.54, 0.83) | |  |
| Eotaxin | | Namuganga, A; 2023 (32) | | | | Uganda | 55 | | ORD | 106 | | | Serum, Frozen | | | Luminex | | 1887 pg/mL | | | 0.91 (0.80, 0.96) | | 0.11 (0.06, 0.19) | | 0.64 (0.54, 0.73) | |  |
| FAS | | Jacobs, R; 2016 (33) | | | | South Africa | 22 | | ORD | 33 | | | Plasma, Frozen | | | Luminex | | > 6.7 ng/mL | | | 0.67 (0.43, 0.85) | | 0.68 (0.49, 0.83) | | 0.65 (0.50, 0.81) | |  |
| Ferritin | | Jacobs, R; 2016 (33) | | | | South Africa | 22 | | ORD | 33 | | | Plasma, Frozen | | | Luminex | | > 93785 pg/mL | | | 0.91 (0.71, 0.99) | | 0.67 (0.48, 0.82) | | 0.78 (0.64, 0.92) | |  |
| GDF-15 | | Jacobs, R; 2016 (33) | | | | South Africa | 22 | | ORD | 33 | | | Plasma, Frozen | | | Luminex | | > 21.06 ng/mL | | | 0.91 (0.71, 0.99) | | 0.55 (0.36, 0.72) | | 0.75 (0.62, 0.88) | |  |
| GM-CSF | | Namuganga, A; 2023 (32) | | | | Uganda | 55 | | ORD | 106 | | | Serum, Frozen | | | Luminex | | 147 pg/mL | | | 0.82 (0.70, 0.90) | | 0.40 (0.31, 0.49) | | 0.67 (0.58, 0.77) | |  |
| I-309 | | Jacobs, R; 2016 (33) | | | | South Africa | 22 | | ORD | 33 | | | Plasma, Frozen | | | Luminex | | > 1.945 pg/mL | | | 0.68 (0.45, 0.86) | | 0.90 (0.73, 0.98) | | 0.8 (0.67, 0.93) | |  |
|  |  | Koeppel, L; 2023 (39) | | | | Peru, South Africa, Vietnam | 177 | | ORD | 302 | | | Serum, Frozen | | | MSD | | NR | | | 0.81 (0.74, 0.86) | | 0.70 (0.64, 0.75) | | 0.87 (NR) | |  |
| IFN-γ | | Jacobs, R; 2016 (33) | | | | South Africa | 22 | | ORD | 33 | | | Plasma, Frozen | | | Luminex | | > 3.910 pg/mL | | | 0.91 (0.70, 0.99) | | 0.48 (0.31, 0.66) | | 0.69 (0.54, 0.83) | |  |
|  |  | Namuganga, A; 2023 (32) | | | | Uganda | 55 | | ORD | 106 | | | Serum, Frozen | | | Luminex | | 102 pg/mL | | | 0.91 (0.80, 0.96) | | 0.14 (0.09, 0.22) | | 0.61 (0.51, 0.70) | |  |
| IL-1β | | Namuganga, A; 2023 (32) | | | | Uganda | 55 | | ORD | 106 | | | Serum, Frozen | | | Luminex | | 143 pg/mL | | | 0.84 (0.72, 0.91) | | 0.53 (0.43, 0.62) | | 0.74 (0.66, 0.83) | |  |
| IL-2 | | Namuganga, A; 2023 (32) | | | | Uganda | 55 | | ORD | 106 | | | Serum, Frozen | | | Luminex | | 95 pg/mL | | | 0.80 (0.68, 0.88) | | 0.48 (0.39, 0.58) | | 0.68 (0.59, 0.77) | |  |
| IL-6 | | Namuganga, AR; 2017 (81) | | | | Uganda | 39 | | ORD | 39 | | | Serum, Frozen | | | Luminex | | > 36.4 pg/mL | | | 0.46 (0.30, 0.63) | | 0.97 (0.87, 0.99) | | 0.85 (NR) | |  |
|  |  | Namuganga, A; 2023 (32) | | | | Uganda | 55 | | ORD | 106 | | | Serum, Frozen | | | Luminex | | 187 pg/mL | | | 0.87 (0.76, 0.94) | | 0.63 (0.54, 0.72) | | 0.83 (0.77, 0.90) | |  |
| IL-8 | | Namuganga, A; 2023 (32) | | | | Uganda | 55 | | ORD | 106 | | | Serum, Frozen | | | Luminex | | 131 pg/mL | | | 0.91 (0.80, 0.96) | | 0.12 (0.07, 0.20) | | 0.64 (0.55, 0.74) | |  |
| IL-33 | | Jacobs, R; 2016 (33) | | | | South Africa | 22 | | ORD | 33 | | | Plasma, Frozen | | | Luminex | | > 131.8 pg/mL | | | 0.68 (0.45, 0.86) | | 0.61 (0.42, 0.77) | | 0.63 (0.48, 0.78) | |  |
| IL12p70 | | Namuganga, A; 2023 (32) | | | | Uganda | 55 | | ORD | 106 | | | Serum, Frozen | | | Luminex | | 65 pg/mL | | | 0.82 (0.70, 0.90) | | 0.52 (0.42, 0.61) | | 0.69 (0.60, 0.78) | |  |
| IP-10 | | Jacobs, R; 2016 (33) | | | | South Africa | 22 | | ORD | 33 | | | Plasma, Frozen | | | Luminex | | > 746.6 pg/mL | | | 0.86 (0.65, 0.97) | | 0.73 (0.54, 0.87) | | 0.78 (0.64, 0.91) | |  |
|  |  | Namuganga, A; 2023 (32) | | | | Uganda | 55 | | ORD | 106 | | | Serum, Frozen | | | Luminex | | 4087 pg/mL | | | **0.93 (0.82, 0.97)** | | **0.81 (0.73, 0.87)** | | 0.89 (0.84, 0.94) | |  |
| MIG | | Jacobs, R; 2016 (33) | | | | South Africa | 22 | | ORD | 33 | | | Plasma, Frozen | | | Luminex | | > 1700 pg/mL | | | 0.68 (0.45, 0.86) | | 0.88 (0.72, 0.97) | | 0.81 (0.69, 0.94) | |  |
| MIP-1β | | Jacobs, R; 2016 (33) | | | | South Africa | 22 | | ORD | 33 | | | Plasma, Frozen | | | Luminex | | > 212263 ng/mL | | | 0.32 (0.14, 0.55) | | 0.97 (0.84, 1.0) | | 0.63 (0.48, 0.79) | |  |
| MIP-4 | | Jacobs, R; 2016 (33) | | | | South Africa | 22 | | ORD | 33 | | | Plasma, Frozen | | | Luminex | | > 220.9 pg/mL | | | 0.50 (0.28, 0.72) | | 0.91 (0.76, 0.98) | | 0.70 (0.55, 0.85) | |  |
| NCAM-1 | | Jacobs, R; 2016 (33) | | | | South Africa | 22 | | ORD | 33 | | | Plasma, Frozen | | | Luminex | | < 477229 pg/mL | | | **0.91 (0.71, 0.99)** | | **0.73 (0.54, 0.87)** | | 0.88 (0.78, 0.98) | |  |
| NGAL | | Jacobs, R; 2016 (33) | | | | South Africa | 22 | | ORD | 33 | | | Plasma, Frozen | | | Luminex | | > 552.8 ng/mL | | | 0.59 (0.36, 0.79) | | 0.76 (0.58, 0.89) | | 0.65 (0.50, 0.80) | |  |
| PCT | | Jacobs, R; 2016 (33) | | | | South Africa | 22 | | ORD | 33 | | | Plasma, Frozen | | | Luminex | | > 8101 pg/mL | | | 0.86 (0.65, 0.97) | | 0.67 (0.48, 0.82) | | 0.77 (0.64, 0.90) | |  |
| PEDF | | Jacobs, R; 2016 (33) | | | | South Africa | 22 | | ORD | 33 | | | Plasma, Frozen | | | Luminex | | > 11423 pg/mL | | | 0.68 (0.45, 0.86) | | 0.64 (0.45, 0.80) | | 0.66 (0.50, 0.81) | |  |
| P-selectin | | Jacobs, R; 2016 (33) | | | | South Africa | 22 | | ORD | 33 | | | Plasma, Frozen | | | Luminex | | > 265.7 ng/mL | | | 0.77 (0.55, 0.92) | | 0.58 (0.39, 0.75) | | 0.67 (0.53, 0.82) | |  |
| RANTES | | Namuganga, A; 2023 (32) | | | | Uganda | 55 | | ORD | 106 | | | Serum, Frozen | | | Luminex | | >70,173 pg/mL | | | 0.82 (0.70, 0.90( | | 0.61 (0.52, 0.70) | | 0.67 (0.58, 0.77) | |  |
| SAA | | Jacobs, R; 2016 (33) | | | | South Africa | 22 | | ORD | 33 | | | Plasma, Frozen | | | Luminex | | > 8626 ng/mL | | | 0.68 (0.45, 0.86) | | 0.70 (0.51, 0.84) | | 0.71 (0.58, 0.85) | |  |
| SAP | | Jacobs, R; 2016 (33) | | | | South Africa | 22 | | ORD | 33 | | | Plasma, Frozen | | | Luminex | | > 25958 ng/mL | | | 0.68 (0.45, 0.86) | | 0.85 (0.68, 0.95) | | 0.85 (0.72, 0.98) | |  |
| TNF | | Jacobs, R; 2016 (33) | | | | South Africa | 22 | | ORD | 33 | | | Plasma, Frozen | | | Luminex | | > 10.85 pg/mL | | | 0.82 (0.60, 0.95) | | 0.73 (0.54, 0.87) | | 0.74 (0.61, 0.88) | |  |
|  |  | Namuganga, A; 2023 (32) | | | | Uganda | 55 | | ORD | 106 | | | Serum, Frozen | | | Luminex | | 255 pg/mL | | | 0.82 (0.70, 0.90) | | 0.36 (0.27, 0.45) | | 0.65 (0.56, 0.75) | |  |
| TPA | | Jacobs, R; 2016 (33) | | | | South Africa | 22 | | ORD | 33 | | | Plasma, Frozen | | | Luminex | | > 6307 pg/mL | | | 0.86 (0.65, 0.97) | | 0.76 (0.58, 0.89) | | 0.80 (0.68, 0.92) | |  |
| TTR | | Jacobs, R; 2016 (33) | | | | South Africa | 22 | | ORD | 33 | | | Plasma, Frozen | | | Luminex | | < 416242 ng/mL | | | 0.82 (0.60, 0.95) | | 0.76 (0.58, 0.89) | | 0.78 (0.65, 0.91) | |  |
| VEGF | | Jacobs, R; 2016 (33) | | | | South Africa | 22 | | ORD | 33 | | | Plasma, Frozen | | | Luminex | | > 175.6 pg/mL | | | 0.73 (0.50, 0.89) | | 0.55 (0.36, 0.72) | | 0.64 (0.50, 0.79) | |  |
|  |  | Namuganga, A; 2017 (81) | | | | Uganda | 39 | | ORD | 39 | | | Serum, Frozen | | | Luminex | | > 573.8 pg/mL | | | 0.28 (0.15, 0.44) | | 0.97 (0.87, 0.99) | | 0.71 (NR) | |  |
|  |  | Namuganga, A; 2023 (32) | | | | Uganda | 55 | | ORD | 106 | | | Serum, Frozen | | | Luminex | | 157 pg/mL | | | 0.87 (0.76, 0.94) | | 0.19 (0.13, 0.27) | | 0.66 (0.57, 0.76) | |  |
| **HIV status not reported** | | | | | | | | | | | | | | | | | | | | | | | | | | |  |
| CA-125 | | Du, Z; 2017 (68) | | | | China | 59* | | HC | 48 | | | Serum,  NR | | | Electrochemi-luminescent Immunoassay | | 13.6 IU/mL | | | 0.88 (0.77, 0.95) | | 0.96 (0.86, 0.99) | | 0.96 (0.91, 0.99) | |  |
|  |  | Du, Z; 2017 (68) | | | | China | 102 | | HC | 48 | | | Serum,  NR | | | Electrochemi-luminescent Immunoassay | | 13.6 IU/mL | | | 0.85 (0.77, 0.92) | | 0.96 (0.86, 0.99) | | 0.94 (0.88, 0.97) | |  |
| CEA | | Du, Z; 2017 (68) | | | | China | 102 | | HC | 48 | | | Serum,  NR | | | Electrochemi-luminescent Immunoassay | | 1.84 ng/mL | | | 0.72 (0.62, 0.81) | | 0.61 (0.45, 0.76) | | 0.61 (0.53, 0.69) | |  |
|  |  | Du, Z; 2017 (68) | | | | China | 59* | | HC | 48 | | | Serum,  NR | | | Electrochemi-luminescent Immunoassay | | 1.84 ng/mL | | | 0.83 (0.71, 0.92) | | 0.61 (0.45, 0.76) | | 0.72 (0.63, 0.81) | |  |
| *Du, 2017 and Kumar, 2018: all TB cases had Diabetes mellitus | | | | | | | | | | | | | | | | | | | | | | | | | | |  |

| **Table S6. Biomarker signatures for adult pulmonary TB, by HIV status**  Biomarkers meeting TPP criteria are indicated in bold  AUC=area under the curve, DM=diabetes mellitus, ELISA=enzyme-linked immunosorbent assay, HC= healthy controls, LTBI=latent TB infection, MSD=Meso Scale Discovery, N=sample size, ORD=other respiratory diseases, NR=not reported | | | | | | | | | | | |
| --- | --- | --- | --- | --- | --- | --- | --- | --- | --- | --- | --- |
| **Biomarker** | **Study ID** | **Country** | **TB cases, N** | **Control group** | **Control group, N** | **Sample type, condition** | **Testing method** | **Biomarker cut-off(unit)** | **Sensitivity**  **(95% CI)** | **Specificity**  **(95% CI)** | **AUC (95% CI)** |
| **HIV-negative** | | | | | | | | | | | |
| C1q, C1-INH | Lubbers, R; 2020 ([77](#_ENREF_74)) | the Gambia | 50 | HC | 50 | Serum, Frozen | ELISA | NR | 0.36 (0.23, 0.51) | 0.96 (0.86, 0.99) | 0.66 (0.55, 0.77) |
| CALCOCO2, IFITM3, SAMD9L | Garlant, H; 2022 ([21](#_ENREF_21)) | India, United Kingdom | 49 | TB Contacts | 209 | Whole blood, Frozen | ELISA | NR | **0.92 (0.82, 0.97)** | **0.99 (0.96, 0.99)** | 0.99 (0.98, 1.0) |
| CLEC3B, ECM1, IP10, SELL | Ndiaye, M; 2022 ([22](#_ENREF_22)) | Madagascar | 37 | LTBI | 24 | Plasma, Frozen | Luminex | NR | 0.89 (0.75, 0.96) | 0.91 (0.74, 0.98) | 0.93 (NR) |
| HO-1, MMP-1 | Andrade, B; 2015 ([26](#_ENREF_26)) | Brazil | 63 | LTBI | 15 | Plasma, Frozen | ELISA, Luminex | HO-1 > 2.397 ng/mL; MMP-1 > 3.511 ng/mL | **0.95 (0.87, 0.99)** | **0.93 (0.68, 0.99)** | 0.98 (0.94, 1.0) |
| HO-1, MMP-1 | Andrade, B; 2015 ([26](#_ENREF_26)) | India | 97 | LTBI | 39 | Plasma, Frozen | ELISA, Luminex | HO-1 > 1.65 ng/mL; MMP-1 > 1.21 ng/mL | **0.98 (0.93, 0.99)** | **1 (0.77, 1.0)** | 1.0 (1.0, 1.0) |
| I-309, MIG, IL-8 | Chen, T; 2015 ([67](#_ENREF_64)) | China | 78 | ORD | 49 | Serum,  NR | ELISA | NR | 0.56 (0.45, 0.68) | 0.98 (0.89, 0.99) | NR |
| IP-10, CXCL13 | Estevez, O; 2020 (70) | Spain | 28 | LTBI | 27 | Serum, Frozen | Luminex | NR | 0.72 (0.53, 0.85) | 0.88 (0.72, 0.96) | 0.83 (NR) |
| IP-10, CXCL13, IL-7 | Estevez, O; 2020 (70) | Spain | 28 | LTBI | 27 | Serum, Frozen | Luminex | NR | 0.72 (0.53, 0.85) | 0.68 (0.48, 0.81) | 0.79 (NR) |
| Pla2G2A, CK-MB, HEPC | Essone, P; 2022 (69) | Gabon | 26 | ORD | 36 | Serum, Frozen | ELISA |  | 0.74 (0.52, 0.88) | 0.88 (0.74, 0.97) | 0.84 (NR) |
| SYWC, kallistatin, C9, gelsolin, testican-2, aldolase C | De Groote, M; 2017 ([34](#_ENREF_34)) | Peru, South Africa, Vietnam, Bangladesh, Zimbabwe, Columbia | 177 | ORD | 226 | Serum, Frozen | SomaScan | NR | 0.88 (0.81, 0.92) | 0.89 (0.86, 0.90) | 0.94 (0.92, 0.96) |
| **HIV-positive** | | | | | | | | | | | |
| IFN-γ, IL-6 | Farr, K; 2018 (71) | Uganda | 155 | ORD | 107 | Plasma, Frozen | Luminex | NR | 0.90 (0.85, 0.95) | 0.50 (0.47, 0.53) | 0.84 (NR) |
| SYWC, kallistatin, C9, gelsolin, testican-2, aldolase C | De Groote, M; 2017 ([34](#_ENREF_34)) | Peru, South Africa, Vietnam, Bangladesh, Zimbabwe, Columbia | 85 | ORD | 81 | Serum, Frozen | SomaScan | NR | 0.89 (0.82, 0.92) | 0.83 (0.73, 0.88) | 0.93 (0.87, 0.96) |
| **Mixed HIV-positive and negative** | | | | | | | | | | | |
| Apo-AI, CFH, TTR, CRP, INF-γ, SAA, IP-10 | Morris, T; 2021 ([35](#_ENREF_35)) | South Africa, Malawi | 122 | ORD | 127 | Serum, Frozen | Luminex | NR | 0.98 (0.94, 1.0) | 0.12 (0.07, 0.19) | NR |
| CFH, IP-10, CRP, SAA, TTR | Morris, T; 2021 ([35](#_ENREF_35)) | South Africa, Malawi | 37 | ORD | 38 | Serum, Frozen | Luminex | NR | 0.79 (0.63, 0.90) | 0.41 (0.25, 0.58) | NR |
| Fibrinogen, α-2-M, CRP, MMP-9, TTR, CFH, IFN-γ, IP-10, TNF | Morris, T; 2021 ([35](#_ENREF_35)) | South Africa, Malawi | 37 | ORD | 38 | Serum, Frozen | Luminex | NR | **0.92 (0.80, 0.98)** | **0.71 (0.56, 0.84)** | 0.84 (0.73, 0.94) |
| IL-6, IL-8, IL-18 | Ahmad, R; 2019 ([66](#_ENREF_62)) | Peru, South Africa, Vietnam | 160 | ORD and LTBI | 157 | Serum, Frozen | Simoa assay | NR | 0.79 (0.72, 0.84) | 0.68 (0.60, 0.74) | 0.80 (0.76, 0.85) |
| IL-6, IL-8, IL-18, VEGF | Ahmad, R; 2019 ([66](#_ENREF_62)) | Peru, South Africa, Vietnam | 160 | ORD and LTBI | 157 | Serum, Frozen | Simoa assay | NR | 0.80 (0.73, 0.85) | 0.65 (0.57, 0.71) | 0.80 (0.75, 0.85) |
| SYWC, kallistatin, C9, gelsolin, testican-2, aldolase C | De Groote, M; 2017 ([34](#_ENREF_34)) | Peru, South Africa, Vietnam, Bangladesh, Zimbabwe, Columbia | 80 | ORD | 97 | Serum, Frozen | SomaScan | NR | 0.85 (0.75, 0.92) | 0.89 (0.81, 0.94) | 0.92 (0.87, 0.96) |

| **Table S7: Biomarker and signatures for adult pulmonary TB, reporting only AUC**  Biomarkers likely to meet TPP criteria are indicated in bold  AUC=area under the curve, DM=diabetes mellitus, ELISA=enzyme-linked immunosorbent assay, HC= healthy controls, LTBI=latent TB infection, MSD=Meso Scale Discovery, N=sample size, ORD=other respiratory diseases, NR=not reported | | | | | | | | | |
| --- | --- | --- | --- | --- | --- | --- | --- | --- | --- |
| **Biomarker** | **Study ID** | **Country** | **HIV status** | **TB cases, N** | **Control group** | **Control group, N** | **Sample type, condition** | **Testing method** | **AUC (95% CI)** |
| α-2-M | Morris, T; 2021 (35) | South Africa, Malawi | positive and negative | 146 | ORD | 146 | Serum, Frozen | Luminex | 0.57 (0.50, 0.64) |
| Apo-AI | Koeppel, L; 2023 (39) | Peru, South Africa, Vietnam | positive and negative | 177 | ORD | 302 | Serum, Frozen | MSD | 0.5 (NR) |
|  | Morris, T; 2021 (35) | South Africa, Malawi | positive and negative | 146 | ORD | 146 | Serum, Frozen | Luminex | 0.52 (0.45, 0.59) |
| Apo-CIII | Morris, T; 2021 (35) | South Africa, Malawi | positive and negative | 146 | ORD | 146 | Serum, Frozen | Luminex | 0.58 (0.51, 0.64) |
| C1q | Lubbers, R; 2018 (36) | South Africa, Italy, the Gambia, Korea, Netherlands | negative | 99 | ORD | 68 | Serum,  NR | ELISA | **0.93 (0.89, 0.97)** |
| CFH | Morris, T; 2021 (35) | South Africa, Malawi | positive and negative | 146 | ORD | 146 | Serum, Frozen | Luminex | 0.70 (0.64, 0.76) |
| CRP | Morris, T; 2021 (35) | South Africa, Malawi | positive and negative | 146 | ORD | 146 | Serum, Frozen | Luminex | 0.51 (0.43, 0.58) |
| CXCL1 | Sampath, P; 2023b (83) | India | Negative | 40 DS-TB; 40 DR-TB | LTBI | 40 | Plasma, Frozen | Luminex | 0.85 (NR) DS-TB  0.80 (NR) DR-TB |
| CXCL11 | Sampath, P; 2023b (83) | India | Negative | 40 DR-TB | HC | 40 | Plasma, Frozen | Luminex | 0.82 (NR) |
| Ferretin | Luo, Y; 2020 (78) | China | negative | 66 | LTBI | 53 | Serum,  NR | Turbidimetry | 0.63 (NR) |
|  | Morris, T; 2021 (35) | South Africa, Malawi | positive and negative | 146 | ORD | 146 | Serum, Frozen | Luminex | 0.57 (0.50, 0.64) |
| Fibrinogen | Morris, T; 2021 (35) | South Africa, Malawi | positive and negative | 146 | ORD | 146 | Serum, Frozen | Luminex | 0.56 (0.49, 0.63) |
| HP | Morris, T; 2021 (35) | South Africa, Malawi | positive and negative | 146 | ORD | 146 | Serum, Frozen | Luminex | 0.64 (0.58, 0.71) |
| I-309 | Chen, T; 2015 (67) | China | negative | 81 | HC and/or ORD | 162 | Serum,  NR | ELISA | **0.90 (NR)** |
|  | Sampath, P; 2023b (83) | India | negative | 40 DS-TB; 40 DR-TB | HC | 40 | Plasma, Frozen | Luminex | 0.85 (NR) DS-TB  0.88 (NR) DR-TB |
| IFN-α-2 | Morris, T; 2021 (35) | South Africa, Malawi | positive and negative | 146 | ORD | 146 | Serum, Frozen | Luminex | 0.52 (0.45, 0.58) |
| IFN-γ | Morris, T; 2021 (35) | South Africa, Malawi | positive and negative | 146 | ORD | 146 | Serum, Frozen | Luminex | 0.66 (0.60, 0.72) |
|  | Sampath, P; 2023a (38) | India | negative | 40 DS-TB | HC | 40 | Plasma, Frozen | Luminex | **0.95 (NR)** |
|  | Sampath, P; 2023a (38) | India | negative | 40 DR-TB | LTBI | 40 | Plasma, Frozen | Luminex | **0.94 (NR)** |
| IL-2 | Sampath, P; 2023a (38) | India | negative | 40 DS-TB;  40 DR-TB | HC | 40 | Plasma, Frozen | Luminex | **1.0 (NR) DS-TB**  **1.0 (NR) DR-TB** |
| IL-5 | Sampath, P; 2023a (38) | India | negative | 40 DS-TB | LTBI | 40 | Plasma, Frozen | Luminex | **0.92 (NR)** |
| IL-6 | Sampath, P; 2023a (38) | India | negative | 40 DR-TB | LTBI | 40 | Plasma, Frozen | Luminex | **0.93 (NR)** |
| IL-8 | Chen, T; 2015 (67) | China | negative | 81 | HC and/or ORD | 162 | Serum,  NR | ELISA | 0.81 (NR) |
| IL-10 | Sampath, P; 2023a (38) | India | negative | 40 DS-TB; 40 DR-TB | LTBI | 40 | Plasma, Frozen | Luminex | **0.90 (NR) DS-TB**  **1.0 (NR) DR-TB** |
| IL-17A | Sampath, P; 2023a (38) | India | negative | 40 DS-TB;  40 DR-TB | LTBI | 40 | Plasma, Frozen | Luminex | **0.90 (NR) DS-TB**  **1.0 (NR) DR-TB** |
| IL-1α | Sampath, P; 2023a (38) | India | negative | 40 DS-TB | LTBI | 40 | Plasma, Frozen | Luminex | **0.97 (NR)** |
| IL-1RA | Morris, T; 2021 (35) | South Africa, Malawi | positive and negative | 146 | ORD | 146 | Serum, Frozen | Luminex | 0.51 (0.44, 0.58) |
| IP-10 | Koeppel, L; 2023 (39) | Peru, South Africa, Vietnam | positive and negative | 177 | ORD | 302 | Serum, Frozen | MSD | 0.79 (NR) |
|  | Morris, T; 2021 (35) | South Africa, Malawi | positive and negative | 146 | ORD | 146 | Serum, Frozen | Luminex | 0.66 (0.60, 0.73) |
|  | Sampath, P; 2023b (83) | India | negative | 40 DS-TB;  40 DR-TB | LTBI | 40 | Plasma, Frozen | Luminex | **0.94 (NR) DS-TB**  **0.98 (NR) DR-TB** |
| MCP-1 | Sampath, P; 2023b (83) | India | negative | 40 DR-TB | HC | 40 | Plasma, Frozen | Luminex | 0.88 (NR) |
| MICA | Jiang, X; 2012 (73) | China | negative | 55 | HC | 141 | Serum,  NR | ELISA | 0.77 (NR) |
| MIG | Chen, T; 2015 (67) | China | negative | 81 | HC and/or ORD | 162 | Serum,  NR | ELISA | 0.87 (NR) |
|  | Koeppel, L; 2023 (39) | Peru, South Africa, Vietnam | positive and negative | 177 | ORD | 302 | Serum, Frozen | MSD | 0.83 (NR) |
|  | Sampath, P; 2023b (83) | India | negative | 40 DS-TB;  40 DR-TB | LTBI | 40 | Plasma, Frozen | Luminex | **0.92 (NR) DS-TB**  **0.98 (NR) DR-TB** |
| MMP-2 | Morris, T; 2021 (35) | South Africa, Malawi | positive and negative | 146 | ORD | 146 | Serum, Frozen | Luminex | 0.52 (0.45, 0.58) |
| MMP-9 | Morris, T; 2021 (35) | South Africa, Malawi | positive and negative | 146 | ORD | 146 | Serum, Frozen | Luminex | 0.53 (0.47, 0.60) |
| PCT | Morris, T; 2021 (35) | South Africa, Malawi | positive and negative | 146 | ORD | 146 | Serum, Frozen | Luminex | 0.52 (0.45, 0.59) |
| SAA | Morris, T; 2021 (35) | South Africa, Malawi | positive and negative | 146 | ORD | 146 | Serum, Frozen | Luminex | 0.65 (0.58, 0.71) |
| SAP | Morris, T; 2021 (35) | South Africa, Malawi | positive and negative | 146 | ORD | 146 | Serum, Frozen | Luminex | 0.64 (0.57, 0.71) |
| SYWC | Koeppel, L; 2023 (39) | Peru, South Africa, Vietnam | positive and negative | 177 | ORD | 302 | Serum, Frozen | MSD | 0.86 (NR) |
| TfR | Luo, Y; 2020 (78) | China | negative | 66 | LTBI | 53 | Serum,  NR | Turbidimetry | 0.66 (NR) |
| TGF-α | Morris, T; 2021 (35) | South Africa, Malawi | positive and negative | 146 | ORD | 146 | Serum, Frozen | Luminex | 0.55 (0.49, 0.62) |
| TNF | Morris, T; 2021 (35) | South Africa, Malawi | positive and negative | 146 | ORD | 146 | Serum, Frozen | Luminex | 0.53 (0.46, 0.59) |
|  | Namuganga, AR; 2017 (81) | Uganda | positive and negative | 39 | ORD | 39 | Serum, Frozen | Luminex | 0.63 (NR) |
|  | Sampath, P; 2023a (38) | India | negative | 40 DR-TB | LTBI | 40 | Plasma, Frozen | Luminex | **0.93 (NR)** |
| TPA | Morris, T; 2021 (35) | South Africa, Malawi | positive and negative | 146 | ORD | 146 | Serum, Frozen | Luminex | 0.57 (0.50, 0.64) |
| Transferrin | Luo, Y; 2020 (78) | China | negative | 66 | LTBI | 53 | Serum,  NR | Turbidimetry | 0.71 (NR) |
| TTR | Morris, T; 2021 (35) | South Africa, Malawi | positive and negative | 146 | ORD | 146 | Serum, Frozen | Luminex | 0.61 (0.55, 0.68) |
| VEGF | Morris, T; 2021 (35) | South Africa, Malawi | positive and negative | 146 | ORD | 146 | Serum, Frozen | Luminex | 0.64 (0.57, 0.71) |
| **Signatures** | | | | | | | | | |
| CD14, SEPP1, SELL, TNXB, LUM, PEPD, QSOX1, COMP, Apo CI, GP1BA | Halliday, A; 2021 (72) | United Kingdom | negative | 60 | ORD | 84 | Serum, Frozen | Mass spectrometry | 0.70 (0.62, 0.79) |
| I-309, SYWC, kallistatin | Koeppel, L; 2023 (39) | Peru, South Africa, Vietnam | positive and negative | 177 | ORD | 302 | Serum, Frozen | MSD | **0.90 (NR)** |
| S100A9, SOD3,  MMP-9 | Halliday, A; 2021 (72) | United Kingdom | negative | 60 | ORD | 84 | Serum, Frozen | Mass spectrometry | 0.70 (0.61, 0.79) |
| SYWC, I-309 | Koeppel, L; 2023 (39) | Peru, South Africa, Vietnam | positive | 46 | ORD | 240 | Serum, Frozen | MSD | **0.91 (NR)** |
| SYWC, kallistatin | Koeppel, L; 2023 (39) | Peru, South Africa, Vietnam | positive and negative | 177 | ORD | 302 | Serum, Frozen | MSD | 0.88 (NR) |

| **Table S8: Composite reference standards for adult EPTB and childhood TB** | |
| --- | --- |
| **Study ID** | **Proportion of TB patients positive on MRS** |
| **EPTB** | |
| Abhimanyu 2016 (87) | 26% positive on culture |
| Andrade, B; 2013 (25) | All positive on AFB smear or culture |
| Chen, X; 2023 (37) | 53% positive on Xpert, 43% positive on NAAT, 3% positive on culture |
| de Larrea, C; 2011 (88) | 70% positive on culture |
| Garlant, H; 2022 (21) | All positive on AFB smear and culture |
| Goyal, N; 2016 (45) | 13% positive on culture |
| He, X; 2020 (89) | 1.37% positive on culture |
| Kathamuthu, G; 2020 (29) | unclear |
| Kim, J; 2016 (90) | 46% positive on culture or AFB smear or PCR |
| Lin, L; 2021 (42) | unclear |
| Liu, Q; 2019 (91) | 28.6% smear or culture positive in sputum; 10.7% AFB smear or culture positive in pleural effusion |
| Lou, C; 2022 (92) | All positive on AFB or culture |
| Mann, T; 2021 (44) | 69% positive on culture or Xpert |
| Onur, S; 2015 (43) | unclear |
| Wang, J; 2022 (93) | All positive on AFB or culture |
| **Childhood TB** | |
| Kumar, NP; 2021b (47) | All positive on culture, Xpert or AFB smear |
| Kashyap, B; 2020 (46) | 55% positive on AFB smear, culture or NAAT |
| Manyelo, C; 2019 (94) | 6.4% positive on AFB smear, culture or NAAT |

**Table S9: Summary of adult EPTB biomarkers and signatures that meet the TPP criteria**

Legend: Control groups: HC = healthy control, LTBI = latent TB infection, OD = other disease. 2-marker signature: CFHR2, CFHR3; 5-marker signature: CRP, NCAM, Ferritin, IL-8, GDF-15. QUADAS-2 was not done for EP-TB studies

| **Table S10: Biomarkers and signatures for adult extrapulmonary TB**  Biomarkers meeting TPP criteria are indicated in bold  AUC=area under the curve, ELISA=enzyme-linked immunosorbent assay, HC= healthy controls, LTBI=latent TB infection, N=sample size, ORD=other respiratory diseases, NR=not reported | | | | | | | | | | | | | |
| --- | --- | --- | --- | --- | --- | --- | --- | --- | --- | --- | --- | --- | --- |
| **Biomarker** | **Study ID** | **Country** | **Site of disease** | **HIV-status** | **TB cases, N** | **Control group** | **Control group, N** | **Sample type** | **Testing method** | **Biomarker cut-off (unit)** | **Sensitivity**  **(95% CI)** | **Specificity**  **(95% CI)** | **AUC (95% CI)** |
| ADA | He, X; 2020 (89) | China | Abdominal | negative | 73 | OD + HC | 135 | Serum,  NR | Peroxidase method | 15.31 IU/mL | 0.74 (0.62, 0.84) | 0.75 (0.67, 0.82) | 0.73 (0.65, 0.80) |
| Apo-AI | Mann, T; 2021 (44) | South Africa | Bone | positive and negative | 26 | OD | 17 | Serum,  Frozen | Luminex | NR | 0.71 (0.44, 0.90) | 0.54 (0.33, 0.73) | 0.61 (0.43, 0.80) |
| Apo-CII | Chen, X; 2023 (37) | China | Bone | negative | 30 | OD | 30 | Serum, Frozen | ELISA | 7.788 μg/mL | **0.97 (0.83, 0.99)** | **0.93 (0.78, 0.99)** | NR |
| Apo-E | Chen, X; 2023 (37) | China | Bone | negative | 30 | OD | 30 | Serum, Frozen | ELISA | 49.46 μg/mL | 0.60 (0.42, 0.75) | 0.57 (0.39, 0.73) | NR |
| CA-125 | He, X; 2020 (89) | China | Abdominal | negative | 73 | OD + HC | 135 | Serum,  NR | Electroche- miluminescent Immunoassay | 30.34 IU/mL | 0.85 (0.75, 0.92) | 0.60 (0.51, 0.68) | 0.72 (0.65, 0.79) |
| CALCOCO2 | Garlant, H; 2022 (21) | India, United Kingdom | NR | negative | 82 | LTBI | 111 | Whole blood, Frozen | ELISA | 990.7 ng/mL | 0.90 (0.83, 0.95) | 0.40 (0.32, 0.49) | 0.72 (0.66, 0.78) |
| CD163 | Liu, Q; 2019 (91) | China | Pleural | negative | 28 | OD | 21 | Plasma, Frozen | ELISA | 934.7 ng/mL | 0.77 (0.59, 0.92) | 0.80 (0.58, 0.95) | 0.81 (NR) |
| CD52 | Garlant, H; 2022 (21) | India, United Kingdom | NR | negative | 82 | LTBI | 111 | Whole blood, Frozen | ELISA | 964.8 ng/mL | 0.90 (0.83, 0.95) | 0.14 (0.09, 0.21) | 0.59 (0.52, 0.66) |
| CFD | Mann, T; 2021 (44) | South Africa | Bone | positive and negative | 26 | OD | 17 | Serum,  Frozen | Luminex | NR | 0.59 (0.33, 0.82) | 0.65 (0.44, 0.83) | 0.62 (0.44, 0.79) |
| CFH | Mann, T; 2021 (44) | South Africa | Bone | positive and negative | 26 | OD | 17 | Serum,  Frozen | Luminex | NR | 0.53 (0.23, 0.72) | 0.62 (0.20, 0.59) | 0.51 (0.33, 0.70) |
| CFHR2 | Chen, X; 2023 (37) | China | Bone | negative | 30 | OD | 30 | Serum, Frozen | ELISA | 86.90 μg/ml | 0.80 (0.63, 0.90) | 0.73 (0.56, 0.86) | 0.86 (0.77, 0.95) |
| CFHR3 | Chen, X; 2023 (37) | China | Bone | negative | 30 | OD | 30 | Serum, Frozen | ELISA | 138.25 μg/ml | 0.77 (0.59, 0.88) | 0.77 (0.59, 0.88) | 0.82 (0.70, 0.93) |
| CFHR5 | Chen, X; 2023 (37) | China | Bone | negative | 30 | OD | 30 | Serum, Frozen | ELISA | 2.67 μg/ml | **0.97 (0.83, 0.99)** | **0.97 (0.83, 0.99)** | 0.99 (0.99, 1.0) |
| CRP | Andrade, B; 2013 (25) | India | Pleural, Lymph node | negative | 35 | PTB | 97 | Plasma,  NR | Luminex | NR | 0.75 (0.65, 0.83) | 0.39 (0.23, 0.58) | 0.62 (0.52, 0.72) |
|  | He, X; 2020 (89) | China | Abdominal | negative | 73 | OD + HC | 135 | Serum,  NR | Turbidimetry | 43.54 IU/mL | 0.68 (0.57, 0.79) | 0.85 (0.78, 0.91) | 0.80 (0.74, 0.87) |
|  | Lin, L; 2021 (42) | China | Pleural | negative | 28 | OD | 16 | Serum,  NR | Turbidimetry | ≤ 77.1 mg/L | 0.75 (0.55, 0.89) | 0.88 (0.62, 0.98) | 0.84 (0.70, 0.93) |
|  | Lin, L; 2021 (42) | China | Pleural | negative | 28 | OD | 7 | Serum,  NR | Turbidimetry | > 19.3 mg/L | 0.75 (0.55, 0.89) | 1 (0.59, 1.0) | 0.91 (0.76, 0.98) |
|  | Mann, T; 2021 (44) | South Africa | Bone | positive and negative | 26 | OD | 17 | Serum,  Frozen | Luminex | 79091 ng/mL | **0.89 (0.70, 0.98)** | **0.94 (0.71, 0.99)** | 0.95 (0.87, 1.0) |
|  | Kim, J; 2016 (90) | South Korea | Meningeal | negative | 26 | OD | 70 | Serum,  NR | NR | NR | NR | NR | 0.78 (0.69, 0.86) |
|  | Wang, J; 2022 (93) | China | Bone | NR | 570 | OD | 147 | Serum,  NR | NR | NR | NR | NR | 0.73 (0.67, 0.79) |
| Ferritin | Mann, T; 2021 (44) | South Africa | Bone | positive and negative | 26 | OD | 17 | Serum,  Frozen | Luminex | NR | 0.69 (0.48, 0.86) | 0.82 (0.82, 0.96) | 0.78 (0.64, 0.92) |
| Fibrinogen | Mann, T; 2021 (44) | South Africa | Bone | positive and negative | 26 | OD | 17 | Serum,  Frozen | Luminex | 3696 ng/mL | **1 (0.89, 1.0)** | **0.94 (0.71, 0.99)** | 0.99 (0.96, 1.0) |
| GBP1 | Garlant, H; 2022 (21) | India, United Kingdom | NR | negative | 82 | LTBI | 111 | Whole blood, Frozen | ELISA | 1394 ng/mL | 0.90 (0.83, 0.95) | 0.36 (0.28, 0.45) | 0.78 (0.72, 0.84) |
| GDF-15 | Mann, T; 2021 (44) | South Africa | Bone | positive and negative | 26 | OD | 17 | Serum,  Frozen | Luminex | NR | 0.62 (0.41, 0.80) | 0.71 (0.44, 0.90) | 0.64 (0.48, 0.81) |
| HO-1 | Andrade, B; 2013 (25) | India | Pleural, Lymph nodes | negative | 35 | PTB | 97 | Plasma,  NR | ELISA | NR | 0.74 (0.64, 0.83) | 0.53 (0.34, 0.72) | 0.73 (0.65, 0.82) |
| I-309 | Mann, T; 2021 (44) | South Africa | Bone | positive and negative | 26 | OD | 17 | Serum,  Frozen | Luminex | NR | 0.73 (0.52, 0.88) | 0.65 (0.38, 0.86) | 0.74 (0.59, 0.90) |
| ICAM-1 | Mann, T; 2021 (44) | South Africa | Bone | positive and negative | 26 | OD | 17 | Serum,  Frozen | Luminex | NR | 0.58 (0.37, 0.77) | 0.71 (0.44, 0.90) | 0.60 (0.41, 0.78) |
| IFIT3 | Garlant, H; 2022 (21) | India, United Kingdom | NR | negative | 82 | LTBI | 111 | Whole blood, Frozen | ELISA | 14.43 ng/mL | **0.90 (0.83, 0.95)** | **0.89 (0.83, 0.94)** | 0.94 (0.90, 0.97) |
| IFITM3 | Garlant, H; 2022 (21) | India, United Kingdom | NR | negative | 82 | LTBI | 111 | Whole blood, Frozen | ELISA | 1303 ng/mL | **0.90 (0.83, 0.95)** | **0.71 (0.62, 0.78)** | 0.91 (0.88, 0.95) |
| IFN-γ | Abhimanyu, BM; 2016 (87) | India | Lymph node | negative | 50 | PTB | 84 | Serum, Frozen | ELISA | NR | NR | NR | 0.49 (0.39, 0.59) |
|  | de Larrea, C; 2011 (88) | Venezuela | Pleural | negative | 20 | OD | 40 | Serum,  NR | ELISA | 0.162 pg/mL | 0.65 (0.44, 0.90) | 0.55 (0.40, 0.70) | NR |
|  | Goyal, N; 2016 (45) | India | Pleural, lymph node, CSF, urine, ascitic fluid | negative | 69 | HC | 69 | Serum,  Frozen | ELISA | 11.4 pg/mL | **0.99 (0.92, 1.0)** | **0.97 (0.90, 1.0)** | 0.99 (0.97, 1.0) |
|  | Mann, T; 2021 (44) | South Africa | Bone | positive and negative | 26 | OD | 17 | Serum,  Frozen | Luminex | 8.56 pg/mL | **0.92 (0.75, 0.99)** | **0.94 (0.71, 0.99)** | 0.92 (0.83, 1.0) |
| IL-1β | Abhimanyu, BM; 2016 (87) | India | Lymph node | negative | 50 | PTB | 84 | Serum, Frozen | ELISA | NR | NR | NR | 0.55 (0.44, 0.65) |
| IL-1RA | Abhimanyu, BM; 2016 (87) | India | Lymph node | negative | 50 | PTB | 84 | Serum, Frozen | ELISA | NR | NR | NR | 0.43 (0.34, 0.52) |
| IL-2 | Abhimanyu, BM; 2016 (87) | India | Lymph node | negative | 50 | PTB | 84 | Serum, Frozen | ELISA | NR | NR | NR | 0.58 (0.48, 0.68) |
|  | Goyal, N; 2016 (45) | India | Pleural, lymph node, CSF, urine, ascitic fluid | negative | 69 | HC | 89 | Serum, Frozen | ELISA | NR | NR | NR | 0.50 (0.42, 0.59) |
| IL-2R | Onur, S; 2015 (43) | Turkey | Pleural | negative | 52 | OD | 68 | Serum,  Frozen | ELISA | > 0.6 ng/mL | 0.83 (0.69, 0.92) | 0.53 (0.40, 0.65) | NR |
| IL-4 | Abhimanyu, BM; 2016 (87) | India | Lymph node | negative | 50 | PTB | 84 | Serum, Frozen | ELISA | NR | NR | NR | 0.38 (0.28, 0.47) |
| IL-6 | Abhimanyu, BM; 2016 (87) | India | Lymph node | negative | 50 | PTB | 84 | Serum, Frozen | ELISA | NR | NR | NR | 0.58 (0.49, 0.67) |
| IL-8 | Abhimanyu, BM; 2016 (87) | India | Lymph node | negative | 50 | PTB | 84 | Serum, Frozen | ELISA | NR | NR | NR | 0.79 (0.72, 0.86) |
|  | Mann, T; 2021 (44) | South Africa | Bone | positive and negative | 26 | OD | 17 | Serum,  Frozen | Luminex | NR | 0.65 (0.38, 0.86) | 0.62 (0.41, 0.80) | 0.59 (0.41, 0.78) |
| IL-10 | Abhimanyu, BM; 2016 (87) | India | Lymph node | negative | 50 | PTB | 84 | Serum, Frozen | ELISA | NR | NR | NR | 0.74 (0.64, 0.85) |
|  | Mann, T; 2021 (44) | South Africa | Bone | positive and negative | 26 | OD | 17 | Serum,  Frozen | Luminex | NR | 0.58 (0.37, 0.77) | 0.77 (0.50, 0.93) | 0.68 (0.53, 0.82) |
| IL-12 | Abhimanyu, BM; 2016 (87) | India | Lymph node | negative | 50 | PTB | 84 | Serum, Frozen | ELISA | NR | NR | NR | 0.76 (0.69, 0.83) |
| IL-12p40 | de Larrea, C; 2011 (88) | Venezuela | Pleural | negative | 20 | OD | 40 | Serum,  NR | ELISA | 0.497 pg/mL | 0.95 (0.86, 1.05) | 0.50 (0.35, 0.66) | NR |
|  | Onur, S; 2015 (43) | Turkey | Pleural | negative | 52 | OD | 68 | Serum,  Frozen | ELISA | > 42 pg/mL | 0.81 (0.68, 0.90) | 0.40 (0.28, 0.52) | NR |
| IL-18 | Abhimanyu, BM; 2016 (87) | India | Lymph node | negative | 50 | PTB | 84 | Serum, Frozen | ELISA | NR | NR | NR | 0.58 (0.47, 0.69) |
| IP-10 | Mann, T; 2021 (44) | South Africa | Bone | positive and negative | 26 | OD | 17 | Serum,  Frozen | Luminex | NR | 0.62 (0.41, 0.79) | 0.71 (0.44, 0.90) | 0.63 (0.46, 0.80) |
| ITIH2 | Chen, X; 2023 (37) | China | Bone | negative | 30 | OD | 30 | Serum, Frozen | ELISA | 99.85 ng/mL | 0.73 (0.56, 0.86) | 0.67 (0.49, 0.81) | 0.72 (0.58, 0.85) |
| KNG1 | Chen, X; 2023 (37) | China | Bone | negative | 30 | OD | 30 | Serum, Frozen | ELISA | 157.32 μg/ml | 0.63 (0.46, 0.78) | 0.60 (0.42, 0.75) | NR |
| LBP | Lou, C; 2022 (92) | China | Bone | negative | 50 | HC | 30 | Serum, Frozen | ELISA | NR | NR | NR | 0.88 (NR) |
| LTA | Abhimanyu, BM; 2016 (87) | India | Lymph node | negative | 50 | PTB | 84 | Serum, Frozen | ELISA | NR | NR | NR | 0.61 (0.52, 0.69) |
| MIG | Mann, T; 2021 (44) | South Africa | Bone | positive and negative | 26 | OD | 17 | Serum,  Frozen | Luminex | NR | 0.58 (0.37, 0.77) | 0.88 (0.64, 0.99) | 0.62 (0.44, 0.79) |
| MMP-1 | Kathamuthu, G; 2020 (29) | India | Cervical lymphadenopathy | negative | 44 | LTBI | 44 | Plasma,  NR | Luminex | NR | 0.59 (0.43, 0.74) | 0.70 (0.55, 0.83) | 0.68 (NR) |
| MMP-12 | Kathamuthu, G; 2020 (29) | India | Cervical lymphadenopathy | negative | 44 | LTBI | 44 | Plasma,  NR | Luminex | NR | 0.52 (0.37, 0.68) | 0.55 (0.39, 0.69) | 0.50 (NR) |
| MMP-13 | Kathamuthu, G; 2020 (29) | India | Cervical lymphadenopathy | negative | 44 | LTBI | 44 | Plasma,  NR | Luminex | NR | **1.0 (0.92, 1.0)** | **1.0 (0.92, 1.0)** | 1.0 (NR) |
| MMP-2 | Kathamuthu, G; 2020 (29) | India | Cervical lymphadenopathy | negative | 44 | LTBI | 44 | Plasma,  NR | Luminex | NR | 0.61 (0.46, 0.76) | 0.65 (0.50, 0.79) | 0.62 (NR) |
| MMP-3 | Kathamuthu, G; 2020 (29) | India | Cervical lymphadenopathy | negative | 44 | LTBI | 44 | Plasma,  NR | Luminex | NR | 0.57 (0.41, 0.72) | 0.55 (0.39, 0.69) | 0.56 (NR) |
| MMP-7 | Kathamuthu, G; 2020 (29) | India | Cervical lymphadenopathy | negative | 44 | LTBI | 44 | Plasma,  NR | Luminex | NR | 0.75 (0.60, 0.87) | 0.70 (0.55, 0.83) | 0.80 (NR) |
| MMP-8 | Kathamuthu, G; 2020 (29) | India | Cervical lymphadenopathy | negative | 44 | LTBI | 44 | Plasma,  NR | Luminex | NR | 0.66 (0.50, 0.79) | 0.55 (0.39, 0.69) | 0.66 (NR) |
| MMP-9 | Kathamuthu, G; 2020 (29) | India | Cervical lymphadenopathy | negative | 44 | LTBI | 44 | Plasma,  NR | Luminex | NR | 0.50 (0.35, 0.65) | 0.55 (0.39, 0.69) | 0.59 (NR) |
| MPO | Mann, T; 2021 (44) | South Africa | Bone | positive and negative | 26 | OD | 17 | Serum,  Frozen | Luminex | NR | 0.58 (0.37, 0.77) | 0.76 (0.44, 0.90) | 0.60 (0.43, 0.80) |
| NCAM-1 | Mann, T; 2021 (44) | South Africa | Bone | positive and negative | 26 | OD | 17 | Serum,  Frozen | Luminex | 299421 pg/mL | 0.77 (0.56, 0.91) | 0.88 (0.64, 0.98) | 0.88 (0.78, 0.98) |
| OPG | Mann, T; 2021 (44) | South Africa | Bone | positive and negative | 26 | OD | 17 | Serum,  Frozen | Luminex | NR | 0.54 (0.27, 0.67) | 0.65 (0.14, 0.62) | 0.56 (0.38, 0.74) |
| PCT | Kim, J; 2016 (90) | South Korea | Meningeal | negative | 26 | OD | 70 | Serum,  NR | Electroche- miluminescent Immunoassay | ≤ 1.27 ng/mL | 0.96 (0.80, 0.99) | 0.63 (0.51, 0.74) | 0.84 (0.75, 0.90) |
| PD-L1 | Garlant, H; 2022 (21) | India, United Kingdom | NR | negative | 49 | LTBI | 111 | Whole blood, Frozen | ELISA | 0.5756 ng/mL | **1.0 (0.96, 1.0)** | **1.0 (0.97, 1.0)** | 1.0 (1.0, 1.0) |
| SAA | Andrade, B; 2013 (25) | India | Pleural, Lymph node | negative | 35 | PTB | 97 | Plasma,  NR | Luminex | NR | 0.79 (0.69, 0.87) | 0.39 (0.23, 0.58) | 0.66 (0.56, 0.77) |
| SAA-4 | Chen, X; 2023 (37) | China | Bone | negative | 30 | OD | 30 | Serum, Frozen | ELISA | 2.197 μg/ml | 0.73 (0.56, 0.86) | 0.70 (0.52, 0.83) | NR |
| SAMD9L | Garlant, H; 2022 (21) | India, United Kingdom | NR | negative | 82 | LTBI | 111 | Whole blood, Frozen | ELISA | 1439 ng/mL | **0.90 (0.83, 0.95)** | **0.88 (0.82, 0.93)** | 0.96 (0.94, 0.98) |
| SNX10 | Garlant, H; 2022 (21) | India, United Kingdom | NR | negative | 82 | LTBI | 111 | Whole blood, Frozen | ELISA | 1739 ng/mL | 0.90 (0.83, 0.95) | 0.37 (0.30, 0.46) | 0.81 (0.76, 0.87) |
| TIMP-1 | Kathamuthu, G; 2020 (29) | India | Cervical lymphadenopathy | negative | 44 | LTBI | 44 | Plasma,  NR | Luminex | NR | 0.57 (0.41, 0.72) | 0.64 (0.48, 0.78) | 0.61 (NR) |
| TIMP-2 | Kathamuthu, G; 2020 (29) | India | Cervical lymphadenopathy | negative | 44 | LTBI | 44 | Plasma,  NR | Luminex | NR | 0.82 (0.67, 0.92) | 0.91 (0.78, 0.97) | 0.86 (NR) |
| TIMP-3 | Kathamuthu, G; 2020 (29) | India | Cervical lymphadenopathy | negative | 44 | LTBI | 44 | Plasma,  NR | Luminex | NR | 0.65 (0.48, 0.77) | 0.82 (0.67, 0.92) | 0.62 (NR) |
| TIMP-4 | Kathamuthu, G; 2020 (29) | India | Cervical lymphadenopathy | negative | 44 | LTBI | 44 | Plasma,  NR | Luminex | NR | 0.62 (0.46, 0.76) | 0.73 (0.57, 0.85) | 0.69 (NR) |
| TMEM49 | Garlant, H; 2022 (21) | India, United Kingdom | NR | negative | 82 | LTBI | 111 | Whole blood, Frozen | ELISA | 266.3 ng/mL | 0.90 (0.83, 0.95) | 0.0 (0.0, 0.3) | 0.42 (0.35, 0.50) |
| TNF | Abhimanyu, BM; 2016 (87) | India | Lymph node | negative | 50 | PTB | 84 | Serum, Frozen | ELISA | NR | NR | NR | 0.38 (0.28, 0.48) |
| VCAM-1 | Mann, T; 2021 (44) | South Africa | Bone | positive and negative | 26 | OD | 17 | Serum,  Frozen | Luminex | NR | 0.54 (0.33, 0.73) | 0.71 (0.44, 0.90) | 0.61 (0.44, 0.79) |
| VEGF | Mann, T; 2021 (44) | South Africa | Bone | positive and negative | 26 | OD | 17 | Serum,  Frozen | Luminex | NR | 0.50 (0.30, 0.70) | 0.88 (0.64, 0.99) | 0.63 (0.46, 0.80) |
| **Signatures** | | | | | | | | | | | | | |
| CFHR2, CFHR3 | Chen, X; 2023 (37) | China | Bone | negative | 30 | OD | 30 | Serum, Frozen | ELISA | NR | **0.90 (0.74, 0.97)** | **0.88 (0.70, 0.95)** | 0.94 (0.87, 0.98) |
| CRP, NCAM, Ferritin, IL-8, GDF-15 | Mann, T; 2021 (44) | South Africa | Bone | positive and negative | 26 | OD | 17 | Serum,  Frozen | Luminex | NR | **1 (0.89, 1.0)** | **1 (0.84, 1.0)** | 1 (1.0, 1.0) |

**Table S11: Biomarkers and signatures for childhood TB, by reference standard and HIV status**

Biomarkers meeting TPP criteria are indicated in bold

AUC=area under the curve, ELISA=enzyme-linked immunosorbent assay, HC= healthy controls, LTBI=latent TB infection, N=sample size, ORD=other respiratory diseases, NR=not reported

| **Biomarker** | **Study ID** | **Country** | **Site of disease** | **TB cases, N** | **Control group** | **Control group, N** | **Sample type** | **Testing method** | **Biomarker cut-off, unit** | **Sensitivity**  **(95% CI)** | **Specificity**  **(95% CI)** | **AUC (95%)** |
| --- | --- | --- | --- | --- | --- | --- | --- | --- | --- | --- | --- | --- |
| **MRS, HIV-negative** | | | | | | | | | | | | |
| IFN-γ | Kumar, NP; 2021a (41) | India | PTB | 36 | ORD | 46 | Plasma, Frozen | Luminex | NR | 0.63 (0.46, 0.79) | 0.73 (0.59, 0.86) | 0.78 (NR) |
| IL-2 | Kumar, NP; 2021a (41) | India | PTB | 36 | ORD | 46 | Plasma, Frozen | Luminex | NR | 0.69 (0.52, 0.84) | 0.60 (0.45, 0.75) | 0.70 (NR) |
| IL-17A | Kumar, NP; 2021a (41) | India | PTB | 36 | ORD | 46 | Plasma, Frozen | Luminex | NR | **1.0 (0.90, 1.0)** | **0.91 (0.79, 0.98)** | 0.98 (NR) |
| TNF | Kumar, NP; 2021a (41) | India | PTB | 36 | ORD | 46 | Plasma, Frozen | Luminex | NR | **0.97 (0.86, 0.99)** | **0.90 (0.76, 0.96)** | 0.98 (NR) |
| **MRS, HIV-mixed** | | | | | | | | | | | | |
| CRP | Jaganath, D; 2022 (40) | Uganda | PTB | 62 | ORD | 270 | Whole blood, Fresh | POC assay | 10 mg/L | 0.50 (0.37, 0.63) | 0.66 (0.60, 0.71) | 0.61 (0.53, 0.69) |
| CRP | Jaganath, D; 2022 (40) | Uganda | PTB | 62 | ORD | 270 | Whole blood, Fresh | POC assay | 5 mg/L | 0.57 (0.43, 0.69) | 0.57 (0.51, 0.63) | 0.61 (0.53, 0.69) |
| **CRS, HIV-negative** | | | | | | | | | | | | |
| A1At | Manyelo, C; 2019 (94) | South Africa | EPTB; Meningeal | 23 | OD | 24 | Serum, Frozen | Luminex | >17908.0 ng/mL | 0.61 (0.39, 0.80) | 0.58 (0.37, 0.78) | 0.59 (0.42, 0.76) |
| ADMTS13 | Manyelo, C; 2019 (94) | South Africa | EPTB; Meningeal | 23 | OD | 24 | Serum, Frozen | Luminex | < 962.3 ng/mL | 0.61 (0.36, 0.80) | 0.46 (0.26, 0.67) | 0.52 (0.35, 0.68) |
| Apo AI | Manyelo, C; 2019 (94) | South Africa | EPTB; Meningeal | 23 | OD | 24 | Serum, Frozen | Luminex | > 287512.0 ng/mL | 0.65 (0.43, 0.84) | 0.54 (0.33, 0.75) | 0.62 (0.46, 0.78) |
| Apo CIII | Manyelo, C; 2019 (94) | South Africa | EPTB; Meningeal | 23 | OD | 24 | Serum, Frozen | Luminex | > 114926.0 ng/mL | 0.87 (0.66, 0.97) | 0.63 (0.41, 0.81) | 0.71 (0.55, 0.87) |
| Aβ40 | Manyelo, C; 2019 (94) | South Africa | EPTB; Meningeal | 23 | OD | 24 | Serum, Frozen | Luminex | < 72.1 pg/mL | 1.0 (0.85, 1.0) | 0.08 (0.01, 0.27) | 0.54 (0.37, 0.71) |
| Aβ42 | Manyelo, C; 2019 (94) | South Africa | EPTB; Meningeal | 23 | OD | 24 | Serum, Frozen | Luminex | < 278.4 pg/mL | 0.74 (0.52, 0.89) | 0.42 (0.22, 0.63) | 0.58 (0.45, 0.72) |
| BDNF | Manyelo, C; 2019 (94) | South Africa | EPTB; Meningeal | 23 | OD | 24 | Serum, Frozen | Luminex | < 17211.0 pg/mL | 0.65 (0.43, 0.84) | 0.54 (0.33, 0.75) | 0.55 (0.38, 0.72) |
| C2 | Manyelo, C; 2019 (94) | South Africa | EPTB; Meningeal | 23 | OD | 24 | Serum, Frozen | Luminex | < 15990.0 ng/mL | 0.52 (0.31, 0.73) | 0.50 (0.29, 0.71) | 0.53 (0.36, 0.70) |
| C3 | Manyelo, C; 2019 (94) | South Africa | EPTB; Meningeal | 23 | OD | 24 | Serum, Frozen | Luminex | > 32056.0 pg/mL | 0.91 (0.72, 0.98) | 0.42 (0.22, 0.63) | 0.40 (0.23, 0.57) |
| C4 | Manyelo, C; 2019 (94) | South Africa | EPTB; Meningeal | 23 | OD | 24 | Serum, Frozen | Luminex | > 89484.0 ng/mL | 0.78 (0.56, 0.93) | 0.54 (0.33, 0.75) | 0.65 (0.49, 0.81) |
| C4b | Manyelo, C; 2019 (94) | South Africa | EPTB; Meningeal | 23 | OD | 24 | Serum, Frozen | Luminex | > 26285.0 ng/mL | 0.69 (0.47, 0.87) | 0.54 (0.33, 0.75) | 0.66 (0.51, 0.82) |
| C5 | Manyelo, C; 2019 (94) | South Africa | EPTB; Meningeal | 23 | OD | 24 | Serum, Frozen | Luminex | > 46742.0 ng/mL | 0.69 (0.47, 0.87) | 0.75 (0.53, 0.90) | 0.73 (0.58, 0.88) |
| C5a | Manyelo, C; 2019 (94) | South Africa | EPTB; Meningeal | 23 | OD | 24 | Serum, Frozen | Luminex | > 2660.0 pg/mL | 0.52 (0.31, 0.73) | 0.67 (0.45, 0.84) | 0.58 (0.41, 0.75) |
| C9 | Manyelo, C; 2019 (94) | South Africa | EPTB; Meningeal | 23 | OD | 24 | Serum, Frozen | Luminex | < 3502.0 ng/mL | 0.65 (0.43, 0.84) | 0.58 (0.37, 0.78) | 0.56 (0.39, 0.73) |
| CAMP | Manyelo, C; 2019 (94) | South Africa | EPTB; Meningeal | 23 | OD | 24 | Serum, Frozen | ELISA | > 0.4 | 0.61 (0.39, 0.80) | 0.35 (0.16, 0.57) | 0.49 (0.31, 0.66) |
| Cathepsin D | Manyelo, C; 2019 (94) | South Africa | EPTB; Meningeal | 23 | OD | 24 | Serum, Frozen | Luminex | < 459422.0 pg/mL | 0.61 (0.39, 0.80) | 0.54 (0.33, 0.75) | 0.57 (0.40, 0.74) |
| CD40L | Manyelo, C; 2019 (94) | South Africa | EPTB; Meningeal | 23 | OD | 24 | Serum, Frozen | Luminex | > 11151.0 pg/mL | 0.65 (0.43, 0.84) | 0.54 (0.33, 0.75) | 0.56 (0.39, 0.73) |
| CFD | Manyelo, C; 2019 (94) | South Africa | EPTB; Meningeal | 23 | OD | 24 | Serum, Frozen | Luminex | < 2393.0 ng/mL | 0.78 (0.56, 0.93) | 0.79 (0.58, 0.93) | 0.75 (0.59, 0.90) |
| CFH | Manyelo, C; 2019 (94) | South Africa | EPTB; Meningeal | 23 | OD | 24 | Serum, Frozen | Luminex | > 350185.0 ng/mL | 0.87 (0.66, 0.97) | 0.67 (0.45, 0.84) | 0.72 (0.57, 0.87) |
| CFI | Manyelo, C; 2019 (94) | South Africa | EPTB; Meningeal | 23 | OD | 24 | Serum, Frozen | Luminex | > 57835.0 ng/mL | 0.65 (0.43, 0.84) | 0.63 (0.41, 0.81) | 0.62 (0.45, 0.78) |
| CRP | Kashyap, B; 2020 (46) | India | PTB and EPTB; Pleural, Abdominal, Bone, Meningeal, empyema, lymphadenopathy, psoas abscess | 60 | HC | 30 | Serum, Frozen | ELISA | 6.32 mg/L | **0.97 (0.89, 0.99)** | **0.90 (0.74, 0.98)** | 0.99 (0.97, 1.00) |
|  | Manyelo, C; 2019 (94) | South Africa | EPTB; Meningeal | 23 | OD | 24 | Serum, Frozen | Luminex | > 80721.0 ng/mL | 0.87 (0.66, 0.97) | 0.33 (0.16, 0.55) | 0.56 (0.43, 0.69) |
| CXCL1 | Kumar, NP; 2021b (47) | India | PTB | 44 | ORD | 76 | Plasma, Frozen | Luminex | NR | **1.0 (0.92, 1.0)** | **0.93 (0.85, 0.98)** | 0.99 (0.99, 1.0) |
| D-dimer | Manyelo, C; 2019 (94) | South Africa | EPTB; Meningeal | 23 | OD | 24 | Serum, Frozen | Luminex | < 9451.0 ng/mL | 0.52 (0.31, 0.73) | 0.50 (0.29, 0.71) | 0.55 (0.38, 0.72) |
| Ferritin | Manyelo, C; 2019 (94) | South Africa | EPTB; Meningeal | 23 | OD | 24 | Serum, Frozen | Luminex | < 56314.0 pg/mL | 0.57 (0.35, 0.77) | 0.58 (0.37, 0.78) | 0.53 (0.36, 0.70) |
| G-CSF | Manyelo, C; 2019 (94) | South Africa | EPTB; Meningeal | 23 | OD | 24 | Serum, Frozen | Luminex | < 76.0 pg/mL | 0.65 (0.43, 0.84) | 0.71 (0.49, 0.87) | 0.72 (0.57, 0.86) |
| GDF-15 | Manyelo, C; 2019 (94) | South Africa | EPTB; Meningeal | 23 | OD | 24 | Serum, Frozen | Luminex | < 1.1 ng/mL | 0.61 (0.39, 0.80) | 0.54 (0.33, 0.75) | 0.56 (0.39, 0.73) |
| GDNF | Manyelo, C; 2019 (94) | South Africa | EPTB; Meningeal | 23 | OD | 24 | Serum, Frozen | Luminex | < 140.4 pg/mL | 0.52 (0.31, 0.73) | 0.42 (0.22, 0.63) | 0.51 (0.34, 0.67) |
| GM-CSF | Manyelo, C; 2019 (94) | South Africa | EPTB; Meningeal | 23 | OD | 24 | Serum, Frozen | Luminex | < 9.3 pg/mL | 1.0 (0.85, 1.0) | 0.17 (0.05, 0.37) | 0.57 (0.40, 0.73) |
| I-309 | Kumar, NP; 2021b (47) | India | PTB | 44 | ORD | 76 | Plasma, Frozen | Luminex | 20.99 pg/mL | **0.98 (0.88, 0.99)** | **0.99 (0.93, 1.0)** | 0.99 (0.98, 1.0) |
|  | Manyelo, C; 2019 (94) | South Africa | EPTB; Meningeal | 23 | OD | 24 | Serum, Frozen | Luminex | < 15.2 pg/mL | 0.52 (0.31, 0.73) | 0.50 (0.29, 0.71) | 0.52 (0.35, 0.69) |
| ICAM-1 | Manyelo, C; 2019 (94) | South Africa | EPTB; Meningeal | 23 | OD | 24 | Serum, Frozen | Luminex | < 224039.0 pg/mL | 0.57 (0.35, 0.77) | 0.50 (0.29, 0.71) | 0.57 (0.40, 0.72) |
| IFN-γ | Manyelo, C; 2019 (94) | South Africa | EPTB; Meningeal | 23 | OD | 24 | Serum, Frozen | Luminex | < 61.5 pg/mL | 0.87 (0.66, 0.92) | 0.21 (0.07, 0.42) | 0.51 (0.39, 0.63) |
| IL-1β | Manyelo, C; 2019 (94) | South Africa | EPTB; Meningeal | 23 | OD | 24 | Serum, Frozen | Luminex | < 8.3 pg/mL | 0.91 (0.72, 0.99) | 0.29 (0.13, 0.51) | 0.56 (0.43, 0.68) |
| IL-4 | Manyelo, C; 2019 (94) | South Africa | EPTB; Meningeal | 23 | OD | 24 | Serum, Frozen | Luminex | < 116.7 pg/mL | 0.78 (0.56, 0.93) | 0.63 (0.41, 0.81) | 0.78 (0.65, 0.91) |
| IL-6 | Manyelo, C; 2019 (94) | South Africa | EPTB; Meningeal | 23 | OD | 24 | Serum, Frozen | Luminex | < 8.0 pg/mL | 0.57 (0.35, 0.77) | 0.58 (0.37, 0.78) | 0.63 (0.47, 0.79) |
| IL-7 | Manyelo, C; 2019 (94) | South Africa | EPTB; Meningeal | 23 | OD | 24 | Serum, Frozen | Luminex | > 27.5 pg/mL | 0.97 (0.47, 0.87) | 0.50 (0.29, 0.71) | 0.64 (0.48, 0.80) |
| IL-8 | Manyelo, C; 2019 (94) | South Africa | EPTB; Meningeal | 23 | OD | 24 | Serum, Frozen | Luminex | < 42.1 pg/mL | 0.61 (0.39, 0.80) | 0.67 (0.45, 0.84) | 0.65 (0.49, 0.81) |
| IL-10 | Manyelo, C; 2019 (94) | South Africa | EPTB; Meningeal | 23 | OD | 24 | Serum, Frozen | Luminex | < 7.0 pg/mL | 0.96 (0.78, 0.99) | 0.54 (0.33, 0.75) | 0.70 (0.56, 0.85) |
| IL-12p40 | Manyelo, C; 2019 (94) | South Africa | EPTB; Meningeal | 23 | OD | 24 | Serum, Frozen | Luminex | < 620.1 pg/mL | 1 (0.85, 1.0) | 0.04 (0.01, 0.21) | 0.52 (0.35, 0.69) |
| IL-13 | Manyelo, C; 2019 (94) | South Africa | EPTB; Meningeal | 23 | OD | 24 | Serum, Frozen | Luminex | < 74.6 pg/mL | 0.57 (0.35, 0.77) | 0.46 (0.26, 0.67) | 0.53 (0.38, 0.69) |
| IL-17A | Manyelo, C; 2019 (94) | South Africa | EPTB; Meningeal | 23 | OD | 24 | Serum, Frozen | Luminex | < 11.3 pg/mL | 0.96 (0.78, 0.99) | 0.38 (0.19, 0.59) | 0.65 (0.53, 0.76) |
| IL-21 | Manyelo, C; 2019 (94) | South Africa | EPTB; Meningeal | 23 | OD | 24 | Serum, Frozen | Luminex | < 34.6 pg/mL | 0.96 (0.78, 0.99) | 0.21 (0.07, 0.42) | 0.55 (0.43, 0.67) |
| IP-10 | Kumar, NP; 2021b (47) | India | PTB | 44 | ORD | 76 | Plasma, Frozen | Luminex | NR | **0.90 (0.78, 0.98)** | **0.85 (0.74, 0.92)** | 0.95 (0.91, 0.99) |
|  | Manyelo, C; 2019 (94) | South Africa | EPTB; Meningeal | 23 | OD | 24 | Serum, Frozen | Luminex | < 57.2 pg/mL | 0.52 (0.31, 0.73) | 0.67 (0.45, 0.84) | 0.61 (0.44, 0.77) |
| MBL | Manyelo, C; 2019 (94) | South Africa | EPTB; Meningeal | 23 | OD | 24 | Serum, Frozen | Luminex | > 4522.0 ng/mL | 0.78 (0.56, 0.93) | 0.58 (0.37, 0.78) | 0.68 (0.52, 0.84) |
| MCP-1 | Manyelo, C; 2019 (94) | South Africa | EPTB; Meningeal | 23 | OD | 24 | Serum, Frozen | Luminex | < 327.3 pg/mL | 0.74 (0.52, 0.89) | 0.75 (0.533, 0.902) | 0.81 (0.69, 0.93) |
| MIG | Manyelo, C; 2019 (94) | South Africa | EPTB; Meningeal | 23 | OD | 24 | Serum, Frozen | Luminex | > 2114.0 pg/mL | 0.52 (0.31, 0.73) | 0.62 (0.41, 0.81) | 0.54 (0.38, 0.71) |
| MIP-1α | Manyelo, C; 2019 (94) | South Africa | EPTB; Meningeal | 23 | OD | 24 | Serum, Frozen | Luminex | < 48.9 pg/mL | 0.65 (0.43, 0.84) | 0.54 (0.33, 0.75) | 0.57 (0.41, 0.74) |
| MIP-1β | Manyelo, C; 2019 (94) | South Africa | EPTB; Meningeal | 23 | OD | 24 | Serum, Frozen | Luminex | < 334.3 pg/mL | 0.78 (0.56, 0.93) | 0.67 (0.45, 0.84) | 0.76 (0.62, 0.90) |
| MIP-4 | Manyelo, C; 2019 (94) | South Africa | EPTB; Meningeal | 23 | OD | 24 | Serum, Frozen | Luminex | > 187.7 ng/mL | 0.69 (0.47, 0.87) | 0.54 (0.33, 0.75) | 0.61 (0.44, 0.77) |
| MMP-1 | Manyelo, C; 2019 (94) | South Africa | EPTB; Meningeal | 23 | OD | 24 | Serum, Frozen | Luminex | > 4282.0 pg/mL | 0.61 (0.39, 0.80) | 0.54 (0.33, 0.75) | 0.66 (0.50, 0.81) |
| MMP-7 | Manyelo, C; 2019 (94) | South Africa | EPTB; Meningeal | 23 | OD | 24 | Serum, Frozen | Luminex | < 869.0 pg/mL | 0.61 (0.39, 0.80) | 0.63 (0.41, 0.81) | 0.61 (0.45, 0.78) |
| MMP-8 | Manyelo, C; 2019 (94) | South Africa | EPTB; Meningeal | 23 | OD | 24 | Serum, Frozen | Luminex | > 22769.0 pg/mL | 0.57 (0.35, 0.77) | 0.58 (0.37, 0.78) | 0.59 (0.41, 0.75) |
| MMP-9 | Manyelo, C; 2019 (94) | South Africa | EPTB; Meningeal | 23 | OD | 24 | Serum, Frozen | Luminex | > 189764.0 pg/mL | 0.57 (0.35, 0.77) | 0.58 (0.37, 0.78) | 0.57 (0.40, 0.74) |
| MPO | Manyelo, C; 2019 (94) | South Africa | EPTB; Meningeal | 23 | OD | 24 | Serum, Frozen | Luminex | > 4650000.0 pg/mL | 0.52 (0.31, 0.73) | 0.71 (0.49, 0.87) | 0.56 (0.39, 0.73) |
| Myoglobin | Manyelo, C; 2019 (94) | South Africa | EPTB; Meningeal | 23 | OD | 24 | Serum, Frozen | Luminex | < 10.2 pg/mL | 0.61 (0.39, 0.80) | 0.67 (0.45, 0.84) | 0.61 (0.44, 0.78) |
| NCAM-1 | Manyelo, C; 2019 (94) | South Africa | EPTB; Meningeal | 23 | OD | 24 | Serum, Frozen | Luminex | < 264419.0 pg/mL | 0.69 (0.47, 0.87) | 0.71 (0.49, 0.87) | 0.68 (0.52, 0.84) |
| NGAL | Manyelo, C; 2019 (94) | South Africa | EPTB; Meningeal | 23 | OD | 24 | Serum, Frozen | Luminex | > 371.5 ng/mL | 0.52 (0.31, 0.73) | 0.50 (0.29, 0.71) | 0.52 (0.35, 0.69) |
| PAI-1 | Manyelo, C; 2019 (94) | South Africa | EPTB; Meningeal | 23 | OD | 24 | Serum, Frozen | Luminex | > 255621.0 pg/mL | 0.78 (0.56, 0.93 | 0.58 (0.37, 0.78) | 0.70 (0.55, 0.85) |
| PDGF-AA | Manyelo, C; 2019 (94) | South Africa | EPTB; Meningeal | 23 | OD | 24 | Serum, Frozen | Luminex | > 6150.0 pg/mL | 0.69 (0.47, 0.87) | 0.50 (0.29, 0.71) | 0.61 (0.44, 0.77) |
| PDGF-AB/BB | Manyelo, C; 2019 (94) | South Africa | EPTB; Meningeal | 23 | OD | 24 | Serum, Frozen | Luminex | > 42307.0 pg/mL | 0.65 (0.43, 0.84) | 0.67 (0.45, 0.84) | 0.68 (0.53, 0.84) |
| PEDF | Manyelo, C; 2019 (94) | South Africa | EPTB; Meningeal | 23 | OD | 24 | Serum, Frozen | Luminex | > 21725.0 ng/mL | 0.52 (0.31, 0.73) | 0.54 (0.33, 0.75) | 0.53 (0.36, 0.70) |
| P-Selectin | Manyelo, C; 2019 (94) | South Africa | EPTB; Meningeal | 23 | OD | 24 | Serum, Frozen | Luminex | > 159.1 ng/mL | 0.65 (0.43, 0.84) | 0.63 (0.41, 0.81) | 0.58 (0.42, 0.75) |
| RAGE | Manyelo, C; 2019 (94) | South Africa | EPTB; Meningeal | 23 | OD | 24 | Serum, Frozen | Luminex | < 875.8 pg/mL | 0.74 (0.52, 0.90) | 0.50 (0.29, 0.71) | 0.64 (0.48, 0.80) |
| RANTES | Manyelo, C; 2019 (94) | South Africa | EPTB; Meningeal | 23 | OD | 24 | Serum, Frozen | Luminex | > 99016.0 pg/mL | 0.57 (0.35, 0.77) | 0.54 (0.33, 0.75) | 0.52 (0.35, 0.69) |
| S100B | Manyelo, C; 2019 (94) | South Africa | EPTB; Meningeal | 23 | OD | 24 | Serum, Frozen | Luminex | > 2772.0 pg/mL | 0.56 (0.31, 0.79) | 0.40 (0.19, 0.64) | 0.50 (0.34, 0.66) |
| SAA | Manyelo, C; 2019 (94) | South Africa | EPTB; Meningeal | 23 | OD | 24 | Serum, Frozen | Luminex | > 59894.0 ng/mL | 0.57 (0.35, 0.77) | 0.67 (0.45, 0.84) | 0.54 (0.37, 0.71) |
| SAP | Manyelo, C; 2019 (94) | South Africa | EPTB; Meningeal | 23 | OD | 24 | Serum, Frozen | Luminex | > 257478.0 ng/mL | 0.78 (0.56, 0.93) | 0.71 (0.49, 0.87) | 0.74 (0.59, 0.89) |
| TGF-α | Manyelo, C; 2019 (94) | South Africa | EPTB; Meningeal | 23 | OD | 24 | Serum, Frozen | Luminex | > 29.9 pg/mL | 0.69 (0.47, 0.87) | 0.54 (0.33, 0.75) | 0.64 (0.48, 0.80) |
| TNF | Manyelo, C; 2019 (94) | South Africa | EPTB; Meningeal | 23 | OD | 24 | Serum, Frozen | Luminex | < 12.9 pg/mL | 0.78 (0.56, 0.93) | 0.79 (0.58, 0.93) | 0.77 (0.62, 0.91) |
| VCAM-1 | Manyelo, C; 2019 (94) | South Africa | EPTB; Meningeal | 23 | OD | 24 | Serum, Frozen | Luminex | < 1580000.0 pg/mL | 0.78 (0.56, 0.93) | 0.67 (0.45, 0.84) | 0.82 (0.70, 0.94) |
| VEGF | Manyelo, C; 2019 (94) | South Africa | EPTB; Meningeal | 23 | OD | 24 | Serum, Frozen | Luminex | > 111.2 pg/mL | 0.78 (0.56, 0.93) | 0.54 (0.33, 0.75) | 0.62 (0.45, 0.78) |
| **Signatures** | | | | | | | | | | | | |
| CFD, Aβ42, IL-10 | Manyelo, C; 2019 (94) | South Africa | EPTB, Meningeal | 23 | OD | 24 | Serum, Frozen | Luminex | NR | 0.83 (0.61, 0.95) | 0.71 (0.49, 0.87) | 0.84 (0.73, 0.96) |
| I-309, CXCL1 | Kumar, NP; 2021b (47) | India | PTB, CRS | 44 | ORD | 76 | Plasma, Frozen | Luminex | NR | **1.0 (0.92, 1.0)** | **1.0 (0.95, 1.0)** | 1.0 NR |
| CRP, IFN-γ, IP-10, CFH, Apo-AI, SAA, NCAM1 | Manyelo, C; 2019 (94) | South Africa | EPTB, Meningeal | 23 | OD | 24 | Serum, Frozen | Luminex | NR | 0.61 (0.39, 0.80) | 0.58 (0.37, 0.78) | 0.80 (0.67, 0.92) |
| TNF, IL-2, IL-17A | Kumar, NP; 2021a (41) | India | PTB, MRS | 36 | ORD | 46 | Plasma, Frozen | Luminex | NR | **1.0 (0.90, 1.0)** | **0.98 (0.89, 0.99)** | 0.99 (NR) |

**References**

17. Sahin F, Yıldız P. Distinctive biochemical changes in pulmonary tuberculosis and pneumonia. Arch Med Sci. 2013;9(4):656-61.

18. Yoon C, Semitala FC, Atuhumuza E, Katende J, Mwebe S, Asege L, et al. Point-of-care C-reactive protein-based tuberculosis screening for people living with HIV: a diagnostic accuracy study. The Lancet Infectious Diseases. 2017;17(12):1285-92.

19. Ruperez M, Shanaube K, Mureithi L, Wapamesa C, Burnett JM, Kosloff B, et al. Use of point-of-care C-reactive protein testing for screening of tuberculosis in the community in high-burden settings: a prospective, cross-sectional study in Zambia and South Africa. The Lancet Global Health. 2023;11(5):e704-e14.

20. Boyles TH, Nduna M, Pitsi T, Scott L, Fox MP, Maartens G. A Clinical Prediction Score Including Trial of Antibiotics and C-Reactive Protein to Improve the Diagnosis of Tuberculosis in Ambulatory People With HIV. Open Forum Infect Dis. 2020;7(2):ofz543.

21. Garlant HN, Ellappan K, Hewitt M, Perumal P, Pekeleke S, Wand N, et al. Evaluation of Host Protein Biomarkers by ELISA From Whole Lysed Peripheral Blood for Development of Diagnostic Tests for Active Tuberculosis. Frontiers in Immunology. 2022; 13:854327

22. Ndiaye MDB, Ranaivomanana P, Rasoloharimanana LT, Rasolofo V, Ratovoson R, Herindrainy P, et al. Plasma host protein signatures correlating with Mycobacterium tuberculosis activity prior to and during antituberculosis treatment. Scientific Reports. 2022;12(1):20640.

23. Mateos J, Estevez O, Gonzalez-Fernandez A, Anibarro L, Pallares A, Reljic R, et al. Serum proteomics of active tuberculosis patients and contacts reveals unique processes activated during Mycobacterium tuberculosis infection. Scientific Reports. 2020;10(1).

24. Kumar NP, Banurekha VV, Nair D, Dolla C, Kumaran P, Babu S. Modulation of iron status biomarkers in tuberculosis-diabetes co-morbidity. Tuberculosis (Edinb). 2018;108:127-35.

25. Andrade BB, Pavan Kumar N, Mayer-Barber KD, Barber DL, Sridhar R, Rekha VV, et al. Plasma heme oxygenase-1 levels distinguish latent or successfully treated human tuberculosis from active disease. PLoS One. 2013;8(5):e62618.

26. Andrade BB, Pavan Kumar N, Amaral EP, Riteau N, Mayer-Barber KD, Tosh KW, et al. Heme Oxygenase-1 Regulation of Matrix Metalloproteinase-1 Expression Underlies Distinct Disease Profiles in Tuberculosis. J Immunol. 2015;195(6):2763-73.

27. Shiratori B, Leano S, Nakajima C, Chagan-Yasutan H, Niki T, Ashino Y, et al. Elevated OPN, IP-10, and neutrophilia in loop-mediated isothermal amplification confirmed tuberculosis patients. Mediators Inflamm. 2014;2014:513263.

28. Franco Fontes C, Silva Bidu N, Rodrigues Freitas F, Maranhão RC, Santos Monteiro AdS, David Couto R, et al. Changes in serum amyloid A, plasma high-density lipoprotein cholesterol and apolipoprotein AI as useful biomarkers for Mycobacterium tuberculosis infection. J Med Microbiol. 2023 Jun;72(6).

29. Kathamuthu GR, Kumar NP, Moideen K, Nair D, Banurekha VV, Sridhar R, et al. Matrix Metalloproteinases and Tissue Inhibitors of Metalloproteinases Are Potential Biomarkers of Pulmonary and Extra-Pulmonary Tuberculosis. Front Immunol. 2020;11:419.

30. Liu YY, Ndumnego OC, Chen TT, Kim RS, Jenny-Avital ER, Ndung'u T, et al. Soluble CD14 as a Diagnostic Biomarker for Smear-Negative HIV-Associated Tuberculosis. Pathogens. 2018;7(1).

31. Wilson D, Badri M, Maartens G. Performance of Serum C-Reactive Protein as a Screening Test for Smear-Negative Tuberculosis in an Ambulatory High HIV Prevalence Population. PLOS ONE. 2011;6(1):e15248.

32. Namuganga AR, Nsereko M, Bagaya BS, Mayanja-Kizza H, Chegou NN. Differential expression of host protein biomarkers among symptomatic clinic attendees finally diagnosed with tuberculosis and other respiratory diseases with or without latent Mycobacterium tuberculosis infection. Immunology Letters. 2023;253:8-18.

33. Jacobs R, Malherbe S, Loxton AG, Stanley K, van der Spuy G, Walzl G, et al. Identification of novel host biomarkers in plasma as candidates for the immunodiagnosis of tuberculosis disease and monitoring of tuberculosis treatment response. Oncotarget. 2016;7(36):57581-92.

34. De Groote Mary A, Sterling David G, Hraha T, Russell Theresa M, Green Louis S, Wall K, et al. Discovery and Validation of a Six-Marker Serum Protein Signature for the Diagnosis of Active Pulmonary Tuberculosis. Journal of Clinical Microbiology. 2017;55(10):3057-71.

35. Morris TC, Hoggart CJ, Chegou NN, Kidd M, Oni T, Goliath R, et al. Evaluation of Host Serum Protein Biomarkers of Tuberculosis in sub-Saharan Africa. Front Immunol. 2021;12:639174.

36. Lubbers R, Sutherland JS, Goletti D, de Paus RA, van Moorsel CHM, Veltkamp M, et al. Complement Component C1q as Serum Biomarker to Detect Active Tuberculosis. Front Immunol. 2018;9:2427.

37. Chen X, Wang J, Wang J, Ye J, Di P, Dong C, et al. Several Potential Serum Proteomic Biomarkers for Diagnosis of Osteoarticular Tuberculosis Based on Mass Spectrometry. Clinica Chimica Acta. 2023:117447.

38. Sampath P, Rajamanickam A, Thiruvengadam K, Natarajan AP, Hissar S, Dhanapal M, et al. Cytokine upsurge among drug-resistant tuberculosis endorse the signatures of hyper inflammation and disease severity. Scientific Reports. 2023;13(1):785.

39. Koeppel L, Denkinger CM, Wyss R, Broger T, Chegou NN, Dunty JM, et al. Diagnostic performance of host protein signatures as a triage test for active pulmonary TB. J Clin Microbiol. 2023:e0026423.

40. Jaganath D, Reza TF, Wambi P, Nakafeero J, Kiconco E, Nanyonga G, et al. The Role of C-Reactive Protein as a Triage Tool for Pulmonary Tuberculosis in Children. J Pediatric Infect Dis Soc. 2022;11(7):316-21.

41. Kumar NP, Hissar S, Thiruvengadam K, Banurekha VV, Suresh N, Shankar J, et al. Discovery and Validation of a Three-Cytokine Plasma Signature as a Biomarker for Diagnosis of Pediatric Tuberculosis. Front Immunol. 2021;12:653898.

42. Lin L, Li S, Xiong Q, Wang H. A retrospective study on the combined biomarkers and ratios in serum and pleural fluid to distinguish the multiple types of pleural effusion. BMC Pulm Med. 2021;21(1):95.

43. Tural Önür S, Sökücü SN, Dalar L, Seyhan EC, Akbaş A, Altin S. Are soluble IL-2 receptor and IL-12p40 levels useful markers for diagnosis of tuberculous pleurisy? Infect Dis (Lond). 2015;47(3):150-5.

44. Mann TN, Davis JH, Walzl G, Beltran CG, du Toit J, Lamberts RP, et al. Candidate Biomarkers to Distinguish Spinal Tuberculosis From Mechanical Back Pain in a Tuberculosis Endemic Setting. Front Immunol. 2021;12:768040.

45. Goyal N, Kashyap B, Kaur IR. Significance of IFN-ɤ/IL-2 Ratio as a Circulating Diagnostic Biomarker in Extrapulmonary Tuberculosis. Scand J Immunol. 2016;83(5):338-44.

46. Kashyap B, Gupta N, Dewan P, Hyanki P, Singh NP. High Sensitivity C Reactive Protein: An Adjunct Diagnosis in Ruling Out Pediatric Tuberculosis. Indian J Clin Biochem. 2020;35(2):211-7.

47. Kumar NP, Hissar S, Thiruvengadam K, Banurekha VV, Balaji S, Elilarasi S, et al. Plasma chemokines as immune biomarkers for diagnosis of pediatric tuberculosis. Bmc Infectious Diseases. 2021;21(1).

48. Ciccacci F, Floridia M, Bernardini R, Sidumo Z, Mugunhe RJ, Andreotti M, et al. Plasma levels of CRP, neopterin and IP-10 in HIV-infected individuals with and without pulmonary tuberculosis. J Clin Tuberc Other Mycobact Dis. 2019;16:100107.

62. Calderwood CJ, Reeve BW, Mann T, Palmer Z, Nyawo G, Mishra H, et al. Clinical utility of C-reactive protein-based triage for presumptive pulmonary tuberculosis in South African adults. Journal of Infection. 2023;86(1):24-32.

63. Meyer AJ, Ochom E, Turimumahoro P, Byanyima P, Sanyu I, Lalitha R, et al. C-Reactive Protein Testing for Active Tuberculosis among Inpatients without HIV in Uganda: a Diagnostic Accuracy Study. J Clin Microbiol. 2020;59(1).

64. Samuels THA, Wyss R, Ongarello S, Moore DAJ, Schumacher SG, Denkinger CM. Evaluation of the diagnostic performance of laboratory-based c-reactive protein as a triage test for active pulmonary tuberculosis. PLOS ONE. 2021;16(7):e0254002.

65. Shapiro AE, Hong T, Govere S, Thulare H, Moosa MY, Dorasamy A, et al. C-reactive protein as a screening test for HIV-associated pulmonary tuberculosis prior to antiretroviral therapy in South Africa. Aids. 2018;32(13):1811-20.

66. Ahmad R, Xie L, Pyle M, Suarez MF, Broger T, Steinberg D, et al. A rapid triage test for active pulmonary tuberculosis in adult patients with persistent cough. Sci Transl Med. 2019;11(515).

67. Chen T, Lin J, Wang W, Fleming J, Chen L, Wang Y, et al. Cytokine and Antibody Based Diagnostic Algorithms for Sputum Culture-Positive Pulmonary Tuberculosis. PLoS One. 2015;10(12):e0144705.

68. Du ZX, Liang MM, Sun J, Wang WJ, Liu YH, Yang JH. Clinical significance of serum CA-125, CA19-9 and CEA in pulmonary tuberculosis with and without type 2 diabetes. Tuberculosis (Edinb). 2017;107:104-10.

69. Essone PN, Adegbite BR, Mbadinga MJM, Mbouna AV, Lotola-Mougeni F, Alabi A, et al. Creatine kinase-(MB) and hepcidin as candidate biomarkers for early diagnosis of pulmonary tuberculosis: a proof-of-concept study in Lambarene, Gabon. Infection. 2022 Aug;50(4):897-905.

70. Estévez O, Anibarro L, Garet E, Pallares Á, Pena A, Villaverde C, et al. Identification of candidate host serum and saliva biomarkers for a better diagnosis of active and latent tuberculosis infection. PLoS One. 2020;15(7):e0235859.

71. Farr K, Ravindran R, Strnad L, Chang E, Chaisson LH, Yoon C, et al. Diagnostic performance of blood inflammatory markers for tuberculosis screening in people living with HIV. PLoS One. 2018;13(10):e0206119.

72. Halliday A, Jain P, Hoang L, Parker R, Tolosa-Wright M, Masonou T, et al. Efficacy and Mechanism Evaluation. New technologies for diagnosing active TB: the VANTDET diagnostic accuracy study. NIHR Journals Library 2021. DOI: 10.3310/eme08050

73. Jiang XX, Huang JF, Huo Z, Zhang QQ, Jiang Y, Wu XP, et al. Elevation of soluble major histocompatibility complex class I related chain A protein in malignant and infectious diseases in Chinese patients. Bmc Immunology. 2012;13.

74. Kumar NP, Moideen K, Nancy A, Viswanathan V, Shruthi BS, Sivakumar S, et al. Plasma chemokines are biomarkers of disease severity, higher bacterial burden and delayed sputum culture conversion in pulmonary tuberculosis. Scientific Reports. 2019;9(1):18217.

75. Lawn SD, Kerkhoff AD, Vogt M, Wood R. Diagnostic and prognostic value of serum C-reactive protein for screening for HIV-associated tuberculosis. Int J Tuberc Lung Dis. 2013;17(5):636-43.

76. Lee K, Chung W, Jung Y, Kim Y, Park J, Sheen S, et al. CXCR3 ligands as clinical markers for pulmonary tuberculosis. Int J Tuberc Lung Dis. 2015;19(2):191-9.

77. Lubbers R, Sutherland JS, Goletti D, de Paus RA, Dijkstra DJ, van Moorsel CHM, et al. Expression and production of the SERPING1-encoded endogenous complement regulator C1-inhibitor in multiple cohorts of tuberculosis patients. Mol Immunol. 2020;120:187-95.

78. Luo Y, Xue Y, Lin Q, Tang G, Yuan X, Mao L, et al. A combination of iron metabolism indexes and tuberculosis-specific antigen/phytohemagglutinin ratio for distinguishing active tuberculosis from latent tuberculosis infection. Int J Infect Dis. 2020;97:190-6.

79. Mikačić M, Vasilj I, Vasilj M, Bevanda D, Šimović M, Galić K. Tumor Marker CA 125 in the Diagnosis of Active Pulmonary Tuberculosis - A Study of Adults in Mostar, B&H. Psychiatr Danub. 2017;29 Suppl 4(Suppl 4):841-4.

80. Moreira FMF, Verma R, dos Santos PCP, Leite A, Santos AD, de Araujo RCP, et al. Blood-based host biomarker diagnostics in active case finding for pulmonary tuberculosis: A diagnostic case-control study. EClinicalMedicine. 2021;33.

81. Namuganga AR, Chegou NN, Mubiri P, Walzl G, Mayanja-Kizza H. Suitability of saliva for Tuberculosis diagnosis: comparing with serum. BMC Infect Dis. 2017;17(1):600.

82. Peruhype-Magalhães V, de Araújo FF, de Morais Papini TF, Wendling APB, Campi-Azevedo AC, Coelho-dos-Reis JG, et al. Serum biomarkers in patients with unilateral or bilateral active pulmonary tuberculosis: Immunological networks and promising diagnostic applications. Cytokine. 2023;162:156076.

83. Sampath P, Rajamanickam A, Thiruvengadam K, Natarajan AP, Hissar S, Dhanapal M, et al. Plasma chemokines CXCL10 and CXCL9 as potential diagnostic markers of drug-sensitive and drug-resistant tuberculosis. Scientific Reports. 2023;13(1):7404.

84. Uwimaana E, Bagaya BS, Castelnuovo B, Kateete DP, Godwin A, Kiwanuka N, et al. Heme oxygenase-1 and neopterin plasma/serum levels and their role in diagnosing active and latent TB among HIV/TB co-infected patients: a cross sectional study. Bmc Infectious Diseases. 2021;21(1).

85. Yang Q, Cai Y, Zhao W, Wu F, Zhang M, Luo K, et al. IP-10 and MIG are compartmentalized at the site of disease during pleural and meningeal tuberculosis and are decreased after antituberculosis treatment. Clin Vaccine Immunol. 2014;21(12):1635-44.

86. Boyles TH, Nduna M, Pitsi T, Scott L, Fox MP, Maartens G, editors. A clinical prediction score including trial of antibiotics and C-reactive protein to improve the diagnosis of tuberculosis in ambulatory people with HIV. Open Forum Infect Dis. 2020 Jan 6;7(2):ofz543

87. Abhimanyu, Bose M, Varma-Basil M, Jain A, Sethi T, Tiwari PK, et al. Establishment of Elevated Serum Levels of IL-10, IL-8 and TNF-β as Potential Peripheral Blood Biomarkers in Tubercular Lymphadenitis: A Prospective Observational Cohort Study. PLoS One. 2016;11(1):e0145576.

88. Fernández de Larrea C, Duplat A, Giampietro F, de Waard JH, Luna J, Singh M, et al. Diagnostic accuracy of immunological methods in patients with tuberculous pleural effusion from Venezuela. Invest Clin. 2011;52(1):23-34.

89. He X, Gao Y, Liu Q, Zhao Z, Deng W, Yang H. Diagnostic Value of Interferon-Gamma Release Assays Combined with Multiple Indicators for Tuberculous Peritonitis. Gastroenterol Res Pract. 2020;2020:2056168.

90. Kim J, Kim SE, Park BS, Shin KJ, Ha SY, Park J, et al. Procalcitonin as a Diagnostic and Prognostic Factor for Tuberculosis Meningitis. Journal of Clinical Neurology. 2016;12(3):332-9.

91. Liu Q, Ou Q, Chen H, Gao Y, Liu Y, Xu Y, et al. Differential expression and predictive value of monocyte scavenger receptor CD163 in populations with different tuberculosis infection statuses. BMC Infect Dis. 2019;19(1):1006.

92. Lou C, Liu J, Ren Z, Ji J, Ma H, Dong H, et al. Analysis of the Value of Serum Biomarker LBP in the Diagnosis of Spinal Tuberculosis. Infection and Drug Resistance. 2022:4915-26.

93. Wang J, Feng Z-X, Ren T, Meng W-Y, Khan I, Fan X-X, et al. Novel clinical biomarkers in blood and pleural effusion for diagnosing patients with tuberculosis distinguishing from malignant tumor. Medicine. 2022;101(41):e31027.

94. Manyelo CM, Solomons RS, Snyders CI, Mutavhatsindi H, Manngo PM, Stanley K, et al. Potential of Host Serum Protein Biomarkers in the Diagnosis of Tuberculous Meningitis in Children. Front Pediatr. 2019;7:376.
